# Serum C-Reactive Protein and Periodontitis: A Systematic Review and Meta-Analysis

**DOI:** 10.3389/fimmu.2021.706432

**Published:** 2021-07-28

**Authors:** Vanessa Machado, João Botelho, Cláudia Escalda, Syed Basit Hussain, Shailly Luthra, Paulo Mascarenhas, Marco Orlandi, José João Mendes, Francesco D’Aiuto

**Affiliations:** ^1^Periodontology Department, Clinical Research Unit, Centro de Investigação Interdisciplinar Egas Moniz (CiiEM), Egas Moniz, Cooperativa de Ensino Superior, CRL, Almada, Portugal; ^2^Evidence Based Hub, Clinical Research Unit, Centro de Investigação Interdisciplinar Egas Moniz (CiiEM), Egas Moniz, Cooperativa de Ensino Superior, CRL, Almada, Portugal; ^3^Periodontology Unit, University College London (UCL) Eastman Dental Institute, London, United Kingdom

**Keywords:** inflammation, periodontal disease, periodontitis, periodontal medicine, systematic reviews and evidence-based medicine, oral-systemic disease(s), systemic health/disease, C- reactive protein

## Abstract

Periodontitis has been associated with low-grade inflammation as assessed by C-reactive protein (CRP) levels and its treatment can decrease CRP serum levels. The aim of this systematic review was to critically appraise the evidence comparing CRP serum levels (standard and high-sensitivity [hs]) of otherwise healthy patients suffering from periodontitis when compared to controls. The impact of intensive and non-intensive nonsurgical periodontal treatment (NSPT) on hs-CRP was also investigated. Four electronic databases (Pubmed, The Cochrane Central Register of Controlled Trials [CENTRAL], EMBASE and Web of Science) were searched up to February 2021 and the review was completed according to Preferred Reporting Items for Systematic Reviews and Meta-Analyses (PRISMA) guidelines (PROSPERO No. CRD42020167454). Observational and intervention studies that: 1) evaluated CRP and hs-CRP serum levels in patients with and without periodontitis, and; 2) hs- CRP levels after NSPT were included. Following risk of bias appraisal, both qualitative and quantitative analyses were performed. Pooled estimates were rendered through ratio of means (RoM) random-effects meta-analyses. After screening 485 studies, 77 case-control studies and 67 intervention trials were included. Chronic and aggressive periodontitis diagnoses were consistently associated with higher levels of CRP and hs-CRP (p<0.001). Patients with aggressive periodontitis exhibited on average more than 50% higher levels of CRP (RoM [95% confidence interval [CI]]: 1.56 [1.15; 2.12], p=0.0039) than patients with chronic periodontitis. Intensive NSPT induced an immediate increase of hs-CRP followed by a progressive decrease whilst non-intensive NSPT consistently decreased hs-CRP after treatment up to 180 days (p<0.001). These findings provide robust evidence that periodontitis is associated with systemic inflammation as measured by serum CRP levels. Periodontitis treatment induces a short-term acute inflammatory increase when performed in an intensive session, whilst a progressive reduction up to 6 months was demonstrated when performed in multiple visits.

## Introduction

Periodontitis is a chronic inflammatory disease initiated by a dysbiotic dental biofilm (1) and followed by a progressive destruction of periodontal tissues (2). Bacteria and their products progressively affect the periodontium integrity, which can trigger a local inflammatory response but also a systemic response (2, 3). Raised levels of pro-inflammatory mediators have been reported in patients with periodontitis (4) who also exhibit distinct hematological changes (5) including raised levels of C-reactive protein (CRP) (6).

CRP is an acute-phase protein mainly produced by the liver in response to infection or tissue damage (7, 8). While CRP is a more traditional inflammation marker with less precision (within the range of 10 to 1,000 mg/L), high sensitivity CRP (hs-CRP) is a highly precise and accurate analyte (within the range of 0.5 to 10 mg/L) (9). The association between CRP and periodontitis received great attention in part due to the link between periodontitis and cardiovascular disease (CVD) (6). However, CRP has been established as a marker of association of periodontitis with other systemic diseases (3).

Previous systematic reviews suggested elevated CRP levels in patients with periodontitis (6, 10–12). Nevertheless, there are inconsistent evidence-based outcomes on the effect of two different periodontal therapeutic approaches (13–19). Standard non-intensive periodontal therapy (NSPT) consists of conventional mechanical scaling and root planning with polishing on a quadrant-by-quadrant with local anesthesia. Whereas intensive periodontal therapy (IPT) involves an intensive session of full-mouth subgingival root debridement delivered within a 24-hour period under local anesthesia (where periodontal surgery, teeth removal or local antibiotic might be delivered). Given the clinical relevance of chronic inflammation and future systemic health outcomes, including in patients with periodontitis, the amount of newly available evidence, an updated and robust appraisal of the data on the association of periodontitis and its treatment with CRP levels was thought to be of interest.

The overall aim of the present systematic review was to critically appraise all up-to-date evidence on the impact of diagnosis of periodontitis on serum CRP levels in otherwise healthy patients when compared to controls. We further investigated the impact of non-intensive and intensive NSPT on the variations of hs-CRP levels after treatment.

## Materials and Methods

### Protocol and Registration

This systematic review protocol was previously defined and registered at the National Institute for Health Research PROSPERO, International Prospective Register of Systematic Review (CRD42020167454). The review was conducted based on the Cochrane Handbook of Systematic Reviews of Interventions (20) and reported according to the Preferred Reporting Items for Systematic Reviews and Meta-Analyses (PRISMA) guidelines (21) (**Supplementary Table 1**).

### Focused Questions and Eligibility Criteria

We developed a protocol to answer two PI(E)CO questions:

1. “In otherwise healthy individuals, do patients with periodontitis have increased serum CRP and hs-CRP levels compared to patients without periodontitis?”.
2. “Do hs-CRP levels decrease after intensive e non-intense NSPT?”

Each question had the following statements:

1. Otherwise healthy patients (Patients, P); Periodontitis (Exposure, E); Non-periodontitis (Comparison, C); CRP and hs-CRP serum levels (Outcome, O).
2. Otherwise healthy patients (Patients, P); Intensive and non-intensive NSPT (Intervention, I); Baseline serum levels of hs-CRP (Comparison, C); Posttreatment hs-CRP serum levels (Outcome, O).

Studies were eligible for inclusion based on the following criteria:

- Observational (cross-sectional, case-control) studies using serum CRP and hs-CRP levels of otherwise healthy participants with periodontitis and controls;
- Intervention studies (randomized controlled trials [RCTs] and non-randomized studies [NRSI]) reporting changes of hs-CRP levels after NSPT;
- Participants with periodontitis and no other comorbidities that could influence CRP serum levels, such as a history of CVD, atherosclerosis, diabetes mellitus, obesity, postmenopausal women;
- Studies with a clear case definition of periodontitis;
- Studies with clear reporting the CRP and hs-CRP method of quantification (for instance, Immunoturbidimetry, Nephelometry, Enzyme-Linked Immunosorbent Assay [ELISA], Chemiluminescent enzyme immunometric assay [CLIA], Radial immunodiffusion assay [RIA]).

### Search Strategy

Identification of studies was performed through detailed search strategies developed for each database (Pubmed, The Cochrane Central Register of Controlled Trials (CENTRAL), EMBASE, Web of Science) until February 2021. Our Pubmed search strategy was based on the following algorithm: (periodontitis OR gingivitis OR periodontal health OR (periodontal diseases [MeSH])) AND (C-reactive protein OR (C-reactive protein [MeSH]) OR CRP OR Acute Phase Protein OR high sensitivity C-reactive protein OR hs-CRP). No restrictions were applied regarding the publication year or language. Grey literature was searched through an appropriate database (http://www.opengrey.eu/). Hand searching in four periodontology journals (namely, Journal of Clinical Periodontology, Journal of Periodontology, Periodontology 2000, Journal of Periodontal Research) was conducted to search articles not found through other search methods.

### Study Selection

Study selection was assessed independently by three investigators (VM, JB and CE), who screened the titles and/or abstracts of retrieved studies. Any disagreements were resolved by discussion with a fourth author (SBH). For measurement reproducibility purposes, inter-examiner reliability following full-text assessment was calculated *via* kappa statistics.

### Data Extraction Process and Data Items

Electronic data were extracted to a predefined table, including the first author’s name, study design, publication year, country where the study was conducted, project funding, mean age, number of participants per group and by gender, percentage of smokers per group, periodontal case definition, CRP and hs-CRP method and serum levels (values were expressed in mg/L). For intervention studies, hs-CRP serum levels at baseline and after intensive and non-intensive NSPT were extracted. Three independent reviewers (VM, JB and CE) completed data extraction. Corresponding authors of studies were contacted if there were missing information.

The primary aim of this systematic review was to assess CRP and hs-CRP serum levels in patients with and without periodontitis (chronic or aggressive). The secondary aims included assessment of hs-CRP serum levels after NSPT comparing to baseline data according to the type of NSPT: 1) IPT; and, 2) non-intensive periodontal treatment.

### Risk of Bias (RoB) in Individual Studies

Case-control studies were appraised using the Newcastle-Ottawa Scale (NOS) by two calibrated reviewers (VM and CE). We considered studies with 7-9 stars as of low RoB, studies with 5-6 stars of moderate RoB, whilst studies with less than 5 stars were deemed of high RoB (5, 22). The Cochrane Collaboration’s tool RoB2 was used to assess RCTs (23) and the RoB in Non-randomized Studies - of Interventions (ROBINS-I) tool for non-RCT studies of intervention (24) by two calibrated examiners (JB and BSH). Any disagreement was resolved by discussion.

### Summary Measures and Synthesis of Results

For continuous data (serum levels of CRP and hs-CRP), mean values and standard deviations (SD) were used. The unit of measurement used in the meta-analysis was mg/L, and all studies reporting different units of measurement were converted appropriately. If median and interquartile range were reported in the selected studies, we converted to mean and SD following Hozo’s procedure (25).

Estimates were calculated in R version 3.4.1 (R Studio Team 2018) using a DerSimonian-Laird random-effects model (26). All random-effects meta-analysis and forest plots were performed using ‘meta’ package (27). Statistical heterogeneity was assessed through I^2^ index and Cochrane’s Q statistic (p<0.1), and the overall homogeneity was calculated through the χ2 test (28). All tests were two-tailed, with alpha set at 0.05. Further, the weight percentage given to each study in each analysis was provided in the forest plots. We analyzed publication bias if, at least, 10 or more studies were included (28). Overall estimates were reported with 95% confidence interval (CI).

We started by conducting an *a priori* sensitivity analysis comparing Standardized Mean Difference (SMD) versus Ratio of Means (RoM) meta-analyses. In case of similar results in terms of heterogeneity and significance, RoM was used as it would allow easier and direct interpretation of the results (reported as percentage) (29, 30).

According to the type of NSPT (intensive and non-intensive) delivered, a subgroup meta-analysis was made for each day after treatment. Then values were graphically assembled in a scatterplot using ‘ggplot2’ package, with mean and 95% CI values in a vertical interval. Post-hoc sensitivity analyses were designed to assess the impact of high RoB in the pooled estimates, as well as the influence of different study designs (intervention and case-control).

## Results

### Study Selection

**Figure 1:**
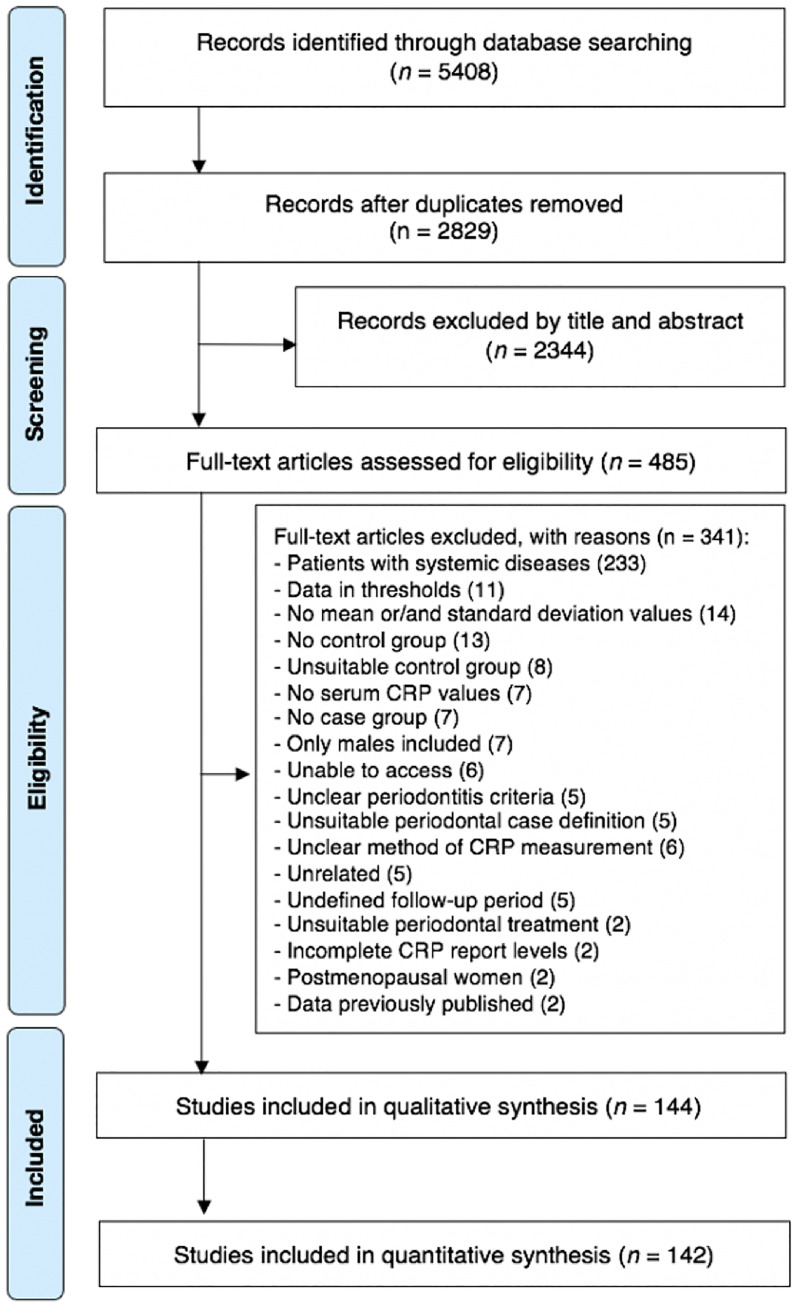
PRISMA flow-chart representing the results of the workflow to identify eligible studies.

The search strategy retrieved 5,408 possibly relevant articles. After duplicate removal, 2,829 manuscripts were screened against the eligibility criteria; 2344 were excluded after titles and/or abstracts review. Among these, 485 articles were assessed for full paper review eligibility, 341 were excluded (detailed reasons for exclusion in **Supplementary Table 2**). A total of 144 studies were included for qualitative synthesis whilst 142 for quantitative analyses (**Figure 1**). Good inter-examiner reliability was confirmed at the full-text screening (kappa score = 0.92, 95% CI: 0.89; 0.94).

### Characteristics of the Studies

In this systematic review, 77 case control-studies (Supplementary 3 - References) and 67 intervention studies (Supplementary 4 - References) were included. Twelve intervention studies had relevant information regarding the comparison of otherwise healthy patients with periodontitis and non-periodontitis (31–42) (Supplementary 4 – References). Two studies had their data reported in more than one article: thus, these two papers were grouped under a single name study as follows: Gani et al., 2009/2012 (43, 44). Overall, studies were performed in 27 countries across Asia, Europe, America and Oceania (**Supplementary Tables 5, 6**). A total of 11,242 participants in observational and 2,199 in intervention studies were included in the analysis. Among the observational studies 6,155 healthy participants and 5,087 patients with periodontitis were identified (4,319 diagnosed with chronic- and 768 with aggressive-periodontitis). Three studies reported data for combined diagnoses (chronic and aggressive) of periodontitis (45–47). Among the intervention studies, 1,389 participants received non-intensive whilst 810 received intensive NSPT.

A plethora of case definitions of periodontitis were retrieved (as shown in **Supplementary Tables 5, 6**) as well as variability in the used CRP and hs-CRP methods (i.e., immunoturbidimetry, nephelometry, ELISA, CLIA, RIA, Cardiac-specific claim, latex agglutination method and spectrophotometer) (**Supplementary Tables 5, 6**).

Seventy-one observational studies were classified as of low RoB according to the NOS for case-control studies (**Supplementary Table 7**) (28 with 9 stars, 29 with 8 stars and 14 with 7 stars). Good inter-examiner reliability was confirmed at the RoB assessment (kappa score = 0.94, 95% CI: 0.84; 1.00). Six observational studies had moderate RoB. Most articles failed in including a representative number of cases (43.0%, n=34), nevertheless, most of them had adequate cases (88.6%, n=70) and controls (94.9%, n=75) definition as well as selection (72.2%, n=57). All studies had excellent a) comparability results (100%, n=79) and b) values of non-response rate (98.7%, n=78). All studies reported a secure record of ascertainment of exposure with equal ascertainment method for cases and controls (100.0%, n=79).

Forty-four non-RCT had a low RoB, two had serious RoB and two had insufficient information according to the ROBINS-I tool (**Supplementary Table 8**). Lastly, fifteen RCTs had a low RoB according to ROB2 and four had some concerns regarding RoB (**Supplementary Figure 1**).

### Diagnosis of Periodontitis and CRP Levels

The *a priori* sensitivity analysis was performed to compare RoM and SMD approaches yielding similar results (**Supplementary Table 9**).

Eighteen (CRP) and fifty-nine (hs-CRP) studies were included (**Supplementary Figures 2–5**). Chronic periodontitis was associated with an increase of CRP and hs-CRP levels of 103% and 110%, respectively (p<0.0001) (RoM [95% CI]: 2.03 [1.59; 2.60], p<0.0001, *I^2^ =* 96.3% and RoM [95% CI]: 2.10 [1.62; 2.55], p<0.0001, *I^2^ =* 97.4%, respectively) (**Table 1**). Sensitivity analyses showed no differences in these results based on the type of study and CRP measurement method (**Supplementary Table 10** and **Supplementary Figures 2–5**). In particular, estimates from studies with low RoB demonstrated only mild improvement in heterogeneity (I^2^ = 66.3% vs I^2^ = 98.5% for all studies) (**Supplementary Figure 3**). No impact of level of RoB on heterogeneity was noted for studies reporting hs-CRP levels (**Supplementary Table 11**).

**Table 1:** CRP and hs-CRP levels according to the periodontal status.

Variable	N studies	N of participants	ROM	95% CI	p-value	I^2^ (%)	Tau	Egger test
**Healthy periodontium vs. Chronic Periodontitis**								
CRP (mg/L)	19	CP: 1411; H: 2894	**2.10**	1.56; 2.83	**<0.0001**	98.5	0.640	0.027
hs-CRP (mg//L)	59	CP: 2615; H: 2317	**2.04**	1.72; 2.43	**<0.0001**	99.8	0.399	0.083
**Healthy periodontium vs. Aggressive Periodontitis**								
CRP (mg/L)	7	AgP: 266; H: 243	**2.91**	1.91; 4.44	**<0.0001**	98.6	0.548	-
hs-CRP (mg//L)	6	AgP: 424; H: 270	**2.79**	1.68; 4.65	**<0.001**	83.6	0.547	-
**Healthy periodontium vs. PD (Chronic- and Aggressive- Periodontitis combined)**								
CRP (mg/L)	24	PD: 2062; H: 3305	**2.06**	1.64; 2.59	**<0.0001**	98.7	0.503	0.041
hs-CRP (mg//L)	65	PD: 2741; H: 2885	**2.09**	1.78; 2.47	**<0.0001**	99.8	0.401	0.064
**Chronic vs. Aggressive Periodontitis**								
CRP (mg/L)	5	CP: 145; AgP: 232	**1.56**	1.15; 2.12	**0.0039**	84.9	0.300	-
hs-CRP (mg//L)	5	CP: 101; AgP: 118	1.69	0.96; 2.98	0.0667	77.5	0.513	-

CRP, C-reactive protein; hs-CRP, high sensitivity CRP; ROM, Ratio of Mean. Bold values mean p value below 0.05.

A diagnosis of aggressive periodontitis was associated with an increase of CRP of 191% (RoM [95% CI]: 2.91 [1.91; 4.44], p<0.0001, *I^2^ =* 98.6%) and of hs-CRP of 179% (RoM [95% CI]: 2.79 [1.68; 4.65], p<0.0001, *I^2 ^=* 83.6%) when compared to controls (**Table 1**). Sensitivity analyses showed no differences in these results based on the type of study and CRP method of measurement (**Supplementary Figures 6, 7**).

Combined diagnosis of periodontitis was associated with an increase of 80% of CRP (RoM [95% CI]: 1.80 [1.36; 2.38], p<0.0001, *I^2^ =* 99.4%) and of 109% of hs-CRP (RoM [95% CI]: 2.09 [1.78; 2.47], p<0.0001, *I^2^ =* 99.8%) when compared to healthy controls (**Supplementary Figures 8–10**). When comparing different diagnoses, aggressive periodontitis was associated with 56% higher levels of CRP than chronic periodontitis (RoM [95% CI]: 1.56 [1.15; 2.12], p=0.0039, *I^2^ = * 84.9%) (**Supplementary Figures 11–13**).

Publication bias was noted in studies examining CRP levels in patients with periodontitis versus controls (p=0.041) as well as studies comparing the specific diagnosis of chronic periodontitis versus healthy controls (p=0.027) (**Supplementary Figures 14–17**).

### Periodontal Treatment and CRP Levels

All studies included reported hs-CRP results at baseline and after NSPT with data at 1, 7, 30, 60, 90 and 180 days of treatment delivered (non-intensive and intensive NSPT). An *a priori* sensitivity analysis confirmed the suitability of RoM meta-analysis, with statistical significance and heterogeneity similar across analyses (**Supplementary Tables 12, 13**). Sensitivity analyses of studies with low RoB confirmed the same findings of the overall analyses (**Supplementary Tables 14–16**).

**Figure 2:**
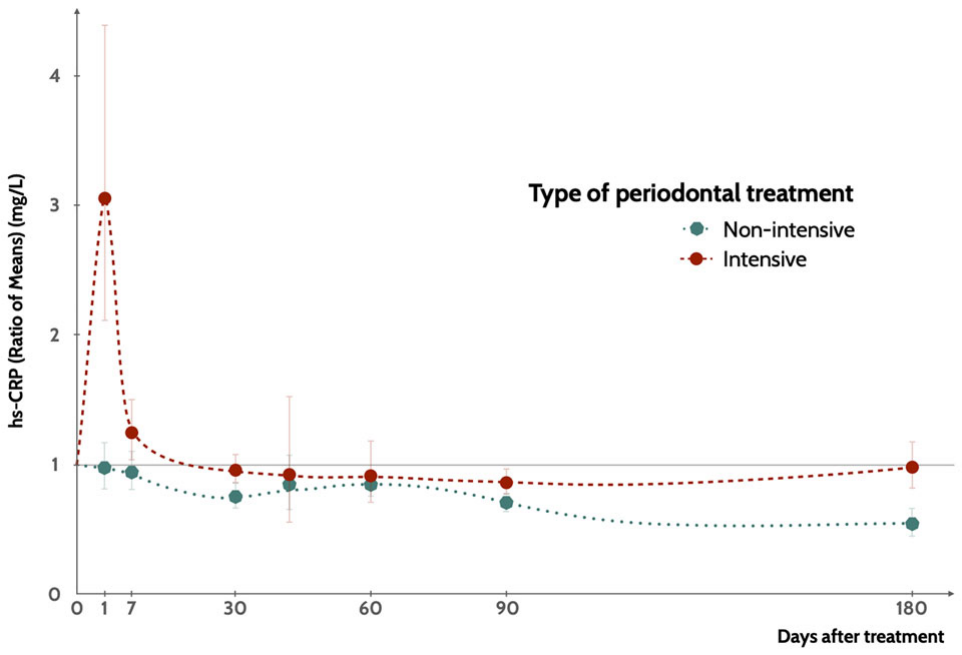
Variation of hs-CRP levels compared to baseline levels according to the type of NSPT, up to 180 days of follow-up. Points represent the mean value and the interval represents the 95%CI (**Appendices S29, S30**).

Non-intensive NSPT was associated with a progressive reduction of hs-CRP values up to 180 days after delivery (**Figure 2** and **Table 2**). Intensive NSPT resulted however in a sharp increase of hs-CRP one day after treatment, followed by a progressive reduction at 90 days after treatment but a new moderate increase at 180 days of follow-up (**Figure 2** and **Table 3**). Two studies reported data beyond 180 days (48, 49) with reductions of hs-CRP levels when compared those at baseline.

**Table 2:** Sensitivity analysis regarding the type of meta-analytical approach for non-intensive treatment studies.

Non-intensive treatment								
Day	n	ROM	95% CI	I^2^ (%)	n	SMD	95% CI	I^2^ (%)
1	4	0.98	0.82; 1.17	0.0	4	-0.05	-0.32; 0.20	0.0
7	6	0.95	0.81; 1.1	5.7	6	-0.08	-0.29; 0.19	5.7
30	12	0.75	0.67; 0.86	83.9	12	-0.53	-0.92; -0.14	83.0
42	3	0.84	0.66; 1.08	0.0	3	-0.20	-0.52; 0.12	0.0
60	13	0.84	0.76; 0.94	77.5	13	-0.48	-0.77; -0.19	77.5
90	23	0.71	0.64; 0.78	73.0	23	-0.71	-1.01; -0.41	82.9
180	11	0.55	0.45; 0.66	88.5	11	-1.25	-1.83; -0.67	92.4

CI, Confidence Interval; n, number of included articles; SMD, Standardized Mean Difference; ROM, Ratio of Mean.

**Table 3:** Sensitivity analysis regarding the type of meta-analytical approach for intensive treatment studies.

Intensive treatment								
Day	n	ROM	95% CI	I^2^ (%)	n	SMD	95% CI	I^2^ (%)
1	16	3.05	2.12; 4.38	95.5	16	1.86	1.25; 2.46	91.8
7	9	1.25	1.04; 1.5	73.8	9	0.39	0.08; 0.71	63.8
30	10	0.96	0.86; 1.07	4.9	10	-0.02	-0.21; 0.17	10.4
42	4	0.93	0.56; 1.52	34.7	4	-0.04	-0.32; 0.24	16.7
60	4	0.96	0.87; 1.05	0.0	4	-0.06	-0.28; 0.16	0.0
90	13	0.87	0.78; 0.97	82.8	13	-0.34	-0.61; 0.07	63.5
180	6	1.04	0.89; 1.21	64.7	6	0.02	-0.34; 0.38	35.9

CI, Confidence Interval; n, number of included articles; SMD, Standardized Mean Difference; ROM, Ratio of Mean.

## Discussion

This systematic review confirms a strong association between periodontitis and serum levels of CRP in otherwise systemically healthy individuals. Patients with periodontitis exhibited consistently higher values of CRP compared to healthy controls irrespective of the laboratory assessment adopted. Furthermore, different periodontal treatment modalities resulted in divergent kinetics of CRP over time with a rapid increase when intensive NSPT was performed but then a progressive reduction over time.

In contrast to previous studies (6, 10–12), this review expanded data on both observational and interventional studies liking periodontitis and systemic inflammation though CRP serum levels. So far, only one meta-analysis had shown high serum CRP levels in periodontitis patients had an average increase of 1.56 mg/L (p<0.001) compared to non-periodontitis cases (6). On the basis of our results, the more aggressive forms of periodontitis would be responsible for the greater increase in CRP levels in serum. This finding would be in agreement with the latest periodontitis case definitions when referring to cases of a rapid rate of progression and higher levels of CRP levels (50). These patients (previously known as suffering from aggressive periodontitis) often present a higher inflammatory load (50–52), nevertheless this should be researched more in depth in the future.

The first ever systematic review on this topic provided inconclusive non-significant results of NSPT on serum CRP levels (10). Two subsequent reviews confirmed periodontal treatment as effective in reducing serum CRP (11, 12). Differences between the different periodontal therapy approaches is unclear and the present review provides newer evidence on the changes of CRP serum levels following two different periodontal treatment modalities (intensive and non-intensive therapy). Furthermore, evidence pointed towards a better clinical response to treatment when intensive sessions of periodontal cleaning were performed (13–19). The latter however seemed to be linked to a greater systemic inflammatory response. In this review we describe the changes over time of systemic inflammation with a different pattern of changes in serum CRP even in otherwise healthy participants. Our intent was not to test whether periodontitis treatment would reduce systemic inflammation (as this has been extensively reviewed before and tested in appropriate RCTs) (6, 10–12) but rather to ascertain the directions of changes in the systemic inflammatory response linked to two treatment modalities over time. These differences bear clinical significance because NSPT, whilst is associated with a medium-term reduction of inflammation, it is also linked to acute spikes of hs-CRP which might have detrimental consequences when observed in patients who might suffer from other co-morbidities (such as, CVD or diabetes mellitus). Future studies are warranted to further expand data beyond 180 days after treatment and focus on patients reported outcomes.

Inflammation is a key biological process against injuries and infections and occurs at intervals throughout life, yet the persistence of a chronic systemic inflammatory state tracks future chronic conditions and associated complications (53). This risk prediction has been demonstrated when assessing basal levels of inflammation as assessed by CRP, particularly in the categorization of CVD risk categories (low risk <1 mg/L, medium between 1 and 3 mg/L and high >3 mg/L) (9). In the Justification for the Use of Statins in Prevention: an Intervention Trial Evaluating Rosuvastatin (JUPITER), lower rates of myocardial infarction, stroke and death were found following Rosuvastatin regimens in otherwise healthy adults with baseline CRP level ≥ 2mg/L and no hyperlipidemia (54). The reduction of 37% in CRP level observed in the test group stimulated further the discussion on the anti-inflammatory therapy as a valuable strategy in the prevention of CVD events. Despite CRP was excluded from the pathogenesis of inflammatory related conditions (i.e., such as atherosclerosis) by a subsequent mendelian randomization, it remains a proxy of the overall systemic inflammatory status. Additionally, the Canakinumab Antiinflammatory Thrombosis Outcome Study (CANTOS) trial showed the success of monoclonal antibody anti-inflammatory therapy in secondary CVD prevention confirming a benefit in terms of future CVD events in participants who maintained with CRP concentration less than 2mg/L throughout the trial (55). The increase in CRP concentration reported in patients with periodontitis and the reduction observed following periodontal therapy do not provide a definitive explanation on the pathways by which periodontitis could affect CVD risk but supports this hypothesis. Further, it remains to be demonstrated that the reduction of CRP levels after periodontal treatment will reduce the risk for CVD events. Nevertheless, periodontitis should be considered a common trigger of systemic inflammation.

Beyond the effect of the treatment of periodontitis in reducing CRP, the clinical relevance of the observed acute increase in inflammation after an intensive NSPT is still unknown (56), particularly in patients with other comorbidities (53). Non-intensive NSPT was recently defined as a safe procedure in patients on anti-thrombotic therapy, as it does not increase the risk for CVD events (56). Exploring the clinical relevance of the acute inflammation following intensive periodontal treatment and whether it poses a risk for patients with established chronic diseases should be a matter of further research.

**Figure 3:**
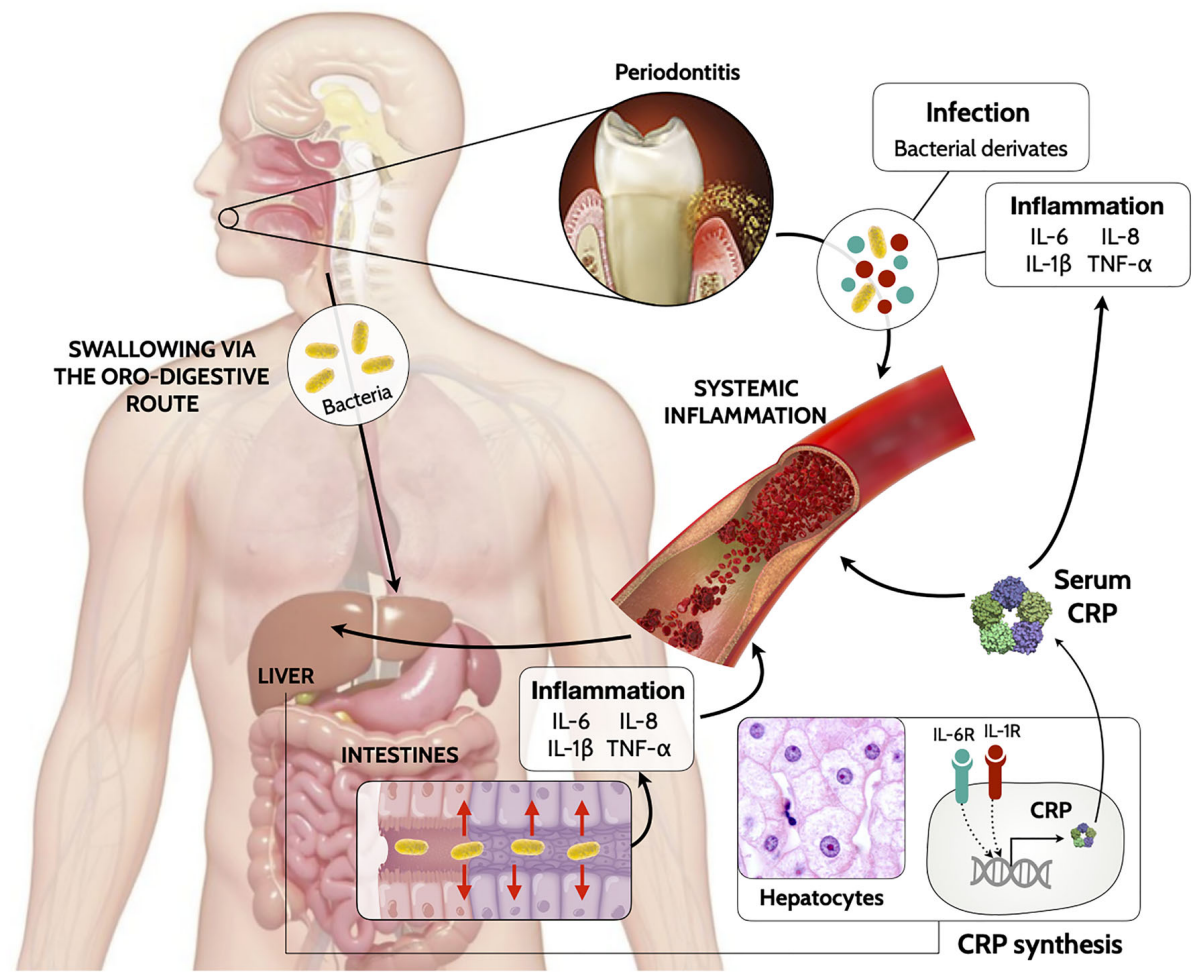
Biologically mechanism linking periodontitis to increase of systemic levels of CRP.

The exact mechanism of how periodontitis triggers CRP release is still under investigation. Gingival inflammation triggered by the dysbiotic dental biofilm is enriched with inflammatory mediators (i.e., interleukin [IL]-1β, IL-6 and tumor necrosis factor [TNF]-α) (2). Intra-oral hematogenous dissemination of periodontal bacteria occurs through the ulcerated epithelium or the overflow of inflammatory mediators from local periodontal damaged tissues to the systemic circulation triggering a systemic inflammatory response. Alternatively, periodontal bacteria can migrate through saliva swallowing *via* the oro-pharyngeal or oro-digestive sites, and ectopically colonize the gut causing an inflammatory reaction (3). The low-grade systemic inflammation driven by local inflammatory mediators promote local secretion of CRP (by adipocytes, vascular smooth muscle cells or gingival cells) (57) but also can generate a distant response by targeting hepatocytes (58, 59) after systemic dispersion (**Figure 3**). This state of prolonged inflammation ultimately results in alterations in the bone marrow (3) and with distinctive blood patterns (5). This is referred as trained myelopoiesis in the bone marrow, where hematopoietic stem cells undergo inflammation-adapted differentiation, leading to hyperreactive white cells (neutrophils and monocytes/macrophages) (3). As a result, when these hyperactive cells migrate through the bloodstream, they exacerbate inflammatory reactions in multiple locations and worsening on-going infectious and inflammatory diseases.

### Strengths and Potential Limitations

There are some limitations worth mentioning when reading this review. Estimates produced by our analyses included both representative and unrepresentative studies (as assessed through RoB assessment), which means that these results are not applicable to all patients with periodontitis. A number of variations in periodontal case definitions as well as CRP quantification methods used in the included studies, might have influenced the heterogeneity observed in the analyses. Further, methods to quantify CRP are becoming more precise and accurate, which may contribute to higher heterogeneity of estimates (60). Additionally, most studies did not include controls to follow and compare the hs-CRP changes over time with periodontitis. Also, although we were able to explore the possible role of smoking habits on baseline CRP levels, we could not further explore in interventional studies as this information was registered only at baseline and not updated throughout follow-ups. Similarly, we were unable to assess the impact BMI *via* meta-regressions because most studies lacked this information both at baseline and prospectively.

Our systematic review however was designed to follow a strict protocol, with a robust methodology and extensive search of all relevant evidence including all possible confounding factors. Further by using two meta-analytical approaches we are comfortable with the effect sizes across comparative groups and/or interventions (29) managing the high degree of heterogeneity (61).

In conclusion, periodontitis is associated inflammation as assessed by raised CRP levels and this is greater in those patients with more aggressive gingival inflammation. Further, periodontal treatment whether performed in an intensive or conventional fashion causes changes in CRP including a sharp increase over the first week and then a progressive reduction up to 6 months. These findings are relevant when discussing the potential implications of periodontitis on systemic health and especially in those individuals who suffer from other co-morbidities where systemic inflammation is recognized as a key driver not just of their onset but also disease-progression like diabetes and cardiovascular diseases.

## Data Availability Statement

The original contributions presented in the study are included in the article/**Supplementary Material**. Further inquiries can be directed to the corresponding author.

## Author Contributions

VM, JB, FD’A, conception and design of the work. VM, JB, SL, SH, data collection. VM, JB, PM, data analysis and interpretation. VM, JB, and CE, drafting the article. VM, JB, CE, SL, SH, PM, MO, JJM, FD’A, critical revision of the article and final approval of the version to be published. All authors contributed to the article and approved the submitted version.

## Funding

We would like to acknowledge that contribution of this work was undertaken at UCLH/UCL who received a proportion of funding from the Department of Health’s NIHR Biomedical Re-search Centre funding scheme. MO holds a NIHR Clinical Lectureship supported by the NIHR. Also, this work is financed by national funds through the FCT—Foundation for Science and Technology, I.P., under the project UIDB/04585/2020.

## Conflict of Interest

The authors declare that the research was conducted in the absence of any commercial or financial relationships that could be construed as a potential conflict of interest.

## Publisher’s Note

All claims expressed in this article are solely those of the authors and do not necessarily represent those of their affiliated organizations, or those of the publisher, the editors and the reviewers. Any product that may be evaluated in this article, or claim that may be made by its manufacturer, is not guaranteed or endorsed by the publisher.

## Abbreviations

CANTOS, Canakinumab Antiinflammatory Thrombosis Outcome Study; CENTRAL, The Cochrane Central Register of Controlled Trials; CI, Confidence Interval; CLIA, Chemiluminescent enzyme immunometric assay; CRP, C-Reactive Protein; CVD, cardiovascular disease; ELISA, Enzyme-Linked Immunosorbent Assay; hs-CRP, high-sensitivity CRP; IL, interleukin; JUPITER, Justification for the Use of Statins in Prevention: an Intervention Trial Evaluating Rosuvastatin; NSPT, Nonsurgical Periodontal Therapy; PRISMA, Preferred Reporting Items for Systematic Reviews and Meta-Analyses; RIA, Radial immunodiffusion assay; RoB, Risk of Bias; RoM, Ratio of Means; TNF, tumor necrosis factor.
