# Supplementary material for: Serum C-Reactive Protein and Periodontitis: A Systematic Review and Meta-Analysis
Source: Front Immunol. 2021 Jul 28;12:706432. doi: 10.3389/fimmu.2021.706432 (PMC8355591; doi:10.3389/fimmu.2021.706432)
Supplement: Supplementary file 1 [file DataSheet_1.docx]

**Online Supplemental Information**

Summary

Appendix S1. PRISMA 2009 Checklist 3

Appendix S2. List of potentially relevant studies not included in the systematic review, along with the reasons for exclusion. 5

Appendix S3 – References of included case control-studies 39

Appendix S4 – References of included intervention studies 45

Appendix S5. Studies characteristics comparing CRP and hs-CRP of otherwise healthy patient with PD, AgP and Non-PD diagnosis. 50

Appendix S6. Studies characteristics comparing levels of hs-CRP baseline and after periodontal treatment of otherwise healthy. 62

Appendix S7. Newcastle-Ottawa Scale In Non-randomized Studies – for case control studies 71

Appendix S8. Risk of Bias In Non-randomized Studies - of Interventions (ROBINS-I) tool 73

Appendix S9. RoB2 Tool 74

Appendix S10. Comparison between ROM and SMD results. 75

Appendix S11. Sensitivity analysis of type of studies using meta-regressions. 75

Appendix S12. Sensitivity analysis of the risk of bias. 76

Appendix S13. CRP of Healthy Periodontium versus Chronic Periodontitis individuals. Subgroup analysis according to the CRP method. 77

Appendix S14. CRP of Healthy Periodontium versus Chronic Periodontitis individuals. Subgroup analysis according to the study type. 78

Appendix S15. hs-CRP of Healthy Periodontium versus Chronic Periodontitis individuals. Subgroup analysis according to the CRP method. 79

Appendix S16. hs-CRP of Healthy Periodontium versus Chronic Periodontitis individuals. Subgroup analysis according to the type of study. 80

Appendix S17. CRP of Healthy Periodontium versus Aggressive Periodontitis individuals. 81

Appendix S18. hs-CRP of Healthy Periodontium versus Aggressive Periodontitis individuals. Subgroup analysis according to the type of study. 81

Appendix S19. CRP of Healthy Periodontium versus Periodontitis individuals. Subgroup analysis according to the CRP method. 82

Appendix S20. hs-CRP of Healthy Periodontium versus Periodontitis individuals. Subgroup analysis according to the study type. 83

Appendix S21. hs-CRP of Healthy Periodontium versus Periodontitis individuals. Subgroup analysis according to the hs-CRP method. 84

Appendix S22. CRP of Chronic Periodontitis versus Aggressive Periodontitis individuals. Subgroup analysis according to the CRP method. 85

Appendix S23. CRP of Chronic Periodontitis versus Aggressive Periodontitis individuals. Subgroup analysis according to the study type. 85

Appendix S24. hs-CRP of Chronic Periodontitis versus Aggressive Periodontitis individuals. 86

Appendix S25. Funnel plot and Egger’s test for CRP of Healthy Periodontium versus Chronic Periodontitis individuals. 86

Appendix S26. Funnel plot and Egger’s test for CRP of Healthy Periodontium versus Periodontitis individuals. 86

Appendix S27. Funnel plot and Egger’s test for hs-CRP of Healthy Periodontium versus Chronic Periodontitis individuals. 87

Appendix S28. Funnel plot and Egger’s test for hs-CRP of Healthy Periodontium versus Periodontitis individuals. 87

Appendix S29. Sensitivity analysis regarding the type of meta-analytical approach for non-intensive treatment studies 88

Appendix S30. Sensitivity analysis regarding the type of meta-analytical approach for intensive treatment studies 88

Appendix S31. Sensitivity analysis regarding the presence of studies without low risk of bias for non-intensive treatment studies 89

Appendix S32. Sensitivity analysis regarding the presence of studies without low risk of bias for intensive treatment studies 89

Appendix S33. Sensitivity analysis for the influence of the presence of smoking participants using meta-regression in non-intensive and intensive treatment studies 90

Supplementary Table 1. PRISMA 2009 Checklist

| **Section/topic** | **#** | **Checklist item** | **Reported on page #** |
| --- | --- | --- | --- |
| **TITLE** | | |  |
| Title | 1 | Identify the report as a systematic review, meta-analysis, or both. | 1 |
| **ABSTRACT** | | |  |
| Structured summary | 2 | Provide a structured summary including, as applicable: background; objectives; data sources; study eligibility criteria, participants, and interventions; study appraisal and synthesis methods; results; limitations; conclusions and implications of key findings; systematic review registration number. | 2 |
| **INTRODUCTION** | | |  |
| Rationale | 3 | Describe the rationale for the review in the context of what is already known. | 3 |
| Objectives | 4 | Provide an explicit statement of questions being addressed with reference to participants, interventions, comparisons, outcomes, and study design (PICOS). | 3 |
| **METHODS** | | |  |
| Protocol and registration | 5 | Indicate if a review protocol exists, if and where it can be accessed (e.g., Web address), and, if available, provide registration information including registration number. | 3-4 |
| Eligibility criteria | 6 | Specify study characteristics (e.g., PICOS, length of follow-up) and report characteristics (e.g., years considered, language, publication status) used as criteria for eligibility, giving rationale. | 4 |
| Information sources | 7 | Describe all information sources (e.g., databases with dates of coverage, contact with study authors to identify additional studies) in the search and date last searched. | 4 |
| Search | 8 | Present full electronic search strategy for at least one database, including any limits used, such that it could be repeated. | 4 |
| Study selection | 9 | State the process for selecting studies (i.e., screening, eligibility, included in systematic review, and, if applicable, included in the meta-analysis). | 4-5 |
| Data collection process | 10 | Describe method of data extraction from reports (e.g., piloted forms, independently, in duplicate) and any processes for obtaining and confirming data from investigators. | 5 |
| Data items | 11 | List and define all variables for which data were sought (e.g., PICOS, funding sources) and any assumptions and simplifications made. | 5 |
| Risk of bias in individual studies | 12 | Describe methods used for assessing risk of bias of individual studies (including specification of whether this was done at the study or outcome level), and how this information is to be used in any data synthesis. | 5 |
| Summary measures | 13 | State the principal summary measures (e.g., risk ratio, difference in means). | 5-6 |
| Synthesis of results | 14 | Describe the methods of handling data and combining results of studies, if done, including measures of consistency (e.g., I^2^) for each meta-analysis. | 5-6 |

| **Section/topic** | **#** | **Checklist item** | **Reported on page #** |
| --- | --- | --- | --- |
| Risk of bias across studies | 15 | Specify any assessment of risk of bias that may affect the cumulative evidence (e.g., publication bias, selective reporting within studies). | NA |
| Additional analyses | 16 | Describe methods of additional analyses (e.g., sensitivity or subgroup analyses, meta-regression), if done, indicating which were pre-specified. | 6 |
| **RESULTS** | | |  |
| Study selection | 17 | Give numbers of studies screened, assessed for eligibility, and included in the review, with reasons for exclusions at each stage, ideally with a flow diagram. | 6 |
| Study characteristics | 18 | For each study, present characteristics for which data were extracted (e.g., study size, PICOS, follow-up period) and provide the citations. | 6 |
| Risk of bias within studies | 19 | Present data on risk of bias of each study and, if available, any outcome level assessment (see item 12). | 6 |
| Results of individual studies | 20 | For all outcomes considered (benefits or harms), present, for each study: (a) simple summary data for each intervention group (b) effect estimates and confidence intervals, ideally with a forest plot. | 6 |
| Synthesis of results | 21 | Present results of each meta-analysis done, including confidence intervals and measures of consistency. | 6-7 |
| Risk of bias across studies | 22 | Present results of any assessment of risk of bias across studies (see Item 15). | NA |
| Additional analysis | 23 | Give results of additional analyses, if done (e.g., sensitivity or subgroup analyses, meta-regression [see Item 16]). | 7 |
| **DISCUSSION** | | |  |
| Summary of evidence | 24 | Summarize the main findings including the strength of evidence for each main outcome; consider their relevance to key groups (e.g., healthcare providers, users, and policy makers). | 7 |
| Limitations | 25 | Discuss limitations at study and outcome level (e.g., risk of bias), and at review-level (e.g., incomplete retrieval of identified research, reporting bias). | 7-9 |
| Conclusions | 26 | Provide a general interpretation of the results in the context of other evidence, and implications for future research. | 9 |
| **FUNDING** | | |  |
| Funding | 27 | Describe sources of funding for the systematic review and other support (e.g., supply of data); role of funders for the systematic review. | 9 |

NA – Not applicable

*From:*  Moher D, Liberati A, Tetzlaff J, Altman DG, The PRISMA Group (2009). Preferred Reporting Items for Systematic Reviews and Meta-Analyses: The PRISMA Statement. PLoS Med 6(7): e1000097. doi:10.1371/journal.pmed1000097

For more information, visit: **www.prisma-statement.org**.

Supplementary Table 2. List of potentially relevant studies not included in the systematic review, along with the reasons for exclusion.

| **Year** | **N** | **Reference** | **Reason for exclusion** |
| --- | --- | --- | --- |
| 2021 | 1 | Davison E, Johnston W, Piela K, Rosier BT, Paterson M, Mira A, Culshaw S. The Subgingival Plaque Microbiome, Systemic Antibodies Against Bacteria and Citrullinated Proteins Following Periodontal Therapy. Pathogens. 2021 Feb 10;10(2):193. doi: 10.3390/pathogens10020193. PMID: 33578802. | No control group |
| 2021 | 2 | Ladegaard Grønkjær L, Holmstrup P, Jepsen P, Vilstrup H. The impact of oral diseases in cirrhosis on complications and mortality. JGH Open. 2021 Jan 12;5(2):294-300. doi: 10.1002/jgh3.12489. PMID: 33553670; PMCID: PMC7857277. | Patients with systemic diseases |
| 2021 | 3 | Inanc N, Mumcu G, Can M, Yay M, Silbereisen A, Manoil D, Direskeneli H, Bostanci N. Elevated serum TREM-1 is associated with periodontitis and disease activity in rheumatoid arthritis. Sci Rep. 2021 Feb 3;11(1):2888. doi: 10.1038/s41598-021-82335-9. PMID: 33536478; PMCID: PMC7859204. | Patients with systemic diseases |
| 2021 | 4 | Leira Y, Domínguez C, Ameijeira P, López-Arias E, Ávila-Gómez P, Pérez-Mato M, Sobrino T, Campos F, Blanco J, Leira R. Mild systemic inflammation enhances response to OnabotulinumtoxinA in chronic migraineurs. Sci Rep. 2021 Jan 13;11(1):1092. doi: 10.1038/s41598-020-80283-4. PMID: 33441852; PMCID: PMC7806961. | Patients with systemic diseases |
| 2021 | 5 | Chang HY, Kim AR, Pi SH, You HK. A Study on the Correlation between C-Reactive Protein Concentration and Teeth with a ≥5 mm Periodontal Pocket in Chronic Periodontitis Patients. Int J Dent. 2020 Dec 23;2020:8832186. doi: 10.1155/2020/8832186. PMID: 33424972; PMCID: PMC7773474. | No control group |
| 2021 | 6 | Almoznino G, Zini A, Kedem R, Protter NE, Zur D, Abramovitz I. Hypertension and Its Associations with Dental Status: Data from the Dental, Oral, Medical Epidemiological (DOME) Nationwide Records-Based Study. J Clin Med. 2021 Jan 6;10(2):176. doi: 10.3390/jcm10020176. PMID: 33419028; PMCID: PMC7825310. | Patients with systemic diseases |
| 2021 | 7 | Wojtkowska A, Zapolski T, Wysokińska-Miszczuk J, Wysokiński AP. The inflammation link between periodontal disease and coronary atherosclerosis in patients with acute coronary syndromes: case-control study. BMC Oral Health. 2021 Jan 6;21(1):5. doi: 10.1186/s12903-020-01356-4. PMID: 33407375; PMCID: PMC7789370. | No control group |
| 2021 | 8 | Chila-Moreno L, Rodríguez LS, Bautista-Molano W, Bello-Gualtero JM, Ramos-Casallas A, Romero-Sánchez C. Anti-carbamylated protein and peptide antibodies as potential inflammatory joint biomarkers in the relatives of rheumatoid arthritis patients. Int J Rheum Dis. 2020 Dec;23(12):1698-1706. doi: 10.1111/1756-185X.13977. Epub 2020 Nov 4. PMID: 33146469. | No control group |
| 2021 | 9 | Montero E, López M, Vidal H, Martínez M, Virto L, Marrero J, Herrera D, Zapatero A, Sanz M. Impact of periodontal therapy on systemic markers of inflammation in patients with metabolic syndrome: A randomized clinical trial. Diabetes Obes Metab. 2020 Nov;22(11):2120-2132. doi: 10.1111/dom.14131. Epub 2020 Aug 20. PMID: 32613714. | Patients with systemic diseases |
| 2021 | 10 | Kotronia E, Wannamethee SG, Papacosta AO, Whincup PH, Lennon LT, Visser M, Kapila YL, Weyant RJ, Ramsay SE. Poor Oral Health and Inflammatory, Hemostatic, and Cardiac Biomarkers in Older Age: Results From Two Studies in the UK and USA. J Gerontol A Biol Sci Med Sci. 2021 Jan 18;76(2):346-351. doi: 10.1093/gerona/glaa096. PMID: 32306041; PMCID: PMC7812424. | Patients with systemic diseases |
| 2020 | 11 | Zhang W, Wang W, Chu C, Jing J, Yao NA, Sun Q, Li S. Clinical, inflammatory and microbiological outcomes of full-mouth scaling with adjunctive glycine powder air-polishing: A randomized trial. J Clin Periodontol. 2020 Nov 10. doi: 10.1111/jcpe.13400. Epub ahead of print. PMID: 33174234. | No mean or/and standard deviation values |
| 2020 | 12 | Costa FO, Esteves Lima RP, Cortelli SC, Costa AM, Cortelli JR, Cota LOM. Effect of compliance during periodontal maintenance therapy on c-reactive protein levels: a 6-year follow-up. J Clin Periodontol. 2020 Dec 1. doi: 10.1111/jcpe.13407. Epub ahead of print. PMID: 33259118. | Undefined follow-up period |
| 2020 | 13 | Aoyama N, Fujii T, Kida S, Nozawa I, Taniguchi K, Fujiwara M, Iwane T, Tamaki K, Minabe M. Association of Periodontal Status, Number of Teeth, and Obesity: A Cross-Sectional Study in Japan. J Clin Med. 2021 Jan 8;10(2):208. doi: 10.3390/jcm10020208. PMID: 33435628; PMCID: PMC7827095. | Patients with systemic diseases |
| 2020 | 14 | Pappe CL, Steckhan N, Hoedke D, Jepsen S, Rauch G, Keller T, Michalsen A, Dommisch H. Prolonged multimodal fasting modulates periodontal inflammation in female patients with metabolic syndrome: A prospective cohort study. J Clin Periodontol. 2021 Jan 4. doi: 10.1111/jcpe.13419. Epub ahead of print. PMID: 33393121. | Patients with systemic diseases |
| 2020 | 15 | Mizutani K, Mikami R, Gohda T, Gotoh H, Aoyama N, Matsuura T, Kido D, Takeda K, Izumi Y, Sasaki Y, Iwata T. Poor oral hygiene and dental caries predict high mortality rate in hemodialysis: a 3-year cohort study. Sci Rep. 2020 Dec 14;10(1):21872. doi: 10.1038/s41598-020-78724-1. PMID: 33318507; PMCID: PMC7736314. | Patients with systemic diseases |
| 2020 | 16 | Dent SC, Berger SM, Griffin JS. Biocultural pathways linking periodontal disease expression to food insecurity, immune dysregulation, and nutrition. Am J Hum Biol. 2020 Dec 10:e23549. doi: 10.1002/ajhb.23549. Epub ahead of print. PMID: 33300640. | Data previously published |
| 2020 | 17 | Mahapatra A, Nayak R, Satpathy A, Pati BK, Mohanty R, Mohanty G, Beura R. Maternal periodontal status, oral inflammatory load, and systemic inflammation are associated with low infant birth weight. J Periodontol. 2020 Nov 5. doi: 10.1002/JPER.20-0266. Epub ahead of print. PMID: 33155287. | Patients with systemic diseases |
| 2020 | 18 | Mikami R, Mizutani K, Gohda T, Gotoh H, Matsuyama Y, Aoyama N, Matsuura T, Kido D, Takeda K, Izumi Y, Fujiwara T, Iwata T. Association between circulating tumor necrosis factor receptors and oral bacterium in patients receiving hemodialysis: a cross-sectional study. Clin Exp Nephrol. 2021 Jan;25(1):58-65. doi: 10.1007/s10157-020-01952-2. Epub 2020 Aug 20. PMID: 32816134. | Patients with systemic diseases |
| 2020 | 19 | Białowąs K, Radwan-Oczko M, Duś-Ilnicka I, Korman L, Świerkot J. Periodontal disease and influence of periodontal treatment on disease activity in patients with rheumatoid arthritis and spondyloarthritis. Rheumatol Int. 2020 Mar;40(3):455-463. | Patients with systemic diseases |
| 2020 | 20 | Boyapati R, Vudathaneni V, Nadella SB, Ramachandran R, Dhulipalla R, Adurty C. Mapping the link between cardiac biomarkers and chronic periodontitis: A clinico-biochemical study. J Indian Soc Periodontol. 2020 Jul-Aug;24(4):309-315. | Patients with systemic diseases |
| 2020 | 21 | Cheah CW, Al-Maleki AR, Vadivelu J, Danaee M, Sockalingam S, Baharuddin NA, Vaithilingam RD. Salivary and serum cathelicidin LL-37 levels in subjects with rheumatoid arthritis and chronic periodontitis. Int J Rheum Dis. 2020 Aug 2. | Patients with systemic diseases |
| 2020 | 22 | Disale PR, Zope SA, Suragimath G, Varma AS, Pisal A. Prevalence and severity of periodontitis in patients with established rheumatoid arthritis and osteoarthritis. J Family Med Prim Care. 2020 Jun 30;9(6):2919-2925. | Patients with systemic diseases |
| 2020 | 23 | Esteves-Lima RP, Reis CS, Santirocchi-Júnior F, Abreu LG, Costa FO. Association between periodontitis and serum c-reactive protein levels. J Clin Exp Dent. 2020 Sep 1;12(9):e838-e843. doi: 10.4317/jced.57041. PMID: 32994872; PMCID: PMC7511054. | Data in thresholds |
| 2020 | 24 | Gupta S, Suri P, Patil PB, Rajguru JP, Gupta P, Patel N. Comparative evaluation of role of hs C -reactive protein as a diagnostic marker in chronic periodontitis patients. J Family Med Prim Care. 2020 Mar 26;9(3):1340-1347. | Unsuitable periodontal case definition |
| 2020 | 25 | Hatta K, Gondo Y, Kamide K, Masui Y, Inagaki H, Nakagawa T, Matsuda KI, Inomata C, Takeshita H, Mihara Y, Fukutake M, Kitamura M, Murakami S, Kabayama M, Ishizaki T, Arai Y, Sugimoto K, Rakugi H, Maeda Y, Ikebe K. Occlusal force predicted cognitive decline among 70- and 80-year-old Japanese: A 3-year prospective cohort study. J Prosthodont Res. 2020 Apr;64(2):175-181. | No mean or/and standard deviation values |
| 2020 | 26 | Keceli HG, Ercan N, Karsiyaka Hendek M, Kisa U, Mesut B, Olgun E. The effect of the systemic folic acid intake as an adjunct to scaling and root planing on clinical parameters and homocysteine and C-reactive protein levels in gingival crevicular fluid of periodontitis patients: A randomized placebo-controlled clinical trial. J Clin Periodontol. 2020 May;47(5):602-613. | No serum CRP values |
| 2020 | 27 | Li P, He L, Chen ZB, Luan QX. Biomarkers in Metabolic Syndrome Patients with Chronic Periodontitis. Chin J Dent Res. 2020;23(3):191-197. | Patients with systemic diseases |
| 2020 | 28 | Matern J, Koch R, Petersmann A, Kocher T, Eickholz P, Lorenz K, Kim TS, Meyle J, Kaner D, Schlagenhauf U, Gravemeier M, Harks I, Ehmke B. Effect of periodontal therapy on adipokine biomarkers in overweight. J Clin Periodontol. 2020 Jul;47(7):842-850 | Unsuitable periodontal treatment |
| 2020 | 29 | Meisel P, Pink C, Pitchika V, Nauck M, Völzke H, Kocher T. Competing interplay between systemic and periodontal inflammation: obesity overrides the impact of oral periphery. Clin Oral Investig. 2020 Aug 22. | Data in thresholds |
| 2020 | 30 | Mikami R, Mizutani K, Gohda T, Gotoh H, Matsuyama Y, Aoyama N, Matsuura T, Kido D, Takeda K, Izumi Y, Fujiwara T, Iwata T. Association between circulating tumor necrosis factor receptors and oral bacterium in patients receiving hemodialysis: a cross-sectional study. Clin Exp Nephrol. 2020 Aug 20. | Patients with systemic diseases |
| 2020 | 31 | Montero E, López M, Vidal H, Martínez M, Virto L, Marrero J, Herrera D, Zapatero A, Sanz M. Impact of periodontal therapy on systemic markers of inflammation in patients with metabolic syndrome: A randomized clinical trial. Diabetes Obes Metab. 2020 Jul 1. | Patients with systemic diseases |
| 2020 | 32 | Munday MR, Rodricks R, Fitzpatrick M, Flood VM, Gunton JE. A Pilot Study Examining Vitamin C Levels in Periodontal Patients. Nutrients. 2020 Jul 28;12(8):2255. | No control group |
| 2020 | 33 | Muñoz Aguilera E, Leira Y, Miró Catalina Q, Orlandi M, Czesnikiewicz-Guzik M, Guzik TJ, Hingorani AD, Nart J, D'Aiuto F. Is Systemic Inflammation a Missing Link Between Periodontitis and Hypertension? Results from Two Large Populations-based Surveys. J Intern Med. 2020 Sep 23. | No control group |
| 2020 | 34 | Nguyen VB, Nguyen TT, Huynh NC, Le TA, Hoang HT. Relationship between periodontitis and rheumatoid arthritis in Vietnamese patients. Acta Odontol Scand. 2020 Oct;78(7):522-528. | Patients with systemic diseases |
| 2020 | 35 | Rocha LO, Rocha E, Succi GM, Brito Junior RB. Association between Periodontitis, Genetic Polymorphisms and Presence of Coronary Artery Disease in Southern Brazil. Arq Bras Cardiol. 2020 Feb;114(2):268-272. | Data in thresholds |
| 2020 | 36 | Schöffer C, Oliveira LM, Santi SS, Antoniazzi RP, Zanatta FB. C-reactive protein levels are associated with periodontitis and periodontal inflamed surface area in adults with end-stage renal disease. J Periodontol. 2020 Oct 11. doi: 10.1002/JPER.20-0200. Epub ahead of print. PMID: 33040368. | No control group |
| 2020 | 37 | Sparrow TV, Dodington DW, Yumol JL, Fritz PC, Ward WE. Higher intakes of flavonoids are associated with lower salivary IL-1β and maintenance of periodontal health 3-4 years after scaling and root planing. J Clin Periodontol. 2020 Apr;47(4):461-469. | No mean or/and standard deviation values |
| 2020 | 38 | Stănescu I, Bulboacă AE, Micu IC, Bolboacă SD, Feștilă DG, Bulboacă AC, Bodizs G, Dogaru G, Boarescu PM, Popa-Wagner A, Roman A. Gender Differences in the Levels of Periodontal Destruction, Behavioral Risk Factors and Systemic Oxidative Stress in Ischemic Stroke Patients: A Cohort Pilot Study. J Clin Med. 2020 Jun 4;9(6):1744. | Patients with systemic diseases |
| 2020 | 39 | Teixeira FCF, Marin-Leon L, Gomes EP, PedrÃo AMN, Pereira ADC, Francisco PMSB. Relationship between periodontitis and subclinical risk indicators for chronic non-communicable diseases. Braz Oral Res. 2020 Jun 19;34:e058. | No control group |
| 2020 | 40 | Tuominen H, Taina M, Puranen M, Onatsu J, Huumonen S, Vanninen R. Serum High-Sensitive C-reactive Protein May Reflect Periodontitis in Patients With Stroke. In Vivo. 2020 Sep-Oct;34(5):2829-2835. | Data in thresholds |
| 2020 | 41 | Wang Y, Liu HN, Zhen Z, Pelekos G, Wu MZ, Chen Y, Tonetti M, Tse HF, Yiu KH, Jin L. A randomized controlled trial of the effects of non-surgical periodontal therapy on cardiac function assessed by echocardiography in type 2 diabetic patients. J Clin Periodontol. 2020 Jun;47(6):726-736. | Patients with systemic diseases |
| 2020 | 42 | Zhang Y, Leveille SG, Edward J. Wisdom teeth, periodontal disease, and C-reactive protein in US adults. Public Health. 2020 Sep 14;187:97-102. | Incomplete CRP report levels |
| 2019 | 43 | Anusha D, Chaly PE, Junaid M, Nijesh JE, Shivashankar K, Sivasamy S. Efficacy of a mouthwash containing essential oils and curcumin as an adjunct to nonsurgical periodontal therapy among rheumatoid arthritis patients with chronic periodontitis: A randomized controlled trial. Indian J Dent Res. 2019 Jul-Aug;30(4):506-511. doi: 10.4103/ijdr.IJDR_662_17. PMID: 31745043. | Patients with systemic diseases |
| 2019 | 44 | Ameijeira P, Leira Y, Domínguez C, Leira R, Blanco J. Association between periodontitis and chronic migraine: a case-control study. Odontology. 2019 Jan;107(1):90-95. | Patients with systemic diseases |
| 2019 | 45 | Pedroso JF, Lotfollahi Z, Albattarni G, Arrruda Schulz M, Monteiro A, Sehnem AL, Gidlund MA, Figueiredo Neto AM, Jardini MAN. Influence of Periodontal Disease on cardiovascular markers in Diabetes Mellitus patients. Sci Rep. 2019 Nov 6;9(1):16138. | Patients with systemic diseases |
| 2019 | 46 | Rapone B, Converti I, Santacroce L, Cesarano F, Vecchiet F, Cacchio L, Scacco S, Grassi R, Grassi FR, Gnoni A, Nardi GM. Impact of Periodontal Inflammation on Nutrition and Inflammation Markers in Hemodialysis Patients. Antibiotics (Basel). 2019 Nov 1;8(4):209. | Patients with systemic diseases |
| 2019 | 47 | Kaushal S, Singh AK, Lal N, Das SK, Mahdi AA. Effect of periodontal therapy on disease activity in patients of rheumatoid arthritis with chronic periodontitis. J Oral Biol Craniofac Res. 2019 Apr-Jun;9(2):128-132. doi: 10.1016/j.jobcr.2019.02.002. Epub 2019 Feb 2. | Patients with systemic diseases |
| 2019 | 48 | Purnamasari D, Khumaedi AI, Soeroso Y, Marhamah S. The influence of diabetes and or periodontitis on inflammation and adiponectin level. Diabetes Metab Syndr. 2019 May-Jun;13(3):2176-2182. doi: 10.1016/j.dsx.2019.05.012. Epub 2019 May 22. PMID: 31235154. | Patients with systemic diseases |
| 2019 | 49 | Rinaudo-Gaujous M, Blasco-Baque V, Miossec P, Gaudin P, Farge P, Roblin X, Thomas T, Paul S, Marotte H. Infliximab Induced a Dissociated Response of Severe Periodontal Biomarkers in Rheumatoid Arthritis Patients. J Clin Med. 2019 May 26;8(5):751. doi: 10.3390/jcm8050751. PMID: 31130713; PMCID: PMC6571563. | Patients with systemic diseases |
| 2019 | 50 | Rodríguez-Lozano B, González-Febles J, Garnier-Rodríguez JL, Dadlani S, Bustabad-Reyes S, Sanz M, Sánchez-Alonso F, Sánchez-Piedra C, González-Dávila E, Díaz-González F. Association between severity of periodontitis and clinical activity in rheumatoid arthritis patients: a case-control study. Arthritis Res Ther. 2019 Jan 18;21(1):27. doi: 10.1186/s13075-019-1808-z. PMID: 30658685; PMCID: PMC6339403. | Patients with systemic diseases |
| 2019 | 51 | Tariq (2019). Comparative Research to Assess the Level of C-Reactive Protein (CRP) among Smokers and Non-smokers diagnosed with Chronic Periodontitis. | No control group |
| 2019 | 52 | Tasdemir, Z., Oskaybas, M. N., Alkan, B. A., & Cakmak, O. (2019). The Effects of Ozone Therapy on Periodontal Therapy: A Randomized Placebo-Controlled Clinical Trial. Oral Diseases. doi:10.1111/odi.13060 | No control group |
| 2019 | 53 | Torrungruang K, Katudat D, Mahanonda R, Sritara P, Udomsak A. Periodontitis is associated with elevated serum levels of cardiac biomarkers-Soluble ST2 and C-reactive protein. J Clin Periodontol. 2019 Aug;46(8):809-818. | Unsuitable control group |
| 2019 | 54 | Yeter HH, Erten Y, Isler SC, Soysal F, Elbeg S, Unsal B. Could drug burden be associated with severe periodontitis in patients receiving haemodialysis? J Ren Care. 2019 Dec;45(4):239-247. doi: 10.1111/jorc.12299. Epub 2019 Sep 28. PMID: 31564070. | Patients with systemic diseases |
| 2019 | 55 | Zare Javid A, Bazyar H, Gholinezhad H, Rahimlou M, Rashidi H, Salehi P, Haghighi-Zadeh MH. The effects of ginger supplementation on inflammatory, antioxidant, and periodontal parameters in type 2 diabetes mellitus patients with chronic periodontitis under non-surgical periodontal therapy. A double-blind, placebo-controlled trial. Diabetes Metab Syndr Obes. 2019 Sep 6;12:1751-1761. doi: 10.2147/DMSO.S214333. PMID: 32021341; PMCID: PMC6737165. | Patients with systemic diseases |
| 2019 | 56 | Montenegro MM, Ribeiro IWJ, Kampits C, Saffi MAL, Furtado MV, Polanczyk CA, Haas AN, Rösing CK. Randomized controlled trial of the effect of periodontal treatment on cardiovascular risk biomarkers in patients with stable coronary artery disease: Preliminary findings of 3 months. J Clin Periodontol. 2019 Mar;46(3):321-331. doi: 10.1111/jcpe.13085. Epub 2019 Mar 6. PMID: 30761568. | Patients with systemic diseases |
| 2019 | 57 | Dengizek Eltas S, Gursel M, Eltas A, Alptekin NO, Ataoglu T. Evaluation of long-term effects of diode laser application in periodontal treatment of poorly controlled type 2 diabetic patients with chronic periodontitis. Int J Dent Hyg. 2019 Nov;17(4):292-299. doi: 10.1111/idh.12384. Epub 2019 Feb 19. PMID: 30697968. | Patients with systemic diseases |
| 2019 | 58 | Cosgarea R, Tristiu R, Dumitru RB, Arweiler NB, Rednic S, Sirbu CI, Lascu L, Sculean A, Eick S. Effects of non-surgical periodontal therapy on periodontal laboratory and clinical data as well as on disease activity in patients with rheumatoid arthritis. Clin Oral Investig. 2019 Jan;23(1):141-151. doi: 10.1007/s00784-018-2420-3. Epub 2018 Mar 27. PMID: 29589156. | Patients with systemic diseases |
| 2018 | 59 | Boyapati R, Chinthalapani S, Ramisetti A, Salavadhi SS, Ramachandran R. Association of pentraxin and high-sensitive C-reactive protein as inflammatory biomarkers in patients with chronic periodontitis and peripheral arterial disease. J Indian Soc Periodontol. 2018 Mar-Apr;22(2):112-115. doi: 10.4103/jisp.jisp_290_17. PMID: 29769764; PMCID: PMC5939017. | Patients with systemic diseases |
| 2018 | 60 | Chen L, Wei B, Xu L, Wu Y. The association of inflammatory markers and periodontal indexes with the risk of coronary heart disease in Chinese patients with type 2 diabetes mellitus. Diabetes Res Clin Pract. 2018 Jan;135:37-44. doi: 10.1016/j.diabres.2017.10.008. Epub 2017 Oct 28. PMID: 29111278. | Patients with systemic diseases |
| 2018 | 61 | Cholewa M, Madziarska K, Radwan-Oczko M. The association between periodontal conditions, inflammation, nutritional status and calcium-phosphate metabolism disorders in hemodialysis patients. J Appl Oral Sci. 2018 Jul 23;26:e20170495. | Unsuitable control group |
| 2018 | 62 | Ahmad A, Nazar Z, Swaminathan D. C-Reactive Protein Levels and Periodontal Diseases During Pregnancy in Malaysian Women. Oral Health Prev Dent. 2018;16(3):281-289. | Patients with systemic diseases |
| 2018 | 63 | Escobar GF, Abdalla DR, Beghini M, Gotti VB, Rodrigues Junior V, Napimoga MH, Ribeiro BM, Rodrigues DBR, Nogueira RD, Pereira SAL (2018). Levels of Pro and Anti-inflammatory Citokynes and C-Reactive Protein in Patients with Chronic Periodontitis Submitted to Nonsurgical Periodontal Treatment. Asian Pac J Cancer Prev, Jul 27;19(7):1927-1933. | No mean or/and standard deviation values |
| 2018 | 64 | Martín A, Bravo M, Arrabal M, Magán-Fernández A, Mesa F. Chronic periodontitis is associated with erectile dysfunction. A case-control study in european population. J Clin Periodontol. 2018 Jul;45(7):791-798. doi: 10.1111/jcpe.12909. Epub 2018 May 28. PMID: 29723438. | Patients with systemic diseases |
| 2018 | 65 | Górski B, Górska R. The impact of periodontal treatment on inflammatory markers and cellular parameters associated with atherosclerosis in patients after myocardial infarction. Cent Eur J Immunol. 2018;43(4):442-452. | Patients with systemic diseases |
| 2018 | 66 | Kang, J.-H., Lee, Y.-H., & Kho, H.-S. (2018). Clinical factors affecting salivary transferrin level, a marker of blood contamination in salivary analysis. BMC Oral Health, 18(1). doi:10.1186/s12903-018-0510-x | No case group |
| 2018 | 67 | Kawano T, Shigeishi H, Fukada E, Yanagisawa T, Kuroda N, Takemoto T, Sugiyama M. Changes in bacterial number at different sites of oral cavity during perioperative oral care management in gastrointestinal cancer patients: preliminary study. J Appl Oral Sci. 2018 Jun 18;26:e20170516. doi: 10.1590/1678-7757-2017-0516. PMID: 29898181; PMCID: PMC6010331. | Patients with systemic diseases |
| 2018 | 68 | Kobayashi T, Kido JI, Ishihara Y, Omori K, Ito S, Matsuura T, Bando T, Wada J, Murasawa A, Nakazono K, Mitani A, Takashiba S, Nagata T, Yoshie H. The KCNQ1 gene polymorphism as a shared genetic risk for rheumatoid arthritis and chronic periodontitis in Japanese adults: A pilot case-control study. J Periodontol. 2018 Mar;89(3):315-324. doi: 10.1002/JPER.17-0412. Epub 2018 Mar 12. PMID: 29520783. | Patients with systemic diseases |
| 2018 | 69 | Kure K, Sato H, Aoyama N, Izumi Y. Accelerated inflammation in peripheral artery disease patients with periodontitis. J Periodontal Implant Sci. 2018 Dec 27;48(6):337-346. doi: 10.5051/jpis.2018.48.6.337. PMID: 30619635; PMCID: PMC6312877. | Patients with systemic diseases |
| 2018 | 70 | Li Z, Lu C, Qiu J, Liu S, Liu X, Ma S, Lai R. Correlation of serum adipocytokine levels with glycolipid metabolism and inflammatory factors in obese patients with periodontal disease. Int J Clin Exp Pathol. 2018 Mar 1;11(3):1620-1628. PMID: 31938261; PMCID: PMC6958146. | Patients with systemic diseases |
| 2018 | 71 | Tabeta, K., Hosojima, M., Nakajima, M., Miyauchi, S., Miyazawa, H., Takahashi, N., … Yoshie, H. (2018). Increased serum PCSK9, a potential biomarker to screen for periodontitis, and decreased total bilirubin associated with probing depth in a Japanese community survey. Journal of Periodontal Research, 53(3), 446–456. doi:10.1111/jre.12533 | Only males included |
| 2018 | 72 | Torrungruang K, Ongphiphadhanakul B, Jitpakdeebordin S, Sarujikumjornwatana S. Mediation analysis of systemic inflammation on the association between periodontitis and glycaemic status. J Clin Periodontol. 2018 May;45(5):548-556. doi: 10.1111/jcpe.12884. Epub 2018 Apr 10. PMID: 29500831. | Patients with systemic diseases |
| 2018 | 73 | Zhao X, Liu Z, Shu D, Xiong Y, He M, Xu S, Si S, Guo B. Association of Periodontitis with Rheumatoid Arthritis and the Effect of Non-Surgical Periodontal Treatment on Disease Activity in Patients with Rheumatoid Arthritis. Med Sci Monit. 2018 Aug 20;24:5802-5810. | Patients with systemic diseases |
| 2018 | 74 | Zambon M, Mandò C, Lissoni A, Anelli GM, Novielli C, Cardellicchio M, Leone R, Monari MN, Massari M, Cetin I, Abati S. Inflammatory and Oxidative Responses in Pregnancies With Obesity and Periodontal Disease. Reprod Sci. 2018 Oct;25(10):1474-1484. doi: 10.1177/1933719117749758. Epub 2018 Jan 17. PMID: 29343164. | No case group |
| 2018 | 75 | Yang NY, Wang CY, Chyuan IT, Wu KJ, Tu YK, Chang CW, Hsu PN, Kuo MY, Chen YW. Significant association of rheumatoid arthritis-related inflammatory markers with non-surgical periodontal therapy. J Formos Med Assoc. 2018 Nov;117(11):1003-1010. doi: 10.1016/j.jfma.2017.11.006. Epub 2017 Nov 22. PMID: 29174174. | Patients with systemic diseases |
| 2018 | 76 | Górski B, Górska R. The impact of periodontal treatment on inflammatory markers and cellular parameters associated with atherosclerosis in patients after myocardial infarction. Cent Eur J Immunol. 2018;43(4):442-452. doi: 10.5114/ceji.2018.81356. Epub 2018 Dec 31. PMID: 30799993; PMCID: PMC6384430. | Patients with systemic diseases |
| 2018 | 77 | Suryaprasanna J, Radhika PL, Karunakar P, Rekharani K, Faizuddin U, Manojkumar MG, Jammula S. Evaluating the effectiveness of clarithromycin as an adjunct to scaling and root planing: A randomized clinical trial. J Indian Soc Periodontol. 2018 Nov-Dec;22(6):529-534. doi: 10.4103/jisp.jisp_254_18. PMID: 30631232; PMCID: PMC6305085. | Unclear method of CRP measurement |
| 2018 | 78 | Tayman MA, Önder C, Kurgan Ş, Serdar MA, Günhan M. A Novel Systemic Indicator of Periodontal Tissue Damage: Ischemia Modified Albumin. Comb Chem High Throughput Screen. 2018;21(8):544-549. doi: 10.2174/1386207321666181018165255. PMID: 30338733. | Unable to access |
| 2018 | 79 | Bazyar H, Gholinezhad H, Moradi L, Salehi P, Abadi F, Ravanbakhsh M, Zare Javid A. The effects of melatonin supplementation in adjunct with non-surgical periodontal therapy on periodontal status, serum melatonin and inflammatory markers in type 2 diabetes mellitus patients with chronic periodontitis: a double-blind, placebo-controlled trial. Inflammopharmacology. 2019 Feb;27(1):67-76. doi: 10.1007/s10787-018-0539-0. Epub 2018 Oct 16. PMID: 30328031. | Patients with systemic diseases |
| 2018 | 80 | Zhao X, Liu Z, Shu D, Xiong Y, He M, Xu S, Si S, Guo B. Association of Periodontitis with Rheumatoid Arthritis and the Effect of Non-Surgical Periodontal Treatment on Disease Activity in Patients with Rheumatoid Arthritis. Med Sci Monit. 2018 Aug 20;24:5802-5810. doi: 10.12659/MSM.909117. PMID: 30124222; PMCID: PMC6113853. | Patients with systemic diseases |
| 2018 | 81 | Ahmad A, Nazar Z, Swaminathan D. C-Reactive Protein Levels and Periodontal Diseases During Pregnancy in Malaysian Women. Oral Health Prev Dent. 2018;16(3):281-289. doi: 10.3290/j.ohpd.a40759. PMID: 30027167. | Patients with systemic diseases |
| 2018 | 82 | Quintero AJ, Chaparro A, Quirynen M, Ramirez V, Prieto D, Morales H, Prada P, Hernández M, Sanz A. Effect of two periodontal treatment modalities in patients with uncontrolled type 2 diabetes mellitus: A randomized clinical trial. J Clin Periodontol. 2018 Sep;45(9):1098-1106. doi: 10.1111/jcpe.12991. Epub 2018 Aug 16. PMID: 30024030. | No mean or/and standard deviation values |
| 2018 | 83 | Ramich T, Asendorf A, Nickles K, Oremek GM, Schubert R, Nibali L, Wohlfeil M, Eickholz P. Inflammatory serum markers up to 5 years after comprehensive periodontal therapy of aggressive and chronic periodontitis. Clin Oral Investig. 2018 Dec;22(9):3079-3089. doi: 10.1007/s00784-018-2398-x. Epub 2018 Feb 27. PMID: 29484548; PMCID: PMC6224024. | Data in thresholds |
| 2017 | 84 | Al-Zahrani MS, Abozor BM, Zawawi KH. The relationship between periapical lesions and the serum levels of glycosylated hemoglobin and C-reactive protein in type 2 diabetic patients. Saudi Med J. 2017 Jan;38(1):36-40. doi: 10.15537/smj.2017.1.16052. PMID: 28042628; PMCID: PMC5278063. | Patients with systemic diseases |
| 2017 | 85 | Äyräväinen L, Leirisalo-Repo M, Kuuliala A, Ahola K, Koivuniemi R, Meurman JH, Heikkinen AM. Periodontitis in early and chronic rheumatoid arthritis: a prospective follow-up study in Finnish population. BMJ Open. 2017 Jan 31;7(1):e011916. | Patients with systemic diseases |
| 2017 | 86 | Bolla V, Kumari P S, Munnangi SR, Kumar D S, Durgabai Y, Koppolu P. Evaluation of serum c-reactive protein levels in subjects with aggressive and chronic periodontitis in comparison with healthy controls: A clinico-biochemical study. Int J App Basic Med Res 2017;7:121-4 | Unclear periodontitis criteria |
| 2017 | 87 | Çalapkorur MU, Alkan BA, Tasdemir Z, Akcali Y, Saatçi E. Association of peripheral arterial disease with periodontal disease: analysis of inflammatory cytokines and an acute phase protein in gingival crevicular fluid and serum. J Periodontal Res. 2017 Jun;52(3):532-539. | Patients with systemic diseases |
| 2017 | 88 | Cotič J, Ferran M, Karišik J, Jerin A, Pussinen PJ, Nemec A, Pavlica Z, Buturović-Ponikvar J, Petelin M. Oral health and systemic inflammatory, cardiac and nitroxid biomarkers in hemodialysis patients. Med Oral Patol Oral Cir Bucal. 2017 Jul 1;22(4):e432-e439. doi: 10.4317/medoral.21629. PMID: 28578371; PMCID: PMC5549516. | Patients with systemic diseases |
| 2017 | 89 | Ebersole JL, Kryscio RJ, Campbell C, Kinane DF, McDevitt J, Christodoulides N, Floriano PN, Miller CS. Salivary and serum adiponectin and C-reactive protein levels in acute myocardial infarction related to body mass index and oral health. J Periodontal Res. 2017 Jun;52(3):419-427. | Patients with systemic diseases |
| 2017 | 90 | Esteves Lima RP, Cota LO, Silva TA, Cortelli SC, Cortelli JR, Costa FO. Periodontitis and type 2 diabetes among women with previous gestational diabetes: epidemiological and immunological aspects in a follow-up of three years. J Appl Oral Sci. 2017 Mar-Apr;25(2):130-139. doi: 10.1590/1678-77572016-0367. PMID: 28403353; PMCID: PMC5393533. | Patients with systemic diseases |
| 2017 | 91 | Fentoğlu Ö, Dinç G, Bağcı Ö, Doğru A, İlhan I, Kırzıoğlu FY, Orhan H. R202Q/M694V as novel MEFV gene mutations in chronic periodontitis and familial Mediterranean fever. J Periodontal Res. 2017 Dec;52(6):994-1003. doi: 10.1111/jre.12467. Epub 2017 Jun 7. PMID: 28590056. | Patients with systemic diseases |
| 2017 | 92 | Gamsiz-Isik H, Kiyan E, Bingol Z, Baser U, Ademoglu E, Yalcin F. Does Obstructive Sleep Apnea Increase the Risk for Periodontal Disease? A Case-Control Study. J Periodontol. 2017 May;88(5):443-449. doi: 10.1902/jop.2016.160365. Epub 2016 Nov 18. PMID: 27858556. | Patients with systemic diseases |
| 2017 | 93 | Gupta S, Pradhan S, Kc S, Shakya S, Giri M. C-reactive Protein in Periodontitis and its Comparison with Body Mass Index and Smoking Behaviour. JNMA J Nepal Med Assoc. 2017 Apr-Jun;56(206):226-233. | Unclear periodontitis criteria |
| 2017 | 94 | Hou Y, Wang X, Zhang CX, Wei YD, Jiang LL, Zhu XY, Du YJ. Risk factors of periodontal disease in maintenance hemodialysis patients. Medicine (Baltimore). 2017 Sep;96(35):e7892. | Patients with systemic diseases |
| 2017 | 95 | Lund Håheim, L., Rønningen, K. S., Enersen, M., & Olsen, I. (2017). The Predictive Role of Tooth Extractions, Oral Infections, and hs-C-Reactive Protein for Mortality in Individuals with and without Diabetes: A Prospective Cohort Study of a 12 1/2-Year Follow-Up. Journal of Diabetes Research, 2017, 1–9. doi:10.1155/2017/9590740 | Only males included |
| 2017 | 96 | Maruyama T, Tomofuji T, Machida T, Kato H, Tsutsumi K, Uchida D, Takaki A, Yoneda T, Miyai H, Mizuno H, Ekuni D, Okada H, Morita M. Association between periodontitis and prognosis of pancreatobiliary tract cancer: A pilot study. Mol Clin Oncol. 2017 May;6(5):683-687. doi: 10.3892/mco.2017.1220. Epub 2017 Apr 10. PMID: 28515921; PMCID: PMC5431377. | Patients with systemic diseases |
| 2017 | 97 | Meisel, P., Kohlmann, T., Nauck, M., Biffar, R., & Kocher, T. (2016). Effect of body shape and inflammation on tooth loss in men and women. Clinical Oral Investigations, 21(1), 183–190. doi:10.1007/s00784-016-1775-6 | Unrelated |
| 2017 | 98 | Meurman JH, Janket SJ, Surakka M, Jackson EA, Ackerson LK, Fakhri HR, Chogle S, Walls A. Lower risk for cardiovascular mortality for patients with root filled teeth in a Finnish population. Int Endod J. 2017 Dec;50(12):1158-1168. doi: 10.1111/iej.12772. Epub 2017 May 2. PMID: 28332718. | Patients with systemic diseases |
| 2017 | 99 | Rashmi N, Galhotra V, Goel P, Rajguru JP, Jha SK, Kulkarni K. Assessment of C-reactive Proteins, Cytokines, and Plasma Protein Levels in Hypertensive Patients with Apical Periodontitis. J Contemp Dent Pract. 2017 Jun 1;18(6):516-521. doi: 10.5005/jp-journals-10024-2076. PMID: 28621285. | Patients with systemic diseases |
| 2017 | 100 | Romero-Sanchez C, Rodríguez C, Santos-Moreno P, Mesa AM, Lafaurie GI, Giraldo-Q S, De-Avila J, Castillo DM, Duran M, Chalem PC, Bello Gualtero JM, Valle-Oñate R. Is the Treatment with Biological or Non-biological DMARDS a Modifier of Periodontal Condition in Patients with Rheumatoid Arthritis? Curr Rheumatol Rev. 2017;13(2):139-151. doi: 10.2174/1573397113666170407161520. PMID: 28403797. | Patients with systemic diseases |
| 2017 | 101 | Romero-Sánchez C, Malagón C, Vargas C, Fernanda Torres M, Moreno LC, Rodríguez C, Castillo DM, De Avila J, Mosquera ÁC, Lafaurie GI. Porphyromonas Gingivalis and IgG1 and IgG2 Subclass Antibodies in Patients with Juvenile Idiopathic Arthritis. J Dent Child (Chic). 2017 May 15;84(2):72-79. PMID: 28814366. | Patients with systemic diseases |
| 2017 | 102 | Thanakun S, Pornprasertsuk-Damrongsri S, Izumi Y. Increased oral inflammation, leukocytes, and leptin, and lower adiponectin in overweight or obesity. Oral Dis. 2017 Oct;23(7):956-965. | Patients with systemic diseases |
| 2017 | 103 | Vadakkekuttical, R. J., Kaushik, P. C., Mammen, J., & George, J. M. (2017). Does periodontal inflammation affect glycosylated haemoglobin level in otherwise systemically healthy individuals? – A hospital based study. Singapore Dental Journal, 38, 55–61. doi:10.1016/j.sdj.2017.08.002 | Data in thresholds |
| 2017 | 104 | Vedin O, Hagström E, Östlund O, Avezum A, Budaj A, Flather MD, Harrington RA, Koenig W, Soffer J, Siegbahn A, Steg PG, Stewart RAH, Wallentin L, White HD, Held C; STABILITY Investigators. Associations between tooth loss and prognostic biomarkers and the risk for cardiovascular events in patients with stable coronary heart disease. Int J Cardiol. 2017 Oct 15;245:271-276. doi: 10.1016/j.ijcard.2017.07.036. Epub 2017 Jul 17. PMID: 28735759. | Patients with systemic diseases |
| 2017 | 105 | Kapellas K, Mejia G, Bartold PM, Skilton MR, Maple-Brown LJ, Slade GD, O'Dea K, Brown A, Celermajer DS, Jamieson LM. Periodontal therapy and glycaemic control among individuals with type 2 diabetes: reflections from the PerioCardio study. Int J Dent Hyg. 2017 Nov;15(4):e42-e51. doi: 10.1111/idh.12234. Epub 2016 Jun 1. PMID: 27245786. | Patients with systemic diseases |
| 2017 | 106 | Deepti, Tewari S, Narula SC, Singhal SR, Sharma RK. Effect of Non-Surgical Periodontal Therapy Along With Myo-Inositol on High-Sensitivity C-Reactive Protein and Insulin Resistance in Women With Polycystic Ovary Syndrome and Chronic Periodontitis: A Randomized Controlled Trial. J Periodontol. 2017 Oct;88(10):999-1011. doi: 10.1902/jop.2017.170121. Epub 2017 Jun 9. PMID: 28598285. | Patients with systemic diseases |
| 2017 | 107 | Hayashi J, Hasegawa A, Hayashi K, Suzuki T, Ishii M, Otsuka H, Yatabe K, Goto S, Tatsumi J, Shin K. Effects of periodontal treatment on the medical status of patients with type 2 diabetes mellitus: a pilot study. BMC Oral Health. 2017 Apr 21;17(1):77. doi: 10.1186/s12903-017-0369-2. PMID: 28431542; PMCID: PMC5399866. | Patients with systemic diseases |
| 2016 | 108 | Anwar A, Qaisar AM, Akbar S (2016). Association of C-Reactive Protein Levels With Periodontitis and Type II Diabetes Mellitus. PJMHS, 10(2), 608-610. | Unclear periodontitis criteria |
| 2016 | 109 | Bello-Gualtero JM, Lafaurie GI, Hoyos LX, Castillo DM, De-Avila J, Munevar JC, Unriza S, Londoño J, Valle-Oñate R, Romero-Sánchez C. Periodontal Disease in Individuals With a Genetic Risk of Developing Arthritis and Early Rheumatoid Arthritis: A Cross-Sectional Study. J Periodontol. 2016 Apr;87(4):346-56. doi: 10.1902/jop.2015.150455. Epub 2015 Nov 26. PMID: 26609697. | Patients with systemic diseases |
| 2016 | 110 | Górski B, Nargiełło E, Opolski G, Ganowicz E, Górska R. The Association Between Dental Status and Systemic Lipid Profile and Inflammatory Mediators in Patients After Myocardial Infarction. Adv Clin Exp Med. 2016 Jul-Aug;25(4):625-30. doi: 10.17219/acem/62937. PMID: 27629835. | Patients with systemic diseases |
| 2016 | 111 | Khare N, Vanza B, Sagar D, Saurav K, Chauhan R, Mishra S. Nonsurgical Periodontal Therapy decreases the Severity of Rheumatoid Arthritis: A Case-control Study. J Contemp Dent Pract. 2016 Jun 1;17(6):484-8. | Patients with systemic diseases |
| 2016 | 112 | Liu B, Li Y, Luo J, Dai L, Zhao J, Li H, Jie Q, Wang D, Huang X, Wei Y. Low protein Z plasma level is a risk factor for acute myocardial infarction in coronary atherosclerosis disease patients. Thromb Res. 2016 Dec;148:25-31. doi: 10.1016/j.thromres.2016.10.010. Epub 2016 Oct 13. PMID: 27770663; PMCID: PMC5154850. | Patients with systemic diseases |
| 2016 | 113 | Mannava P, Gokhale S, Pujari S, Biswas KP, Kaliappan S, Vijapure S. Comparative Evaluation of C-reactive Proteins in Pregnant Women with and without Periodontal Pathologies: A Prospective Cohort Analysis. J Contemp Dent Pract. 2016 Jun 1;17(6):480-3. | Patients with systemic diseases |
| 2016 | 114 | Raga LG, Mínguez I, Caffesse R, Llambés F. Changes in Periodontal Parameters and C-Reactive Protein After Pregnancy. J Periodontol. 2016 Dec;87(12):1388-1395. doi: 10.1902/jop.2016.160093. Epub 2016 Jul 1. PMID: 27367423. | Unsuitable control group |
| 2016 | 115 | Redman RS, Kerr GS, Payne JB, Mikuls TR, Huang J, Sayles HR, Becker KL, Nylén ES. Salivary and serum procalcitonin and C-reactive protein as biomarkers of periodontitis in United States veterans with osteoarthritis or rheumatoid arthritis. Biotech Histochem. 2016;91(2):77-85. | Patients with systemic diseases |
| 2016 | 116 | Wang Y, Sugita N, Yoshihara A, Iwasaki M, Miyazaki H, Nakamura K, Yoshie H. PPARγ gene polymorphism, C-reactive protein level, BMI and periodontitis in post-menopausal Japanese women. Gerodontology. 2016 Mar;33(1):44-51. | Unsuitable control group |
| 2016 | 117 | Vidal F, Fontes TV, Marques TV, Gonçalves LS. Association between apical periodontitis lesions and plasmatic levels of C-reactive protein, interleukin 6 and fibrinogen in hypertensive patients. Int Endod J. 2016 Dec;49(12):1107-1115. | Patients with systemic diseases |
| 2016 | 118 | Thanakun S, Izumi Y. Effect of Periodontitis on Adiponectin, C-Reactive Protein, and Immunoglobulin G Against Porphyromonas gingivalis in Thai People With Overweight or Obese Status. J Periodontol. 2016 May;87(5):566-76. | Patients with systemic diseases |
| 2016 | 119 | Thanakun, S., Pornprasertsuk-Damrongsri, S., Gokyu, M., Kobayashi, H., & Izumi, Y. (2016). Inverse Association of Plasma IgG Antibody to Aggregatibacter actinomycetemcomitans and High C-Reactive Protein Levels in Patients with Metabolic Syndrome and Periodontitis. PLOS ONE, 11(2), e0148638. doi:10.1371/journal.pone.0148638 | No mean or/and standard deviation values |
| 2016 | 120 | Zanella SM, Pereira SS, Barbisan JN, Vieira L, Saba-Chujfi E, Haas AN, Rösing CK. Periodontal disease, tooth loss and coronary heart disease assessed by coronary angiography: a cross-sectional observational study. J Periodontal Res. 2016 Apr;51(2):221-7. doi: 10.1111/jre.12301. Epub 2015 Jul 30. PMID: 26223630. | Patients with systemic diseases |
| 2016 | 121 | Žekonis, G., Žekonis, J., Gleiznys, A., Noreikienė, V., Balnytė, I., Šadzevičienė, R., & Narbutaitė, J. (2016). Effect of Supragingival Irrigation with Aerosolized 0.5% Hydrogen Peroxide on Clinical Periodontal Parameters, Markers of Systemic Inflammation, and Morphology of Gingival Tissues in Patients with Periodontitis. Medical Science Monitor, 22, 3713–3721. doi:10.12659/msm.900338 | No control group |
| 2016 | 122 | Zuza EP, Barroso EM, Fabricio M, Carrareto AL, Toledo BE, R Pires J. Lipid profile and high-sensitivity C-reactive protein levels in obese and non-obese subjects undergoing non-surgical periodontal therapy. J Oral Sci. 2016;58(3):423-30. doi: 10.2334/josnusd.16-0173. PMID: 27665983. | Patients with systemic diseases |
| 2016 | 123 | Izuora KE, Ezeanolue EE, Neubauer MF, Gewelber CL, Allenback GL, Shan G, Umpierrez GE. Changes in Inflammatory and Bone Turnover Markers After Periodontal Disease Treatment in Patients With Diabetes. Am J Med Sci. 2016 Jun;351(6):589-94. doi: 10.1016/j.amjms.2016.02.004. Epub 2016 Apr 23. PMID: 27238921; PMCID: PMC4886108. | Patients with systemic diseases |
| 2016 | 124 | Geisinger ML, Michalowicz BS, Hou W, Schoenfeld E, Gelato M, Engebretson SP, Reddy MS, Hyman L. Systemic Inflammatory Biomarkers and Their Association With Periodontal and Diabetes-Related Factors in the Diabetes and Periodontal Therapy Trial, A Randomized Controlled Trial. J Periodontol. 2016 Aug;87(8):900-13. doi: 10.1902/jop.2016.150727. Epub 2016 Apr 25. PMID: 27108476. | Patients with systemic diseases |
| 2016 | 125 | Giannopoulou C, Cionca N, Almaghlouth A, Cancela J, Courvoisier DS, Mombelli A. Systemic Biomarkers in 2-Phase Antibiotic Periodontal Treatment: A Randomized Clinical Trial. J Dent Res. 2016 Mar;95(3):349-55. doi: 10.1177/0022034515618949. Epub 2015 Nov 24. PMID: 26604272. | No mean or/and standard deviation values |
| 2016 | 126 | Srirangarajan S, Setty R, Satyanarayan A, Shetty S. Effect of full-mouth disinfection on insulin sensitivity in type 2 diabetes patients with and without chronic periodontitis. Quintessence Int. 2016 Feb;47(2):103-12. doi: 10.3290/j.qi.a34811. PMID: 26417619. | Patients with systemic diseases |
| 2016 | 127 | Ren J, Chen YB, Zhang YY, Zhou QB, Chen S, Yang JY, Tao J. Decreased circulating neopterin is associated with increased arterial elasticity: a beneficial role of periodontal treatment. Aust Dent J. 2016 Mar;61(1):76-83. doi: 10.1111/adj.12303. PMID: 25600514. | Unsuitable baseline CRP data |
| 2015 | 128 | Awad M, Rahman B, Hasan H, Ali H. The Relationship between Body Mass Index and Periodontitis in Arab Patients with Type 2 Diabetes Mellitus. Oman Med J. 2015 Jan;30(1):36-41. doi: 10.5001/omj.2015.07. PMID: 25829999; PMCID: PMC4371459. | Patients with systemic diseases |
| 2015 | 129 | Amaliya, A., Laine, M. L., Delanghe, J. R., Loos, B. G., Van Wijk, A. J., & Van der Velden, U. (2015). Java project on periodontal diseases: periodontal bone loss in relation to environmental and systemic conditions. Journal of Clinical Periodontology, 42(4), 325–332. doi:10.1111/jcpe.12381 | Unrelated |
| 2015 | 130 | Andriankaja OM, Jiménez JJ, Muñoz-Torres FJ, Pérez CM, Vergara JL, Joshipura KJ. Lipid-lowering agents use and systemic and oral inflammation in overweight or obese adult Puerto Ricans: the San Juan Overweight Adults Longitudinal Study (SOALS). J Clin Periodontol. 2015 Dec;42(12):1090-6. | Patients with systemic diseases |
| 2015 | 131 | Bhavsar NV, Dave BD, Brahmbhatt NA, Parekh R. Periodontal status and oral health behavior in hospitalized patients with chronic obstructive pulmonary disease. J Nat Sci Biol Med. 2015 Aug;6(Suppl 1):S93-7. doi: 10.4103/0976-9668.166097. PMID: 26604629; PMCID: PMC4630773. | Patients with systemic diseases |
| 2015 | 132 | Cutando A, Montero J, Gómez-de Diego R, Ferrera MJ, Lopez-Valverde A. Effect of topical application of melatonin on serum levels of C-reactive protein (CRP), interleukin-6 (IL-6) and tumor necrosis factor-alpha (TNF-α) in patients with type 1 or type 2 diabetes and periodontal disease. J Clin Exp Dent. 2015 Dec 1;7(5):e628-33. doi: 10.4317/jced.52604. PMID: 26644840; PMCID: PMC4663066. | Patients with systemic diseases |
| 2015 | 133 | Fabri GM, Pereira RM, Savioli C, Saad CG, de Moraes JC, Siqueira JT, Bonfa E. Periodontitis Response to Anti-TNF Therapy in Ankylosing Spondylitis. J Clin Rheumatol. 2015 Oct;21(7):341-5. doi: 10.1097/RHU.0000000000000300. PMID: 26398459. | Patients with systemic diseases |
| 2015 | 134 | Furuta, M., Shimazaki, Y., Tanaka, S., Takeuchi, K., Shibata, Y., Takeshita, T., … Yamashita, Y. (2015). Gender-Specific Associations of Serum Antibody toPorphyromonas gingivalisand Inflammatory Markers. BioMed Research International, 2015, 1–9. doi:10.1155/2015/897971 | No mean or/and standard deviation values |
| 2015 | 135 | Garneata L, Slusanschi O, Preoteasa E, Corbu-Stancu A, Mircescu G. Periodontal status, inflammation, and malnutrition in hemodialysis patients - is there a link? J Ren Nutr. 2015 Jan;25(1):67-74. doi: 10.1053/j.jrn.2014.07.004. Epub 2014 Sep 11. | Patients with systemic diseases |
| 2015 | 136 | Gonzalez SM, Payne JB, Yu F, Thiele GM, Erickson AR, Johnson PG, Schmid MJ, Cannon GW, Kerr GS, Reimold AM, Sokolove J, Robinson WH, Mikuls TR. Alveolar bone loss is associated with circulating anti-citrullinated protein antibody (ACPA) in patients with rheumatoid arthritis. J Periodontol. 2015 Feb;86(2):222-31. doi: 10.1902/jop.2014.140425. Epub 2014 Oct 9. PMID: 25299390. | Patients with systemic diseases |
| 2015 | 137 | Kiedrowicz M, Dembowska E, Banach J, Safranow K, Pynka S. A comparison of the periodontal status in patients with type 2 diabetes based on glycated haemoglobin levels and other risk factors. Adv Med Sci. 2015 Mar;60(1):156-61. | Patients with systemic diseases |
| 2015 | 138 | Kobayashi T, Ito S, Kobayashi D, Kojima A, Shimada A, Narita I, Murasawa A, Nakazono K, Yoshie H. Interleukin-6 receptor inhibitor tocilizumab ameliorates periodontal inflammation in patients with rheumatoid arthritis and periodontitis as well as tumor necrosis factor inhibitors. Clin Exp Dent Res. 2015 Nov 13;1(2):63-73. doi: 10.1002/cre2.11. PMID: 29744142; PMCID: PMC5839195. | Patients with systemic diseases |
| 2015 | 139 | Kimura Y, Yoshida S, Takeuchi T, Kimura M, Yoshikawa A, Hiramatsu Y, Ishida T, Makino S, Takasugi Y, Hanafusa T. Periodontal pathogens participate in synovitis in patients with rheumatoid arthritis in clinical remission: a retrospective case-control study. Rheumatology (Oxford). 2015 Dec;54(12):2257-63. doi: 10.1093/rheumatology/kev274. Epub 2015 Aug 5. PMID: 26248812. | Patients with systemic diseases |
| 2015 | 140 | Wang CY, Chyuan IT, Wang YL, Kuo MY, Chang CW, Wu KJ, Hsu PN, Nagasawa T, Wara-aswapati N, Chen YW. β2-Glycoprotein I-Dependent Anti-Cardiolipin Antibodies Associated With Periodontitis in Patients With Systemic Lupus Erythematosus. J Periodontol. 2015 Aug;86(8):995-1004. | Unsuitable control group |
| 2015 | 141 | Sanders AE, Essick GK, Beck JD, Cai J, Beaver S, Finlayson TL, Zee PC, Loredo JS, Ramos AR, Singer RH, Jimenez MC, Barnhart JM, Redline S. Periodontitis and Sleep Disordered Breathing in the Hispanic Community Health Study/Study of Latinos. Sleep. 2015 Aug 1;38(8):1195-203. | Patients with systemic diseases |
| 2015 | 142 | Seringec, N., Guncu, G., Arihan, O., Avcu, N., & Dikmenoglu, N. (2015). Investigation of hemorheological parameters in periodontal diseases. Clinical Hemorheology and Microcirculation, 61(1), 47–58. doi:10.3233/ch-141892 | Only males included |
| 2015 | 143 | Singer, R. E., Moss, K., Kim, S. J., Beck, J. D., & Offenbacher, S. (2015). Oxidative Stress and IgG Antibody Modify Periodontitis-CRP Association. Journal of Dental Research, 94(12), 1698–1705. doi:10.1177/0022034515602693 | Unrelated |
| 2015 | 144 | Winning, L., Patterson, C. C., Cullen, K. M., Stevenson, K. A., Lundy, F. T., Kee, F., & Linden, G. J. (2015). The association between subgingival periodontal pathogens and systemic inflammation. Journal of Clinical Periodontology, 42(9), 799–806. doi:10.1111/jcpe.12450 | No serum CRP values |
| 2015 | 145 | Roman-Torres CV, Neto JS, Souza MA, Schwartz-Filho HO, Brandt WC, Diniz RE. An Evaluation of Non-Surgical Periodontal Therapy in Patients with Rheumatoid Arthritis. Open Dent J. 2015 May 15;9:150-3. doi: 10.2174/1874210601509010150. PMID: 26140059; PMCID: PMC4484346. | Unclear method of CRP measurement |
| 2015 | 146 | Vedin O, Hagström E, Gallup D, Neely ML, Stewart R, Koenig W, Budaj A, Sritara P, Wallentin L, White HD, Held C. Periodontal disease in patients with chronic coronary heart disease: Prevalence and association with cardiovascular risk factors. Eur J Prev Cardiol. 2015 Jun;22(6):771-8. doi: 10.1177/2047487314530660. Epub 2014 Apr 10. PMID: 24721691. | Patients with systemic diseases |
| 2015 | 147 | Tawfig A. Effects of non-surgical periodontal therapy on serum lipids and C-reactive protein among hyperlipidemic patients with chronic periodontitis. J Int Soc Prev Community Dent. 2015 May;5(Suppl 1):S49-56. | Undefined follow-up period |
| 2015 | 148 | Zhang Z, Ma N, Zheng Y, Zhang L. Association of serum immunoglobulin-G to Porphyromonas gingivalis with acute cerebral infarction in the Chinese population. J Indian Soc Periodontol. 2015 Nov-Dec;19(6):628-32. doi: 10.4103/0972-124X.164750. PMID: 26941512; PMCID: PMC4753706. | Patients with systemic diseases |
| 2015 | 149 | Hada DS, Garg S, Ramteke GB, Ratre MS. Effect of Non-Surgical Periodontal Treatment on Clinical and Biochemical Risk Markers of Cardiovascular Disease: A Randomized Trial. J Periodontol. 2015 Nov;86(11):1201-11. doi: 10.1902/jop.2015.150249. Epub 2015 Jul 24. PMID: 26205747. | Patients with systemic diseases |
| 2015 | 150 | Tawfig A. Effects of non-surgical periodontal therapy on serum lipids and C-reactive protein among hyperlipidemic patients with chronic periodontitis. J Int Soc Prev Community Dent. 2015 May;5(Suppl 1):S49-56. doi: 10.4103/2231-0762.156524. PMID: 25984468; PMCID: PMC4428020. | Patients with systemic diseases |
| 2015 | 151 | Reddy PV, Ambati M, Koduganti R. Systemic lycopene as an adjunct to scaling and root planing in chronic periodontitis patients with type 2 diabetes mellitus. J Int Soc Prev Community Dent. 2015 May;5(Suppl 1):S25-31. doi: 10.4103/2231-0762.156520. PMID: 25984464; PMCID: PMC4428016. | Patients with systemic diseases |
| 2015 | 152 | Fang F, Wu B, Qu Q, Gao J, Yan W, Huang X, Ma D, Yue J, Chen T, Liu F, Liu Y. The clinical response and systemic effects of non-surgical periodontal therapy in end-stage renal disease patients: a 6-month randomized controlled clinical trial. J Clin Periodontol. 2015 Jun;42(6):537-46. doi: 10.1111/jcpe.12411. Epub 2015 May 30. PMID: 25933364. | Patients with systemic diseases |
| 2015 | 153 | Khairnar MS, Pawar BR, Marawar PP, Khairnar DM. Estimation of changes in C-reactive protein level and pregnancy outcome after nonsurgical supportive periodontal therapy in women affected with periodontitis in a rural set up of India. Contemp Clin Dent. 2015 Mar;6(Suppl 1):S5-S11. doi: 10.4103/0976-237X.152930. PMID: 25821375; PMCID: PMC4374319. | Patients with systemic diseases |
| 2014 | 154 | Arregoces FE, Uriza CL, Porras JV, Camargo MB, Morales AR. Relation between ultra-sensitive C-reactive protein, diabetes and periodontal disease in patients with and without myocardial infarction. Arq Bras Endocrinol Metabol. 2014 Jun;58(4):362-8. doi: 10.1590/0004-2730000002899. PMID: 24936730. | Patients with systemic diseases |
| 2014 | 155 | Baser U, Oztekin G, Ademoglu E, Isik G, Yalcin F (2014). Is the Severity of Periodontitis Related to Gingival Crevicular Fluid and Serum High-Sensitivity C-reactive Protein Concentrations? Clin Lab, 60 (10), 1653-8. 10.7754/clin.lab.2014.131217 | Unable to access |
| 2014 | 156 | Bokhari SA, Khan AA, Butt AK, Hanif M, Izhar M, Tatakis DN, Ashfaq M. Periodontitis in coronary heart disease patients: strong association between bleeding on probing and systemic biomarkers. J Clin Periodontol. 2014 Nov;41(11):1048-54. | Patients with systemic diseases |
| 2014 | 157 | Bullon P, Jaramillo R, Santos-Garcia R, Rios-Santos V, Ramirez M, Fernandez-Palacin A, Fernandez-Riejos P. Relation of periodontitis and metabolic syndrome with gestational glucose metabolism disorder. J Periodontol. 2014 Feb;85(2):e1-8. | Patients with systemic diseases |
| 2014 | 158 | Buduneli N, Bıyıkoğlu B, Ilgenli T, Buduneli E, Nalbantsoy A, Saraç F, Kinane DF. Is obesity a possible modifier of periodontal disease as a chronic inflammatory process? A case-control study. J Periodontal Res. 2014 Aug;49(4):465-71. | Patients with systemic diseases |
| 2014 | 159 | El-Beshbishy HA, Maria RA, Bardi FA. Biochemical and C-reactive protein alterations in myocardial infarction periodontitis patients. Am J Med Sci. 2014 Sep;348(3):181-5. | Patients with systemic diseases |
| 2014 | 160 | Etemadifar R, Konarizadeh S, Zarei A, Farshidi H, Sobhani A. Relationship between periodontal status and C-reactive protein and interleuckin-6 levels among atherosclerotic patients in Bandar Abbas, Iran in 2014. Electron Physician. 2015 Mar 1;7(1):1010-6. doi: 10.14661/2015.1010-1016. PMID: 26052413; PMCID: PMC4455295. | Patients with systemic diseases |
| 2014 | 161 | Flores MF, Montenegro MM, Furtado MV, Polanczyk CA, Rösing CK, Haas AN. Periodontal status affects C-reactive protein and lipids in patients with stable heart disease from a tertiary care cardiovascular clinic. J Periodontol. 2014 Apr;85(4):545-53. doi: 10.1902/jop.2013.130255. Epub 2013 Jun 27. PMID: 23805809. | Patients with systemic diseases |
| 2014 | 162 | Gocke, C., Holtfreter, B., Meisel, P., Grotevendt, A., Jablonowski, L., Nauck, M., … Kocher, T. (2014). Abdominal obesity modifies long-term associations between periodontitis and markers of systemic inflammation. Atherosclerosis, 235(2), 351–357. doi:10.1016/j.atherosclerosis.2014.05.926 | No mean or/and standard deviation values |
| 2014 | 163 | Janket SJ, Baird AE, Jones JA, Jackson EA, Surakka M, Tao W, Meurman JH, Van Dyke TE. Number of teeth, C-reactive protein, fibrinogen and cardiovascular mortality: a 15-year follow-up study in a Finnish cohort. J Clin Periodontol. 2014 Feb;41(2):131-40. doi: 10.1111/jcpe.12192. Epub 2013 Dec 10. PMID: 24354534; PMCID: PMC3934352. | Patients with systemic diseases |
| 2014 | 164 | Kobayashi T, Yokoyama T, Ito S, Kobayashi D, Yamagata A, Okada M, Oofusa K, Narita I, Murasawa A, Nakazono K, Yoshie H. Periodontal and serum protein profiles in patients with rheumatoid arthritis treated with tumor necrosis factor inhibitor adalimumab. J Periodontol. 2014 Nov;85(11):1480-8. doi: 10.1902/jop.2014.140194. Epub 2014 May 26. PMID: 24857321. | Patients with systemic diseases |
| 2014 | 165 | Lafon A, Tala S, Ahossi V, Perrin D, Giroud M, Béjot Y. Association between periodontal disease and non-fatal ischemic stroke: a case-control study. Acta Odontol Scand. 2014 Nov;72(8):687-93. doi: 10.3109/00016357.2014.898089. Epub 2014 Apr 11. PMID: 24720864. | Patients with systemic diseases |
| 2014 | 166 | Leite, Anne Carolina Eleutério, Carneiro, Valéria Martins de Araújo, & Guimarães, Maria do Carmo Machado. (2014). Effects of periodontal therapy on C-reactive protein and HDL in serum of subjects with periodontitis. Brazilian Journal of Cardiovascular Surgery, 29(1), 69-77. https://dx.doi.org/10.5935/1678-9741.20140013 | No mean or/and standard deviation values |
| 2014 | 167 | Lakshmanan, R., Jayakumar, N. D., Sankari, M., Padmalatha, O., & Varghese, S. (2014). Estimation of Pentraxin-3 Levels in the Gingival Tissues of Chronic and Aggressive Periodontitis Participants: An In Vivo Study. Journal of Periodontology, 85(2), 290–297. doi:10.1902/jop.2013.120718 | No serum CRP values |
| 2014 | 168 | Magán-Fernández A, Papay-Ramírez L, Tomás J, Marfil-Álvarez R, Rizzo M, Bravo M, Mesa F. Association of simvastatin and hyperlipidemia with periodontal status and bone metabolism markers. J Periodontol. 2014 Oct;85(10):1408-15. doi: 10.1902/jop.2014.130652. Epub 2014 Feb 21. PMID: 24555750. | Patients with systemic diseases |
| 2014 | 169 | Musalaiah SV, Anupama M, Nagasree M, Krishna ChM, Kumar A, Kumar PM. Evaluation of nonsurgical periodontal therapy in chronic periodontitis patients with anemia by estimating hematological parameters and high-sensitivity C-reactive protein levels. J Pharm Bioallied Sci. 2014 Jul;6(Suppl 1):S64-9. | Patients with systemic diseases |
| 2014 | 170 | Mikuls TR, Payne JB, Yu F, Thiele GM, Reynolds RJ, Cannon GW, Markt J, McGowan D, Kerr GS, Redman RS, Reimold A, Griffiths G, Beatty M, Gonzalez SM, Bergman DA, Hamilton BC 3rd, Erickson AR, Sokolove J, Robinson WH, Walker C, Chandad F, O'Dell JR. Periodontitis and Porphyromonas gingivalis in patients with rheumatoid arthritis. Arthritis Rheumatol. 2014 May;66(5):1090-100. doi: 10.1002/art.38348. PMID: 24782175; PMCID: PMC4115329. | Patients with systemic diseases |
| 2014 | 171 | Öztekin G, Baser U, Kucukcoskun M, Tanrikulu-Kucuk S, Ademoglu E, Isik G, Ozkan G, Yalcin F, Kiyan E. The association between periodontal disease and chronic obstructive pulmonary disease: a case control study. COPD. 2014 Aug;11(4):424-30. | Patients with systemic diseases |
| 2014 | 172 | Ollikainen E, Saxlin T, Tervonen T, Suominen AL, Knuuttila M, Jula A, Ylöstalo P. Association between periodontal condition and hypertension in a non-smoking population aged 30-49 years: results of the Health 2000 Survey in Finland. J Clin Periodontol. 2014 Dec;41(12):1132-8. | Patients with systemic diseases |
| 2014 | 173 | Porwal S, Tewari S, Sharma RK, Singhal SR, Narula SC. Periodontal status and high-sensitivity C-reactive protein levels in polycystic ovary syndrome with and without medical treatment. J Periodontol. 2014 Oct;85(10):1380-9. | Patients with systemic diseases |
| 2014 | 174 | Suzuki J, Imai Y, Aoki M, Fujita D, Aoyama N, Tada Y, Wakayama K, Akazawa H, Izumi Y, Isobe M, Komuro I, Nagai R, Hirata Y. Periodontitis in cardiovascular disease patients with or without Marfan syndrome--a possible role of Prevotella intermedia. PLoS One. 2014 Apr 18;9(4):e95521. doi: 10.1371/journal.pone.0095521. PMID: 24748407; PMCID: PMC3991676. | Patients with systemic diseases |
| 2014 | 175 | Yokoyama T, Kobayashi T, Ito S, Yamagata A, Ishida K, Okada M, Oofusa K, Murasawa A, Yoshie H. Comparative analysis of serum proteins in relation to rheumatoid arthritis and chronic periodontitis. J Periodontol. 2014 Jan;85(1):103-12. doi: 10.1902/jop.2013.120741. Epub 2013 May 7. PMID: 23646852. | Patients with systemic diseases |
| 2014 | 176 | Willershausen I, Weyer V, Peter M, Weichert C, Kasaj A, Münzel T, Willershausen B. Association between chronic periodontal and apical inflammation and acute myocardial infarction. Odontology. 2014 Jul;102(2):297-302. | Patients with systemic diseases |
| 2014 | 177 | Kocyigit I, Yucel HE, Cakmak O, Dogruel F, Durukan DB, Korkar H, Unal A, Sipahioglu MH, Oymak O, Gurgan CA, Tokgoz B. An ignored cause of inflammation in patients undergoing continuous ambulatory peritoneal dialysis: periodontal problems. Int Urol Nephrol. 2014 Oct;46(10):2021-8. doi: 10.1007/s11255-014-0716-z. Epub 2014 Apr 23. PMID: 24756531. | Patients with systemic diseases |
| 2014 | 178 | Raman RP, Taiyeb-Ali TB, Chan SP, Chinna K, Vaithilingam RD. Effect of nonsurgical periodontal therapy verses oral hygiene instructions on type 2 diabetes subjects with chronic periodontitis: a randomised clinical trial. BMC Oral Health. 2014 Jun 25;14:79. doi: 10.1186/1472-6831-14-79. PMID: 24965218; PMCID: PMC4082680. | Unclear method of CRP measurement |
| 2014 | 179 | Leite AC, Carneiro VM, Guimarães Mdo C. Effects of periodontal therapy on C-reactive protein and HDL in serum of subjects with periodontitis. Rev Bras Cir Cardiovasc. 2014 Jan-Mar;29(1):69-77. doi: 10.5935/1678-9741.20140013. PMID: 24896165; PMCID: PMC4389485. | Data in thresholds |
| 2014 | 180 | Wang SH, Hung HC, Tsai CC, Huang MC, Ho KY, Wu YM, Wang YY, Lin YC. Plasma polyunsaturated fatty acids and periodontal recovery in Taiwanese with periodontitis: a significant relationship. Arch Oral Biol. 2014 Aug;59(8):800-7. doi: 10.1016/j.archoralbio.2014.04.009. Epub 2014 Apr 22. PMID: 24859767. | Unclear periodontitis criteria |
| 2013 | 181 | Antonoglou G, Knuuttila M, Niemelä O, Hiltunen L, Raunio T, Karttunen R, Vainio O, Ylöstalo P, Tervonen T. Serum 1,25(OH)D level increases after elimination of periodontal inflammation in T1DM subjects. J Clin Endocrinol Metab. 2013 Oct;98(10):3999-4005. | Patients with systemic diseases |
| 2013 | 182 | Anitha G, Nagaraj M, Jayashree A. Comparative evaluation of levels of C-reactive protein and PMN in periodontitis patients related to cardiovascular disease. J Indian Soc Periodontol. 2013 May;17(3):330-2. doi: 10.4103/0972-124X.115657. PMID: 24049333; PMCID: PMC3768183. | Patients with systemic diseases |
| 2013 | 183 | Auerkari EI, Suhartono AW, Djamal NZ, Verisqa F, Suryandari DA, Kusdhany LS, Masulili SLC, Talbot C (2013). CRP and IL-1B Gene Polymorphisms and CRP in Blood in Periodontal Disease. The Open Dentistry Journal, 7, 88-93 | Only males included |
| 2013 | 184 | Buchwald S, Kocher T, Biffar R, Harb A, Holtfreter B, Meisel P. Tooth loss and periodontitis by socio-economic status and inflammation in a longitudinal population-based study. J Clin Periodontol. 2013 Mar;40(3):203-11. | Patients with systemic diseases |
| 2013 | 185 | Chaparro A, Sanz A, Quintero A, Inostroza C, Ramirez V, Carrion F, Figueroa F, Serra R, Illanes SE. Increased inflammatory biomarkers in early pregnancy is associated with the development of pre-eclampsia in patients with periodontitis: a case control study. J Periodontal Res. 2013 Jun;48(3):302-7. | Patients with systemic diseases |
| 2013 | 186 | Chokwiriyachit A, Dasanayake AP, Suwannarong W, Hormdee D, Sumanonta G, Prasertchareonsuk W, Wara-Aswapati N, Combellick J, Pitiphat W. Periodontitis and gestational diabetes mellitus in non-smoking females. J Periodontol. 2013 Jul;84(7):857-62. | Patients with systemic diseases |
| 2013 | 187 | Domingues, José Eduardo Gomes, Vettore, Mario Vianna, & Lima, Emerson Silva. (2013). Association between markers of cardiovascular risk and clinical parameters of periodontitis. Revista de Odontologia da UNESP, 42(5), 336-343. | Unsuitable control group |
| 2013 | 188 | Holtfreter B, Richter S, Kocher T, Dörr M, Völzke H, Ittermann T, Obst A, Schäper C, John U, Meisel P, Grotevendt A, Felix SB, Ewert R, Gläser S. Periodontitis is related to lung volumes and airflow limitation: a cross-sectional study. Eur Respir J. 2013 Dec;42(6):1524-35. | Patients with systemic diseases |
| 2013 | 189 | Joseph R, Rajappan S, Nath SG, Paul BJ. Association between chronic periodontitis and rheumatoid arthritis: a hospital-based case-control study. Rheumatol Int. 2013 Jan;33(1):103-9. | Unsuitable control group |
| 2013 | 190 | Katagiri S, Nitta H, Nagasawa T, Izumi Y, Kanazawa M, Matsuo A, Chiba H, Fukui M, Nakamura N, Oseko F, Kanamura N, Inagaki K, Noguchi T, Naruse K, Matsubara T, Miyazaki S, Miyauchi T, Ando Y, Hanada N, Inoue S. Effect of glycemic control on periodontitis in type 2 diabetic patients with periodontal disease. J Diabetes Investig. 2013 May;4(3):320-325. doi: 10.1111/jdi.12026. Epub 2013 Feb 14. PMID: 23997922; PMCID: PMC3752968. | Patients with systemic diseases |
| 2013 | 191 | Koppikar RS, Agrawal SV. The effect of sub-antimicrobial dose-doxycycline periodontal therapy on serum inflammatory biomarker C-reactive protein levels in post-menopausal Women: A 2-year, double-blinded, randomized clinical trial. Contemp Clin Dent. 2013 Jan;4(1):71-3. doi: 10.4103/0976-237X.111628. PMID: 23853456; PMCID: PMC3703699. | Patients with systemic diseases |
| 2013 | 192 | Noack B, Aslanhan Z, Boué J, Petig C, Teige M, Schaper F, Hoffmann T, Hannig C. Potential association of paraoxonase-1, type 2 diabetes mellitus, and periodontitis. J Periodontol. 2013 May;84(5):614-23. doi: 10.1902/jop.2012.120062. Epub 2012 Jul 6. PMID: 22769439. | Patients with systemic diseases |
| 2013 | 193 | Rajkarnikar J, Thomas BS, Rao SK. Inter- relationship between rheumatoid arthritis and periodontitis. Kathmandu Univ Med J (KUMJ). 2013 Jan-Mar;11(41):22-6. | Patients with systemic diseases |
| 2013 | 194 | Salminen A, Pussinen PJ, Payne JB, Stoner JA, Jauhiainen M, Golub LM, Lee HM, Thompson DM, Sorsa T. Subantimicrobial-dose doxycycline treatment increases serum cholesterol efflux capacity from macrophages. Inflamm Res. 2013 Jul;62(7):711-20. doi: 10.1007/s00011-013-0626-z. Epub 2013 May 7. PMID: 23649042; PMCID: PMC3700361. | Patients with systemic diseases |
| 2013 | 195 | Sen S, Sumner R, Hardin J, Barros S, Moss K, Beck J, Offenbacher S. Periodontal disease and recurrent vascular events in stroke/transient ischemic attack patients. J Stroke Cerebrovasc Dis. 2013 Nov;22(8):1420-7. doi: 10.1016/j.jstrokecerebrovasdis.2013.06.024. Epub 2013 Jul 30. PMID: 23910516; PMCID: PMC5624802. | Patients with systemic diseases |
| 2013 | 196 | Shojaee, M., Fereydooni Golpasha, M., Maliji, G., Bijani, A., Aghajanpour Mir, S. M., & Mousavi Kani, S. N. (2013). C - reactive protein levels in patients with periodontal disease and normal subjects. International journal of molecular and cellular medicine, 2(3), 151–155. | No serum CRP values |
| 2013 | 197 | Shetty S1, Bose A, Sridharan S, Satyanarayana A, Rahul A (2013). A clinico-biochemical evaluation of the role of a herbal (Ayurvedic) immunomodulator in chronic periodontal disease: a pilot study. . Oral Health Dent Manag, Jun;12(2):95-104. | No control group |
| 2013 | 198 | Susanto H, Nesse W, Kertia N, Soeroso J, Huijser van Reenen Y, Hoedemaker E, Agustina D, Vissink A, Abbas F, Dijkstra PU. Prevalence and severity of periodontitis in Indonesian patients with rheumatoid arthritis. J Periodontol. 2013 Aug;84(8):1067-74. | Patients with systemic diseases |
| 2013 | 199 | Ye C, Katagiri S, Miyasaka N, Bharti P, Kobayashi H, Takeuchi Y, Momohara Y, Sekiguchi M, Takamine S, Nagasawa T, Izumi Y. The anti-phospholipid antibody-dependent and independent effects of periodontopathic bacteria on threatened preterm labor and preterm birth. Arch Gynecol Obstet. 2013 Jul;288(1):65-72. doi: 10.1007/s00404-013-2741-z. Epub 2013 Feb 12. PMID: 23400354. | Patients with systemic diseases |
| 2013 | 200 | Tu YK, D'Aiuto F, Lin HJ, Chen YW, Chien KL. Relationship between metabolic syndrome and diagnoses of periodontal diseases among participants in a large Taiwanese cohort. J Clin Periodontol. 2013 Nov;40(11):994-1000 | Patients with systemic diseases |
| 2013 | 201 | Siribamrungwong M, Yothasamutr K, Puangpanngam K. Periodontal treatment reduces chronic systemic inflammation in peritoneal dialysis patients. Ther Apher Dial. 2014 Jun;18(3):305-8. doi: 10.1111/1744-9987.12105. Epub 2013 Sep 30. PMID: 24118730. | Patients with systemic diseases |
| 2013 | 202 | Antonoglou G, Knuuttila M, Niemelä O, Hiltunen L, Raunio T, Karttunen R, Vainio O, Ylöstalo P, Tervonen T. Serum 1,25(OH)D level increases after elimination of periodontal inflammation in T1DM subjects. J Clin Endocrinol Metab. 2013 Oct;98(10):3999-4005. doi: 10.1210/jc.2013-1906. Epub 2013 Aug 12. PMID: 23940127. | Unrelated |
| 2013 | 203 | Chauhan AS, Bains VK, Gupta V, Singh GP, Patil SS. Comparative analysis of hyaluronan gel and xanthan-based chlorhexidine gel, as adjunct to scaling and root planing with scaling and root planing alone in the treatment of chronic periodontitis: A preliminary study. Contemp Clin Dent. 2013 Jan;4(1):54-61. doi: 10.4103/0976-237X.111619. PMID: 23853453; PMCID: PMC3703695. | Unable to access |
| 2013 | 204 | Shetty S, Bose A, Sridharan S, Satyanarayana A, Rahul A. A clinico-biochemical evaluation of the role of a herbal (Ayurvedic) immunomodulator in chronic periodontal disease: a pilot study. Oral Health Dent Manag. 2013 Jun;12(2):95-104. PMID: 23756425. | Patients with systemic diseases |
| 2013 | 205 | Okada M, Kobayashi T, Ito S, Yokoyama T, Abe A, Murasawa A, Yoshie H. Periodontal treatment decreases levels of antibodies to Porphyromonas gingivalis and citrulline in patients with rheumatoid arthritis and periodontitis. J Periodontol. 2013 Dec;84(12):e74-84. doi: 10.1902/jop.2013.130079. Epub 2013 May 23. PMID: 23701010. | Incomplete CRP report levels |
| 2013 | 206 | Yazdi FK, Karimi N, Rasouli M, Roozbeh J. Effect of nonsurgical periodontal treatment on C-reactive protein levels in maintenance hemodialysis patients. Ren Fail. 2013;35(5):711-7. doi: 10.3109/0886022X.2013.777890. Epub 2013 Mar 28. PMID: 23534529. | Patients with systemic diseases |
| 2013 | 207 | Munenaga Y; Hiroshima Study Group, Yamashina T, Tanaka J, Nishimura F. Improvement of glycated hemoglobin in Japanese subjects with type 2 diabetes by resolution of periodontal inflammation using adjunct topical antibiotics: results from the Hiroshima Study. Diabetes Res Clin Pract. 2013 Apr;100(1):53-60. doi: 10.1016/j.diabres.2013.01.028. Epub 2013 Mar 7. PMID: 23465365. | Patients with systemic diseases |
| 2013 | 208 | Pradeep AR, Kalra N, Priyanka N, Kumari M, Khaneja E, Naik SB. Post-treatment levels of stem cell factor and hs-CRP in serum and crevicular fluid of chronic periodontitis subjects with type 2 diabetes. J Investig Clin Dent. 2013 May;4(2):89-93. doi: 10.1111/jicd.12008. Epub 2012 Oct 28. PMID: 23109408. | Patients with systemic diseases |
| 2013 | 209 | Zhou SY, Duan XQ, Hu R, Ouyang XY. Effect of non-surgical periodontal therapy on serum levels of TNF-a, IL-6 and C-reactive protein in periodontitis subjects with stable coronary heart disease. Chin J Dent Res. 2013;16(2):145-51. PMID: 24436950. | Patients with systemic diseases |
| 2013 | 210 | Bharti P, Katagiri S, Nitta H, Nagasawa T, Kobayashi H, Takeuchi Y, Izumiyama H, Uchimura I, Inoue S, Izumi Y. Periodontal treatment with topical antibiotics improves glycemic control in association with elevated serum adiponectin in patients with type 2 diabetes mellitus. Obes Res Clin Pract. 2013 Mar-Apr;7(2):e129-e138. doi: 10.1016/j.orcp.2011.11.005. PMID: 24331774. | Patients with systemic diseases |
| 2013 | 211 | Koppolu P, Durvasula S, Palaparthy R, Rao M, Sagar V, Reddy SK, Lingam S. Estimate of CRP and TNF-alpha level before and after periodontal therapy in cardiovascular disease patients. Pan Afr Med J. 2013 Jul 10;15:92. doi: 10.11604/pamj.2013.15.92.2326. PMID: 24198887; PMCID: PMC3810246. | Patients with systemic diseases |
| 2013 | 212 | Pabolu CM, Mutthineni RB, Chintala S, Naheeda, Mutthineni N. Evaluation of the effect of one stage versus two stage full mouth disinfection on C-reactive protein and leucocyte count in patients with chronic periodontitis. J Indian Soc Periodontol. 2013 Jul;17(4):466-71. doi: 10.4103/0972-124X.118318. PMID: 24174726; PMCID: PMC3800409. | Unclear periodontitis criteria |
| 2012 | 213 | Akman, P. T., Fentoğlu, Ö., Yılmaz, G., & Arpak, N. (2012). Serum Plasminogen Activator Inhibitor-1 and Tumor Necrosis Factor-α Levels in Obesity and Periodontal Disease. Journal of Periodontology, 83(8), 1057–1062. doi:10.1902/jop.2011.110548 | Unsuitable control group |
| 2012 | 214 | Bertl, K., Schoiber, A., Haririan, H., Laky, M., Steiner, I., Rausch, W. D., … Rausch-Fan, X. (2012). Non-surgical periodontal therapy influences salivary melatonin levels. Clinical Oral Investigations, 17(4), 1219–1225. doi:10.1007/s00784-012-0801-6 | Undefined follow-up period |
| 2012 | 215 | Demmer, R. T., Squillaro, A., Papapanou, P. N., Rosenbaum, M., Friedewald, W. T., Jacobs, D. R., & Desvarieux, M. (2012). Periodontal Infection, Systemic Inflammation, and Insulin Resistance: Results from the continuous National Health and Nutrition Examination Survey (NHANES) 1999-2004. Diabetes Care, 35(11), 2235–2242. doi:10.2337/dc12-0072 | No case group |
| 2012 | 216 | Hosomi N, Aoki S, Matsuo K, Deguchi K, Masugata H, Murao K, Ichihara N, Ohyama H, Dobashi H, Nezu T, Ohtsuki T, Yasuda O, Soejima H, Ogawa H, Izumi Y, Kohno M, Tanaka J, Matsumoto M. Association of serum anti-periodontal pathogen antibody with ischemic stroke. Cerebrovasc Dis. 2012;34(5-6):385-92. doi: 10.1159/000343659. Epub 2012 Nov 29. PMID: 23207319. | Patients with systemic diseases |
| 2012 | 217 | Esfahanian V, Abdolsafa S, Shahram J, Messripour M, Sadeghi-Dehboneh (2012). C-reactive protein levels in chronic gingivitis, chronic periodontitis and periodontally healthy subjects. Healthmed 6(9) | Unable to access |
| 2012 | 218 | Kanaparthy R, Kanaparthy A, Mahendra M (2012). C-reactive protein as a marker of periodontal disease. Gen Dent. Jan-Feb;60(1):e1-5. | Unable to access |
| 2012 | 219 | Katagiri S, Nagasawa T, Kobayashi H, Takamatsu H, Bharti P, Izumiyama H, Uchimura I, Tagami T, Suzuki T, Nanbara H, Taniguchi Y, Hayakumo S, Koyanagi T, Himeno-Ando A, Goto M, Kajio H, Takahashi Y, Izumi Y, Noda M. Improvement of glycemic control after periodontal treatment by resolving gingival inflammation in type 2 diabetic patients with periodontal disease. J Diabetes Investig. 2012 Aug 20;3(4):402-9. doi: 10.1111/j.2040-1124.2012.00209.x. PMID: 24843597; PMCID: PMC4019262. | Patients with systemic diseases |
| 2012 | 220 | Maruyama T, Yamanaka R, Yokoi A, Ekuni D, Tomofuji T, Mizukawa N, Onoda T, Eguchi M, Morita M. Relationship between serum albumin concentration and periodontal condition in patients with head and neck cancer. J Periodontol. 2012 Sep;83(9):1110-5. | Patients with systemic diseases |
| 2012 | 221 | Romagna C, Dufour L, Troisgros O, Lorgis L, Richard C, Buffet P, Soulat G, Casillas JM, Rioufol G, Touzery C, Zeller M, Laurent Y, Cottin Y. Periodontal disease: a new factor associated with the presence of multiple complex coronary lesions. J Clin Periodontol. 2012 Jan;39(1):38-44. doi: 10.1111/j.1600-051X.2011.01802.x. Epub 2011 Oct 18. PMID: 22092604. | Patients with systemic diseases |
| 2012 | 222 | Savioli C, Ribeiro AC, Fabri GM, Calich AL, Carvalho J, Silva CA, Viana VS, Bonfá E, Siqueira JT. Persistent periodontal disease hampers anti-tumor necrosis factor treatment response in rheumatoid arthritis. J Clin Rheumatol. 2012 Jun;18(4):180-4. doi: 10.1097/RHU.0b013e31825828be. PMID: 22647860. | Patients with systemic diseases |
| 2012 | 223 | Koromantzos PA, Makrilakis K, Dereka X, Offenbacher S, Katsilambros N, Vrotsos IA, Madianos PN. Effect of non-surgical periodontal therapy on C-reactive protein, oxidative stress, and matrix metalloproteinase (MMP)-9 and MMP-2 levels in patients with type 2 diabetes: a randomized controlled study. J Periodontol. 2012 Jan;83(1):3-10. doi: 10.1902/jop.2011.110148. Epub 2011 May 31. PMID: 21627458. | Patients with systemic diseases |
| 2012 | 224 | Lin SJ, Tu YK, Tsai SC, Lai SM, Lu HK. Non-surgical periodontal therapy with and without subgingival minocycline administration in patients with poorly controlled type II diabetes: a randomized controlled clinical trial. Clin Oral Investig. 2012 Apr;16(2):599-609. doi: 10.1007/s00784-011-0535-x. Epub 2011 Mar 18. PMID: 21416238. | Patients with systemic diseases |
| 2012 | 225 | Miyashita H, Honda T, Maekawa T, Takahashi N, Aoki Y, Nakajima T, Tabeta K, Yamazaki K. Relationship between serum antibody titres to Porphyromonas gingivalis and hs-CRP levels as inflammatory markers of periodontitis. Arch Oral Biol. 2012 Jun;57(6):820-9. doi: 10.1016/j.archoralbio.2011.11.008. Epub 2011 Dec 14. PMID: 22172404. | Unsuitable periodontal case definition |
| 2012 | 226 | Chen L, Luo G, Xuan D, Wei B, Liu F, Li J, Zhang J. Effects of non-surgical periodontal treatment on clinical response, serum inflammatory parameters, and metabolic control in patients with type 2 diabetes: a randomized study. J Periodontol. 2012 Apr;83(4):435-43. doi: 10.1902/jop.2011.110327. Epub 2011 Aug 22. PMID: 21859323. | Patients with systemic diseases |
| 2012 | 227 | López NJ, Quintero A, Casanova PA, Ibieta CI, Baelum V, López R. Effects of periodontal therapy on systemic markers of inflammation in patients with metabolic syndrome: a controlled clinical trial. J Periodontol. 2012 Mar;83(3):267-78. doi: 10.1902/jop.2011.110227. Epub 2011 Jul 12. PMID: 21749167. | Patients with systemic diseases |
| 2012 | 228 | Auyeung L, Wang PW, Lin RT, Hsieh CJ, Lee PY, Zhuang RY, Chang HW. Evaluation of periodontal status and effectiveness of non-surgical treatment in patients with type 2 diabetes mellitus in Taiwan for a 1-year period. J Periodontol. 2012 May;83(5):621-8. doi: 10.1902/jop.2011.110133. Epub 2011 Jun 21. PMID: 21692625. | Patients with systemic diseases |
| 2012 | 229 | Bretz WA. Low-dose doxycycline plus additional therapies may lower systemic inflammation in postmenopausal women with periodontitis. J Evid Based Dent Pract. 2012 Sep;12(3 Suppl):67-8. doi: 10.1016/S1532-3382(12)70016-7. PMID: 23253834. | Postmenopausal women |
| 2012 | 230 | Bokhari SA, Khan AA, Butt AK, Azhar M, Hanif M, Izhar M, Tatakis DN. Non-surgical periodontal therapy reduces coronary heart disease risk markers: a randomized controlled trial. J Clin Periodontol. 2012 Nov;39(11):1065-74. doi: 10.1111/j.1600-051X.2012.01942.x. Epub 2012 Sep 11. PMID: 22966824. | Patients with systemic diseases |
| 2012 | 231 | Dodwad V, Ahuja S, Kukreja BJ. Effect of locally delivered tetracycline hydrochloride as an adjunct to scaling and root planing on Hba1c, C-reactive protein, and lipid profile in type 2 diabetes: A clinico-biochemical study. Contemp Clin Dent. 2012 Apr;3(2):150-4. doi: 10.4103/0976-237X.96816. PMID: 22919212; PMCID: PMC3425095. | Patients with systemic diseases |
| 2012 | 232 | Rastogi P, Singhal R, Sethi A, Agarwal A, Singh VK, Sethi R. Assessment of the effect of periodontal treatment in patients with coronary artery disease : A pilot survey. J Cardiovasc Dis Res. 2012 Apr;3(2):124-7. doi: 10.4103/0975-3583.95366. PMID: 22629030; PMCID: PMC3354455. | Unsuitable periodontal case definition |
| 2012 | 233 | Al-Zahrani MS, Alghamdi HS. Effect of periodontal treatment on serum C-reactive protein level in obese and normal-weight women affected with chronic periodontitis. Saudi Med J. 2012 Mar;33(3):309-14. PMID: 22426913. | Unable to access |
| 2012 | 234 | Siribamrungwong M, Puangpanngam K. Treatment of periodontal diseases reduces chronic systemic inflammation in maintenance hemodialysis patients. Ren Fail. 2012;34(2):171-5. doi: 10.3109/0886022X.2011.643351. Epub 2012 Jan 9. PMID: 22229644. | Patients with systemic diseases |
| 2011 | 235 | Allen EM, Matthews JB, O' Halloran DJ, Griffiths HR, Chapple IL. Oxidative and inflammatory status in Type 2 diabetes patients with periodontitis. J Clin Periodontol. 2011 Oct;38(10):894-901. doi: 10.1111/j.1600-051X.2011.01764.x. Epub 2011 Aug 24. PMID: 21883360. | Patients with systemic diseases |
| 2011 | 236 | Ardalan MR, Ghabili K, Pourabbas R, Shoja MM. A causative link between periodontal disease and glomerulonephritis: a preliminary study. Ther Clin Risk Manag. 2011;7:93-8. doi: 10.2147/TCRM.S14106. Epub 2011 Mar 8. PMID: 21445283; PMCID: PMC3061848. | Patients with systemic diseases |
| 2011 | 237 | Aspriello SD, Zizzi A, Tirabassi G, Buldreghini E, Biscotti T, Faloia E, Stramazzotti D, Boscaro M, Piemontese M. Diabetes mellitus-associated periodontitis: differences between type 1 and type 2 diabetes mellitus. J Periodontal Res. 2011 Apr;46(2):164-9. doi: 10.1111/j.1600-0765.2010.01324.x. Epub 2010 Nov 26. PMID: 21108647. | Patients with systemic diseases |
| 2011 | 238 | Berent R, Auer J, Schmid P, Krennmair G, Crouse SF, Green JS, Sinzinger H, von Duvillard SP. Periodontal and coronary heart disease in patients undergoing coronary angiography. Metabolism. 2011 Jan;60(1):127-33. doi: 10.1016/j.metabol.2009.12.016. Epub 2010 Jan 22. PMID: 20096894. | Patients with systemic diseases |
| 2011 | 239 | Fernandez-Botran R, Miller JJ, Burns VE, Newton TL. Correlations among inflammatory markers in plasma, saliva and oral mucosal transudate in post-menopausal women with past intimate partner violence. Brain Behav Immun. 2011 Feb;25(2):314-21. doi: 10.1016/j.bbi.2010.09.023. Epub 2010 Oct 1. PMID: 20888902; PMCID: PMC3025073. | Patients with systemic diseases |
| 2011 | 240 | Gomes-Filho IS, Freitas Coelho JM, da Cruz SS, Passos JS, Teixeira de Freitas CO, Aragão Farias NS, Amorim da Silva R, Silva Pereira MN, Lima TL, Barreto ML. Chronic periodontitis and C-reactive protein levels. J Periodontol. 2011 Jul;82(7):969-78. | Patients with systemic diseases |
| 2011 | 241 | Haba D, Teslaru S, Ungureanu D, Hodorog D, Alecu C, Benghiac AG, Zetu L, Ancuţa C, Ancuţa E, Nemţoi A, Iordache C. Evaluation of serum and gingival crevicular fluid C-reactive protein and IL-6 levels in patients with periodontitis and transient ischemic attacks. Rom J Morphol Embryol. 2011;52(4):1243-7. | Patients with systemic diseases |
| 2011 | 242 | Ioannidou E, Swede H, Dongari-Bagtzoglou A. Periodontitis predicts elevated C-reactive protein levels in chronic kidney disease. J Dent Res. 2011 Dec;90(12):1411-5. | Patients with systemic diseases |
| 2011 | 243 | Kodovazenitis G, Pitsavos C, Papadimitriou L, Deliargyris EN, Vrotsos I, Stefanadis C, Madianos PN. Periodontal disease is associated with higher levels of C-reactive protein in non-diabetic, non-smoking acute myocardial infarction patients. J Dent. 2011 Dec;39(12):849-54. | Patients with systemic diseases |
| 2011 | 244 | Meisel, P, Kohlmann T, Wallaschofski H, Kroemer HK,Kocher T (2011). Cholesterol, C-Reactive Protein, and Periodontitis: HMG-CoA-Reductase Inhibitors (Statins) as Effect Modifiers. International Scholarly Research Network. 2011, 125168. https://doi.org/10.5402/2011/125168 | No case group |
| 2011 | 245 | Motta AC, Furini RB, Simão JC, Vieira MB, Ferreira MA, Komesu MC, Foss NT. Could leprosy reaction episodes be exacerbated by oral infections? Rev Soc Bras Med Trop. 2011 Oct;44(5):633-5. doi: 10.1590/s0037-86822011000500022. PMID: 22031082. | Patients with systemic diseases |
| 2011 | 246 | Pejcic, A., Kesic, L., & Milasin, J. (2011). Association between Periodontopathogens and CRP Levels in Patients with Periodontitis in Serbia. Journal of dental research, dental clinics, dental prospects, 5(1), 10–16. doi:10.5681/joddd.2011.003 | Data previously published |
| 2011 | 247 | Straka M, Kazar J, Pijak MR, Gasparovic J, Wsolova L, Mongiellova V. The importance of the presence of aggregatibacter actinomycetemcomitans in sulcus gingivalis of patients with cardiovascular diseases. Med Sci Monit. 2011 Nov;17(11):CR646-649. doi: 10.12659/msm.882050. PMID: 22037744; PMCID: PMC3539491. | Patients with systemic diseases |
| 2011 | 248 | Sezer U, Erciyas K, Pehlivan Y, Ustün K, Tarakçioğlu M, Senyurt SZ, Onat AM. Serum cytokine levels and periodontal parameters in ankylosing spondylitis. J Periodontal Res. 2012 Jun;47(3):396-401. doi: 10.1111/j.1600-0765.2011.01448.x. Epub 2011 Nov 29. PMID: 22126620. | Patients with systemic diseases |
| 2011 | 249 | Tabeta K, Tanabe N, Yonezawa D, Miyashita H, Maekawa T, Takahashi N, Okui T, Nakajima T, Yamazaki K. Elevated antibody titers to Porphyromonas gingivalis as a possible predictor of ischemic vascular disease - results from the Tokamachi-Nakasato cohort study. J Atheroscler Thromb. 2011;18(9):808-17. doi: 10.5551/jat.6957. Epub 2011 Jun 13. PMID: 21670558. | Patients with systemic diseases |
| 2011 | 250 | Tsioufis C, Thomopoulos C, Soldatos N, Kasiakogias A, Andrikou I, Kordalis A, Toutouzas K, Giamarelos G, Tousoulis D, Kallikazaros I, Stefanadis C. Periodontal disease severity and urinary albumin excretion in middle-aged hypertensive patients. Am J Cardiol. 2011 Jan;107(1):52-8. | Patients with systemic diseases |
| 2011 | 251 | Uriza CL, Arregoces FE, Porras JV, Camargo MB, Morales AR. Ultra-Sensitive C-Reactive Protein (US-CRP) in Patients With Periodontal Disease and Risk of Acute Myocardial Infarction. Cardiol Res. 2011 Feb;2(1):27-35. doi: 10.4021/cr11e. Epub 2011 Jan 20. PMID: 28348657; PMCID: PMC5358126. | Patients with systemic diseases |
| 2011 | 252 | Vieira CL, Cury PR, Miname MH, Martinez LR, Bortolotto LA, Giuliano IB, Santos RD, Caramelli B. Severe periodontitis is associated with diastolic blood pressure elevation in individuals with heterozygous familial hypercholesterolemia: a pilot study. J Periodontol. 2011 May;82(5):683-8. doi: 10.1902/jop.2010.100496. Epub 2010 Nov 8. PMID: 21054230. | Patients with systemic diseases |
| 2011 | 253 | Yetkin Ay, Z., Kırzıoğlu, F. Y., Öztürk Tonguç, M., Sütçü, R., & Kapucuoğlu, N. (2011). The gingiva contains leptin and leptin receptor in health and disease. Odontology, 100(2), 222–231. doi:10.1007/s10266-011-0043-0 | Unclear method of CRP measurement |
| 2011 | 254 | Joseph R, Narayan V, Krishnan R, Melemadathil S. Non-surgical periodontal therapy improves serum levels of C-reactive protein and edematous states in female patients with idiopathic edema. J Periodontol. 2011 Feb;82(2):201-9. doi: 10.1902/jop.2010.100258. Epub 2010 Aug 3. PMID: 20681817. | Patients with systemic diseases |
| 2011 | 255 | Payne JB, Golub LM, Stoner JA, Lee HM, Reinhardt RA, Sorsa T, Slepian MJ. The effect of subantimicrobial-dose-doxycycline periodontal therapy on serum biomarkers of systemic inflammation: a randomized, double-masked, placebo-controlled clinical trial. J Am Dent Assoc. 2011 Mar;142(3):262-73. doi: 10.14219/jada.archive.2011.0165. PMID: 21357860; PMCID: PMC3077029. | Postmenopausal women |
| 2011 | 256 | Li X, Tse HF, Yiu KH, Li LS, Jin L. Effect of periodontal treatment on circulating CD34(+) cells and peripheral vascular endothelial function: a randomized controlled trial. J Clin Periodontol. 2011 Feb;38(2):148-56. doi: 10.1111/j.1600-051X.2010.01651.x. Epub 2010 Dec 6. PMID: 21133981. | No serum CRP values |
| 2011 | 257 | Cengiz Mİ, Yayla N, Cengiz K, Bagci H, Taşkın E. Interaction between periodontal disease and systemic secondary amyloidosis: from inflammation to amyloidosis. J Periodontol. 2011 Apr;82(4):566-74. doi: 10.1902/jop.2010.100439. Epub 2010 Nov 2. PMID: 21043797. | Patients with systemic diseases |
| 2011 | 258 | Passoja A, Knuuttila M, Hiltunen L, Karttunen R, Niemelä O, Raunio T, Vainio O, Hedberg P, Tervonen T. Serum interleukin-6 may modulate periodontal inflammation in type 1 diabetic subjects. J Clin Periodontol. 2011 Aug;38(8):687-93. doi: 10.1111/j.1600-051X.2011.01731.x. Epub 2011 Apr 19. PMID: 21504440. | Patients with systemic diseases |
| 2011 | 259 | Gonzales JR, Harnack L, Schmitt-Corsitto G, Boedeker RH, Chakraborty T, Domann E, Meyle J. A novel approach to the use of subgingival controlled-release chlorhexidine delivery in chronic periodontitis: a randomized clinical trial. J Periodontol. 2011 Aug;82(8):1131-9. doi: 10.1902/jop.2011.100287. Epub 2011 Apr 14. PMID: 21491990. | Data in thresholds |
| 2011 | 260 | Sun WL, Chen LL, Zhang SZ, Wu YM, Ren YZ, Qin GM. Inflammatory cytokines, adiponectin, insulin resistance and metabolic control after periodontal intervention in patients with type 2 diabetes and chronic periodontitis. Intern Med. 2011;50(15):1569-74. doi: 10.2169/internalmedicine.50.5166. Epub 2011 Aug 1. PMID: 21804283. | Patients with systemic diseases |
| 2010 | 261 | Alexander KS, Madden TE, Farrell DH. Association between γ' fibrinogen levels and inflammation. Thromb Haemost. 2011 Apr;105(4):605-9. doi: 10.1160/TH10-09-0626. Epub 2010 Dec 21. PMID: 21174007; PMCID: PMC4110682. | Patients with systemic diseases |
| 2010 | 262 | El Attar MM, Zaghloup MZ, Elmenoufr HS. Role of periodontitis in hospital-acquired pneumonia. East Mediterr Health J. 2010 May;16(5):563-9. PMID: 20799559. | Patients with systemic diseases |
| 2010 | 263 | Kobayashi T, Murasawa A, Komatsu Y, Yokoyama T, Ishida K, Abe A, Yamamoto K, Yoshie H. Serum cytokine and periodontal profiles in relation to disease activity of rheumatoid arthritis in Japanese adults. J Periodontol. 2010 May;81(5):650-7. doi: 10.1902/jop.2010.090688. PMID: 20429644. | Patients with systemic diseases |
| 2010 | 264 | Malali E, Basar I, Emekli-Alturfan E, Elemek E, Oktay S, Ayan F, Emekli N, Noyan U. Levels of C-reactive protein and protein C in periodontitis patients with and without cardiovascular disease. Pathophysiol Haemost Thromb. 2010;37(1):49-54. | Patients with systemic diseases |
| 2010 | 265 | Megson, E., Fitzsimmons, T., Dharmapatni, K., & Mark Bartold, P. (2010). C-reactive protein in gingival crevicular fluid may be indicative of systemic inflammation. Journal of Clinical Periodontology, 37(9), 797–804. doi:10.1111/j.1600-051x.2010.01603.x | No serum CRP values |
| 2010 | 266 | Nagarale G, Ravindra S, Thakur S, Setty S. Efficacy of a chairside diagnostic test kit for estimation of C-reactive protein levels in periodontal disease. J Indian Soc Periodontol. 2010 Oct;14(4):213-6. doi: 10.4103/0972-124X.76919. PMID: 21731244; PMCID: PMC3118069. | Patients with systemic diseases |
| 2010 | 267 | Oktay S, Basar I, Emekli-Alturfan E, Malali E, Elemek E, Ayan F, Koldas L, Noyan U, Emekli N. Serum and saliva sialic acid in periodontitis patients with and without cardiovascular disease. Pathophysiol Haemost Thromb. 2010;37(2-4):67-71. | Patients with systemic diseases |
| 2010 | 268 | Renvert S, Ohlsson O, Pettersson T, Persson GR. Periodontitis: a future risk of acute coronary syndrome? A follow-up study over 3 years. J Periodontol. 2010 Jul;81(7):992-1000. doi: 10.1902/jop.2010.090105. PMID: 20350154. | Patients with systemic diseases |
| 2010 | 269 | Shaqman M, Ioannidou E, Burleson J, Hull D, Dongari-Bagtzoglou A. Periodontitis and inflammatory markers in transplant recipients. J Periodontol. 2010 May;81(5):666-72. | Patients with systemic diseases |
| 2010 | 270 | Souccar NM, Chakhtoura M, Ghafari JG, Abdelnoor AM. Porphyromonas gingivalis in dental plaque and serum C-reactive protein levels in pregnancy. J Infect Dev Ctries. 2010 Jun 30;4(6):362-6. | Patients with systemic diseases |
| 2010 | 271 | Tsioufis C, Thomopoulos C, Soldatos N, Syrseloudis D, Kasiakogias A, Silvestros S, Stefanadi E, Mostratou E, Stefanadis C. The conjoint detrimental effect of chronic periodontal disease and systemic inflammation on asymmetric dimethyl-arginine in untreated hypertensive subjects. Atherosclerosis. 2010 Jan;208(1):258-63. doi: 10.1016/j.atherosclerosis.2009.07.017. Epub 2009 Jul 8. PMID: 19646696. | Patients with systemic diseases |
| 2010 | 272 | Nakajima T, Honda T, Domon H, Okui T, Kajita K, Ito H, Takahashi N, Maekawa T, Tabeta K, Yamazaki K. Periodontitis-associated up-regulation of systemic inflammatory mediator level may increase the risk of coronary heart disease. J Periodontal Res. 2010 Feb;45(1):116-22. doi: 10.1111/j.1600-0765.2009.01209.x. Epub 2009 Jul 8. PMID: 19602107. | Unsuitable periodontal case definition |
| 2010 | 273 | Fentoğlu O, Sözen T, Oz SG, Kale B, Sönmez Y, Tonguç MO, Gürgan CA, Aykaç Y, Kirzioğlu FY. Short-term effects of periodontal therapy as an adjunct to anti-lipemic treatment. Oral Dis. 2010 Oct;16(7):648-54. doi: 10.1111/j.1601-0825.2010.01668.x. PMID: 20412449. | Patients with systemic diseases |
| 2010 | 274 | Correa FO, Gonçalves D, Figueredo CM, Bastos AS, Gustafsson A, Orrico SR. Effect of periodontal treatment on metabolic control, systemic inflammation and cytokines in patients with type 2 diabetes. J Clin Periodontol. 2010 Jan;37(1):53-8. doi: 10.1111/j.1600-051X.2009.01498.x. Epub 2009 Nov 24. PMID: 19968741. | Patients with systemic diseases |
| 2010 | 275 | Sun WL, Chen LL, Zhang SZ, Ren YZ, Qin GM. Changes of adiponectin and inflammatory cytokines after periodontal intervention in type 2 diabetes patients with periodontitis. Arch Oral Biol. 2010 Dec;55(12):970-4. doi: 10.1016/j.archoralbio.2010.08.001. PMID: 20889139. | Patients with systemic diseases |
| 2009 | 276 | Bayraktar G, Kurtulus I, Kazancioglu R, Bayramgurler I, Cintan S, Bural C, Bozfakioglu S, Issever H, Yildiz A. Oral health and inflammation in patients with end-stage renal failure. Perit Dial Int. 2009 Jul-Aug;29(4):472-9. PMID: 19602614. | Patients with systemic diseases |
| 2009 | 277 | Blach A, Franek E, Witula A, Kolonko A, Chudek J, Drugacz J, Wiecek A. The influence of chronic periodontitis on serum TNF-alpha, IL-6 and hs-CRP concentrations, and function of graft and survival of kidney transplant recipients. Clin Transplant. 2009 Mar-Apr;23(2):213-9. | Patients with systemic diseases |
| 2009 | 278 | Biyikoğlu B, Buduneli N, Kardeşler L, Aksu K, Pitkala M, Sorsa T. Gingival crevicular fluid MMP-8 and -13 and TIMP-1 levels in patients with rheumatoid arthritis and inflammatory periodontal disease. J Periodontol. 2009 Aug;80(8):1307-14. | Patients with systemic diseases |
| 2009 | 279 | Cengiz MI, Bagci H, Cengiz S, Yigit S, Cengiz K. Periodontal disease in patients with familial Mediterranean fever: from inflammation to amyloidosis. J Periodontal Res. 2009 Jun;44(3):354-61. doi: 10.1111/j.1600-0765.2008.01115.x. Epub 2008 Oct 7. PMID: 18973533. | Patients with systemic diseases |
| 2009 | 280 | Jared H, Boggess KA, Moss K, Bose C, Auten R, Beck J, Offenbacher S. Fetal exposure to oral pathogens and subsequent risk for neonatal intensive care admission. J Periodontol. 2009 Jun;80(6):878-83. doi: 10.1902/jop.2009.080642. PMID: 19485816. | Patients with systemic diseases |
| 2009 | 281 | Higashi Y, Goto C, Hidaka T, Soga J, Nakamura S, Fujii Y, Hata T, Idei N, Fujimura N, Chayama K, Kihara Y, Taguchi A. Oral infection-inflammatory pathway, periodontitis, is a risk factor for endothelial dysfunction in patients with coronary artery disease. Atherosclerosis. 2009 Oct;206(2):604-10. doi: 10.1016/j.atherosclerosis.2009.03.037. Epub 2009 Apr 5. PMID: 19410250. | Patients with systemic diseases |
| 2009 | 282 | Katagiri S, Nitta H, Nagasawa T, Uchimura I, Izumiyama H, Inagaki K, Kikuchi T, Noguchi T, Kanazawa M, Matsuo A, Chiba H, Nakamura N, Kanamura N, Inoue S, Ishikawa I, Izumi Y. Multi-center intervention study on glycohemoglobin (HbA1c) and serum, high-sensitivity CRP (hs-CRP) after local anti-infectious periodontal treatment in type 2 diabetic patients with periodontal disease. Diabetes Res Clin Pract. 2009 Mar;83(3):308-15. doi: 10.1016/j.diabres.2008.10.016. Epub 2009 Jan 24. PMID: 19168253. | Patients with systemic diseases |
| 2009 | 283 | Mikuls TR, Payne JB, Reinhardt RA, Thiele GM, Maziarz E, Cannella AC, Holers VM, Kuhn KA, O'Dell JR. Antibody responses to Porphyromonas gingivalis (P. gingivalis) in subjects with rheumatoid arthritis and periodontitis. Int Immunopharmacol. 2009 Jan;9(1):38-42. doi: 10.1016/j.intimp.2008.09.008. Epub 2008 Oct 9. PMID: 18848647; PMCID: PMC2748386. | Patients with systemic diseases |
| 2009 | 284 | Sanders, A. E., Slade, G. D., Fitzsimmons, T. R., & Bartold, P. M. (2009). Physical activity, inflammatory biomarkers in gingival crevicular fluid and periodontitis. Journal of Clinical Periodontology, 36(5), 388–395. doi:10.1111/j.1600-051x.2009.01394.x | No mean or/and standard deviation values |
| 2009 | 285 | Sasahara J, Kikuchi A, Takakuwa K, Sugita N, Abiko Y, Yoshie H, Tanaka K. Antibody responses to Porphyromonas gingivalis outer membrane protein in the first trimester. Aust N Z J Obstet Gynaecol. 2009 Apr;49(2):137-41. doi: 10.1111/j.1479-828x.2009.00958.x. PMID: 19441162. | Patients with systemic diseases |
| 2009 | 286 | Sharma A, Ramesh A, Thomas B. Evaluation of plasma C-reactive protein levels in pregnant women with and without periodontal disease: A comparative study. J Indian Soc Periodontol. 2009 Sep;13(3):145-9. doi: 10.4103/0972-124X.60227. PMID: 20379412; PMCID: PMC2848785. | Patients with systemic diseases |
| 2009 | 287 | Willershausen B, Kasaj A, Willershausen I, Zahorka D, Briseño B, Blettner M, Genth-Zotz S, Münzel T. Association between chronic dental infection and acute myocardial infarction. J Endod. 2009 May;35(5):626-30. doi: 10.1016/j.joen.2009.01.012. PMID: 19410072. | Patients with systemic diseases |
| 2009 | 288 | Wohlfeil, M., Wehner, J., Schacher, B., Oremek, G. M., Sauer-Eppel, H., & Eickholz, P. (2009). Degree of gingivitis correlates to systemic inflammation parameters. Clinica Chimica Acta, 401(1-2), 105–109. doi:10.1016/j.cca.2008.11.017 | No case group |
| 2009 | 289 | Yoshii S, Tsuboi S, Morita I, Takami Y, Adachi K, Inukai J, Inagaki K, Mizuno K, Nakagaki H. Temporal association of elevated C-reactive protein and periodontal disease in men. J Periodontol. 2009 May;80(5):734-9. | Patients with systemic diseases |
| 2009 | 290 | Offenbacher S, Beck JD, Moss K, Mendoza L, Paquette DW, Barrow DA, Couper DJ, Stewart DD, Falkner KL, Graham SP, Grossi S, Gunsolley JC, Madden T, Maupome G, Trevisan M, Van Dyke TE, Genco RJ. Results from the Periodontitis and Vascular Events (PAVE) Study: a pilot multicentered, randomized, controlled trial to study effects of periodontal therapy in a secondary prevention model of cardiovascular disease. J Periodontol. 2009 Feb;80(2):190-201. doi: 10.1902/jop.2009.080007. PMID: 19186958; PMCID: PMC2778200. | Data in thresholds |
| 2009 | 291 | Vidal F, Figueredo CM, Cordovil I, Fischer RG. Periodontal therapy reduces plasma levels of interleukin-6, C-reactive protein, and fibrinogen in patients with severe periodontitis and refractory arterial hypertension. J Periodontol. 2009 May;80(5):786-91. doi: 10.1902/jop.2009.080471. PMID: 19405832. | Patients with systemic diseases |
| 2009 | 292 | Michalowicz BS, Novak MJ, Hodges JS, DiAngelis A, Buchanan W, Papapanou PN, Mitchell DA, Ferguson JE, Lupo V, Bofill J, Matseoane S, Steffen M, Ebersole JL. Serum inflammatory mediators in pregnancy: changes after periodontal treatment and association with pregnancy outcomes. J Periodontol. 2009 Nov;80(11):1731-41. doi: 10.1902/jop.2009.090236. PMID: 19905943; PMCID: PMC2922720. | Undefined follow-up period |
| 2009 | 293 | Hussain Bokhari SA, Khan AA, Tatakis DN, Azhar M, Hanif M, Izhar M. Non-surgical periodontal therapy lowers serum inflammatory markers: a pilot study. J Periodontol. 2009 Oct;80(10):1574-80. doi: 10.1902/jop.2009.090001. PMID: 19792845. | Unsuitable periodontal case definition |
| 2008 | 294 | Abou-Raya S, Abou-Raya A, Naim A, Abuelkheir H. Rheumatoid arthritis, periodontal disease and coronary artery disease. Clin Rheumatol. 2008 Apr;27(4):421-7. doi: 10.1007/s10067-007-0714-y. Epub 2007 Aug 29. Erratum in: Clin Rheumatol. 2008 Apr;27(4):551. PMID: 17763921. | Patients with systemic diseases |
| 2008 | 295 | Amabile N, Susini G, Pettenati-Soubayroux I, Bonello L, Gil JM, Arques S, Bonfil JJ, Paganelli F. Severity of periodontal disease correlates to inflammatory systemic status and independently predicts the presence and angiographic extent of stable coronary artery disease. J Intern Med. 2008 Jun;263(6):644-52. doi: 10.1111/j.1365-2796.2007.01916.x. Epub 2008 Jan 16. PMID: 18205762. | Patients with systemic diseases |
| 2008 | 296 | Chitsazi, M. T., Pourabbas, R., Shirmohammadi, A., Ahmadi Zenouz, G., & Vatankhah, A. H. (2008). Association of Periodontal Diseases with Elevation of Serum C-reactive Protein and Body Mass Index. Journal of dental research, dental clinics, dental prospects, 2(1), 9–14. doi:10.5681/joddd.2008.002 | Data in thresholds |
| 2008 | 297 | Horton AL, Boggess KA, Moss KL, Jared HL, Beck J, Offenbacher S. Periodontal disease early in pregnancy is associated with maternal systemic inflammation among African American women. J Periodontol. 2008 Jul;79(7):1127-32. doi: 10.1902/jop.2008.070655. PMID: 18597593; PMCID: PMC4381567. | Patients with systemic diseases |
| 2008 | 298 | Linden, G. J., McClean, K., Young, I., Evans, A., & Kee, F. (2008). Persistently raised C-reactive protein levels are associated with advanced periodontal disease. Journal of Clinical Periodontology, 35(9), 741–747. doi:10.1111/j.1600-051x.2008.01288.x | Data in thresholds |
| 2008 | 299 | Lu, B., Parker, D., & Eaton, C. B. (2008). Relationship of periodontal attachment loss to peripheral vascular disease: An analysis of NHANES 1999–2002 data. Atherosclerosis, 200(1), 199–205. doi:10.1016/j.atherosclerosis.2007.12.037 | No case group |
| 2008 | 300 | Lund Håheim L, Olsen I, Nafstad P, Schwarze P, Rønningen KS. Antibody levels to single bacteria or in combination evaluated against myocardial infarction. J Clin Periodontol. 2008 Jun;35(6):473-8. doi: 10.1111/j.1600-051X.2008.01229.x. Epub 2008 Apr 9. PMID: 18410396. | Patients with systemic diseases |
| 2008 | 301 | Nilsson M, Kopp S. Gingivitis and periodontitis are related to repeated high levels of circulating tumor necrosis factor-alpha in patients with rheumatoid arthritis. J Periodontol. 2008 Sep;79(9):1689-96. | Patients with systemic diseases |
| 2008 | 302 | Ruma M, Boggess K, Moss K, Jared H, Murtha A, Beck J, Offenbacher S. Maternal periodontal disease, systemic inflammation, and risk for preeclampsia. Am J Obstet Gynecol. 2008 Apr;198(4):389.e1-5. | Patients with systemic diseases |
| 2008 | 303 | Sabbah W, Watt RG, Sheiham A, Tsakos G. Effects of allostatic load on the social gradient in ischaemic heart disease and periodontal disease: evidence from the Third National Health and Nutrition Examination Survey. J Epidemiol Community Health. 2008 May;62(5):415-20. doi: 10.1136/jech.2007.064188. PMID: 18413454. | Patients with systemic diseases |
| 2008 | 304 | Makiura N, Ojima M, Kou Y, Furuta N, Okahashi N, Shizukuishi S, Amano A. Relationship of Porphyromonas gingivalis with glycemic level in patients with type 2 diabetes following periodontal treatment. Oral Microbiol Immunol. 2008 Aug;23(4):348-51. doi: 10.1111/j.1399-302X.2007.00426.x. PMID: 18582336. | Patients with systemic diseases |
| 2008 | 305 | Higashi Y, Goto C, Jitsuiki D, Umemura T, Nishioka K, Hidaka T, Takemoto H, Nakamura S, Soga J, Chayama K, Yoshizumi M, Taguchi A. Periodontal infection is associated with endothelial dysfunction in healthy subjects and hypertensive patients. Hypertension. 2008 Feb;51(2):446-53. doi: 10.1161/HYPERTENSIONAHA.107.101535. Epub 2007 Nov 26. PMID: 18039979. | Patients with systemic diseases |
| 2007 | 306 | Blum A, Front E, Peleg A (2007). Periodontal care may improve systemic inflammation. Clinical and Investigative Medicine, 30(3), E114-117. https://doi.org/10.25011/cim.v30i3.1079 | Unclear method of CRP measurement |
| 2007 | 307 | Cengiz MI, Bal S, Gökçay S, Cengiz K. Does periodontal disease reflect atherosclerosis in continuous ambulatory peritoneal dialysis patients? J Periodontol. 2007 Oct;78(10):1926-34. doi: 10.1902/jop.2007.060499. PMID: 18062114. | Patients with systemic diseases |
| 2007 | 308 | Herrera JA, Parra B, Herrera E, Botero JE, Arce RM, Contreras A, López-Jaramillo P. Periodontal disease severity is related to high levels of C-reactive protein in pre-eclampsia. J Hypertens. 2007 Jul;25(7):1459-64. | Patients with systemic diseases |
| 2007 | 309 | Kshirsagar AV, Craig RG, Beck JD, Moss K, Offenbacher S, Kotanko P, Yoshino M, Levin NW, Yip JK, Almas K, Lupovici E, Falk RJ. Severe periodontitis is associated with low serum albumin among patients on maintenance hemodialysis therapy. Clin J Am Soc Nephrol. 2007 Mar;2(2):239-44. doi: 10.2215/CJN.02420706. Epub 2007 Jan 24. PMID: 17699419. | Patients with systemic diseases |
| 2007 | 310 | Lim LP, Tay FB, Sum CF, Thai AC. Relationship between markers of metabolic control and inflammation on severity of periodontal disease in patients with diabetes mellitus. J Clin Periodontol. 2007 Feb;34(2):118-23. doi: 10.1111/j.1600-051X.2006.01032.x. PMID: 17309586. | Patients with systemic diseases |
| 2007 | 311 | Pussinen PJ, Tuomisto K, Jousilahti P, Havulinna AS, Sundvall J, Salomaa V. Endotoxemia, immune response to periodontal pathogens, and systemic inflammation associate with incident cardiovascular disease events. Arterioscler Thromb Vasc Biol. 2007 Jun;27(6):1433-9. doi: 10.1161/ATVBAHA.106.138743. Epub 2007 Mar 15. PMID: 17363692. | Patients with systemic diseases |
| 2007 | 312 | Ziebolz D, Jäger GC, Hornecker E, Mausberg RF. Periodontal findings and blood analysis of blood donors: a pilot study. J Contemp Dent Pract. 2007 Jul 1;8(5):43-50. PMID: 17618329. | No mean or/and standard deviation values |
| 2007 | 313 | Pischon N, Hägewald S, Kunze M, Heng N, Christan C, Kleber BM, Müller C, Bernimoulin JP. Influence of periodontal therapy on the regulation of soluble cell adhesion molecule expression in aggressive periodontitis patients. J Periodontol. 2007 Apr;78(4):683-90. doi: 10.1902/jop.2007.060286. PMID: 17397316. | No serum CRP values |
| 2007 | 314 | Tüter G, Kurtiş B, Serdar M, Aykan T, Okyay K, Yücel A, Toyman U, Pinar S, Cemri M, Cengel A, Walker SG, Golub LM. Effects of scaling and root planing and sub-antimicrobial dose doxycycline on oral and systemic biomarkers of disease in patients with both chronic periodontitis and coronary artery disease. J Clin Periodontol. 2007 Aug;34(8):673-81. doi: 10.1111/j.1600-051X.2007.01104.x. Epub 2007 Jun 21. PMID: 17590156. | Patients with systemic diseases |
| 2007 | 315 | Lalla E, Kaplan S, Yang J, Roth GA, Papapanou PN, Greenberg S. Effects of periodontal therapy on serum C-reactive protein, sE-selectin, and tumor necrosis factor-alpha secretion by peripheral blood-derived macrophages in diabetes. A pilot study. J Periodontal Res. 2007 Jun;42(3):274-82. doi: 10.1111/j.1600-0765.2006.00945.x. PMID: 17451548. | Patients with systemic diseases |
| 2006 | 316 | Briggs JE, McKeown PP, Crawford VL, Woodside JV, Stout RW, Evans A, Linden GJ. Angiographically confirmed coronary heart disease and periodontal disease in middle-aged males. J Periodontol. 2006 Jan;77(1):95-102. doi: 10.1902/jop.2006.77.1.95. PMID: 16579709. | Patients with systemic diseases |
| 2006 | 317 | Chen LP, Chiang CK, Chan CP, Hung KY, Huang CS. Does periodontitis reflect inflammation and malnutrition status in hemodialysis patients? Am J Kidney Dis. 2006 May;47(5):815-22. doi: 10.1053/j.ajkd.2006.01.018. PMID: 16632020. | Patients with systemic diseases |
| 2006 | 318 | Czerniuk MR, Górska R, Filipiak KJ, Opolski G. C-reactive protein in patients with coexistent periodontal disease and acute coronary syndromes. J Clin Periodontol. 2006 Jun;33(6):415-20. doi: 10.1111/j.1600-051X.2006.00931.x. PMID: 16677330. | Patients with systemic diseases |
| 2006 | 319 | Reichert S, Machulla HK, Fuchs C, John V, Schaller HG, Stein J. Is there a relationship between juvenile idiopathic arthritis and periodontitis? J Clin Periodontol. 2006 May;33(5):317-23. doi: 10.1111/j.1600-051X.2006.00909.x. PMID: 16634951. | Patients with systemic diseases |
| 2006 | 320 | Takeda M, Ojima M, Yoshioka H, Inaba H, Kogo M, Shizukuishi S, Nomura M, Amano A. Relationship of serum advanced glycation end products with deterioration of periodontitis in type 2 diabetes patients. J Periodontol. 2006 Jan;77(1):15-20. doi: 10.1902/jop.2006.77.1.15. PMID: 16579698. | Patients with systemic diseases |
| 2006 | 321 | Offenbacher S, Lin D, Strauss R, McKaig R, Irving J, Barros SP, Moss K, Barrow DA, Hefti A, Beck JD. Effects of periodontal therapy during pregnancy on periodontal status, biologic parameters, and pregnancy outcomes: a pilot study. J Periodontol. 2006 Dec;77(12):2011-24. doi: 10.1902/jop.2006.060047. PMID: 17209786. | Undefined follow-up period |
| 2006 | 322 | Chee HK, Lim LP, Tay F, Thai AC, Sum CF. Non-surgical periodontal therapy and serum lipid levels in patients with diabetes mellitus. Ann R Australas Coll Dent Surg. 2006 Sep;18:46. PMID: 17668592. | Patients with systemic diseases |
| 2005 | 323 | Persson GR, Pettersson T, Ohlsson O, Renvert S. High-sensitivity serum C-reactive protein levels in subjects with or without myocardial infarction or periodontitis. J Clin Periodontol. 2005 Mar;32(3):219-24. | Patients with systemic diseases |
| 2005 | 324 | Montebugnoli L, Servidio D, Miaton RA, Prati C, Tricoci P, Melloni C, Melandri G. Periodontal health improves systemic inflammatory and haemostatic status in subjects with coronary heart disease. J Clin Periodontol. 2005 Feb;32(2):188-92. doi: 10.1111/j.1600-051X.2005.00641.x. PMID: 15691350. | Only males included |
| 2005 | 325 | Yamazaki K, Honda T, Oda T, Ueki-Maruyama K, Nakajima T, Yoshie H, Seymour GJ. Effect of periodontal treatment on the C-reactive protein and proinflammatory cytokine levels in Japanese periodontitis patients. J Periodontal Res. 2005 Feb;40(1):53-8. doi: 10.1111/j.1600-0765.2004.00772.x. PMID: 15613080. | Patients with systemic diseases |
| 2005 | 326 | D'Aiuto F, Casas JP, Shah T, Humphries SE, Hingorani AD, Tonetti MS. C-reactive protein (+1444C>T) polymorphism influences CRP response following a moderate inflammatory stimulus. Atherosclerosis. 2005 Apr;179(2):413-7. doi: 10.1016/j.atherosclerosis.2004.10.036. Epub 2004 Dec 18. PMID: 15777561. | Unrelated |
| 2004 | 327 | Joshipura, K. J., Wand, H. C., Merchant, A. T., & Rimm, E. B. (2004). Periodontal Disease and Biomarkers Related to Cardiovascular Disease. Journal of Dental Research, 83(2), 151–155. | Only males included |
| 2004 | 328 | Montebugnoli L, Servidio D, Miaton RA, Prati C, Tricoci P, Melloni C. Poor oral health is associated with coronary heart disease and elevated systemic inflammatory and haemostatic factors. J Clin Periodontol. 2004 Jan;31(1):25-9. doi: 10.1111/j.0303-6979.2004.00432.x. PMID: 15058371. | Patients with systemic diseases |
| 2003 | 329 | Furuichi, Y., Shimotsu, A., Ito, H., Namariyama, Y., Yotsumoto, Y., Hino, Y., … Izumi, Y. (2003). Associations of Periodontal Status with General Health Conditions and Serum Antibody Titers forPorphyromonas gingivalisandActinobacillus actinomycetemcomitans. Journal of Periodontology, 74(10), 1491–1497. | Unclear method of CRP measurement |
| 2003 | 330 | Meurman JH, Janket SJ, Qvarnström M, Nuutinen P. Dental infections and serum inflammatory markers in patients with and without severe heart disease. Oral Surg Oral Med Oral Pathol Oral Radiol Endod. 2003 Dec;96(6):695-700. doi: 10.1016/j.tripleo.2003.08.017. PMID: 14676760. | Patients with systemic diseases |
| 2003 | 331 | Miranda LA, Fischer RG, Sztajnbok FR, Figueredo CM, Gustafsson A. Periodontal conditions in patients with juvenile idiopathic arthritis. J Clin Periodontol. 2003 Nov;30(11):969-74. doi: 10.1034/j.1600-051x.2003.00406.x. PMID: 14761119. | Patients with systemic diseases |
| 2003 | 332 | Saito, T., Murakami, M., Shimazaki, Y., Oobayashi, K., Matsumoto, S., & Koga, T. (2003). Association Between Alveolar Bone Loss and Elevated Serum C-Reactive Protein in Japanese Men. Journal of Periodontology, 74(12), 1741–1746. | Only males included |
| 2003 | 333 | Iwamoto Y, Nishimura F, Soga Y, Takeuchi K, Kurihara M, Takashiba S, Murayama Y. Antimicrobial periodontal treatment decreases serum C-reactive protein, tumor necrosis factor-alpha, but not adiponectin levels in patients with chronic periodontitis. J Periodontol. 2003 Aug;74(8):1231-6. doi: 10.1902/jop.2003.74.8.1231. PMID: 14514239. | Patients with systemic diseases |
| 2002 | 334 | Abou-Raya S, Naeem A, Abou-El KH, El BS. Coronary artery disease and periodontal disease: is there a link? Angiology. 2002 Mar-Apr;53(2):141-8. doi: 10.1177/000331970205300203. PMID: 11952103. | Patients with systemic diseases |
| 2002 | 335 | Beck, J. D., & Offenbacher, S. (2002). Relationships Among Clinical Measures of Periodontal Disease and Their Associations With Systemic Markers. Annals of Periodontology, 7(1), 79–89. | No control group |
| 2002 | 336 | Rahmati MA, Craig RG, Homel P, Kaysen GA, Levin NW. Serum markers of periodontal disease status and inflammation in hemodialysis patients. Am J Kidney Dis. 2002 Nov;40(5):983-9. doi: 10.1053/ajkd.2002.36330. PMID: 12407643. | Patients with systemic diseases |
| 2002 | 337 | Mattila K, Vesanen M, Valtonen V, Nieminen M, Palosuo T, Rasi V, Asikainen S. Effect of treating periodontitis on C-reactive protein levels: a pilot study. BMC Infect Dis. 2002 Dec 10;2:30. doi: 10.1186/1471-2334-2-30. Epub 2002 Dec 10. PMID: 12475397; PMCID: PMC138813. | No mean or/and standard deviation values |
| 2001 | 338 | Mercado FB, Marshall RI, Klestov AC, Bartold PM. Relationship between rheumatoid arthritis and periodontitis. J Periodontol. 2001 Jun;72(6):779-87. doi: 10.1902/jop.2001.72.6.779. PMID: 11453241. | Patients with systemic diseases |
| 2000 | 339 | Loos, B. G., Craandijk, J., Hoek, F. J., Dillen, P. M. E. W., & Velden, U. V. D. (2000). Elevation of Systemic Markers Related to Cardiovascular Diseases in the Peripheral Blood of Periodontitis Patients. Journal of Periodontology, 71(10), 1528–1534. | No mean or/and standard deviation values |
| 2000 | 340 | Slade, G. D., Offenbacher, S., Beck, J. D., Heiss, G., & Pankow, J. S. (2000). Acute-phase Inflammatory Response to Periodontal Disease in the US Population. Journal of Dental Research, 79(1), 49–57. | No case group |
| 1997 | 341 | Ebersole JL, Machen RL, Steffen MJ, Willmann DE. Systemic acute-phase reactants, C-reactive protein and haptoglobin, in adult periodontitis. Clin Exp Immunol. 1997 Feb;107(2):347-52. doi: 10.1111/j.1365-2249.1997.270-ce1162.x. PMID: 9030874; PMCID: PMC1904587. | Unsuitable periodontal treatment |

Supplementary 3 – References of included case control-studies

Altıngöz SM, Kurgan Ş, Önder C, Serdar MA, Ünlütürk U, Uyanık M, Başkal N, Tatakis DN, Günhan M. 2020. Salivary and serum oxidative stress biomarkers and advanced glycation end products in periodontitis patients with or without diabetes: A cross-sectional study. Journal of Periodontology. doi:10.1002/JPER.20-0406.

Amar S, Gokce N, Morgan S, Loukideli M, Van Dyke TE, Vita JA. 2003. Periodontal disease is associated with brachial artery endothelial dysfunction and systemic inflammation. Arteriosclerosis, Thrombosis, and Vascular Biology. 23(7):1245–1249. doi:10.1161/01.ATV.0000078603.90302.4A.

Andrukhov O, Haririan H, Bertl K, Rausch WD, Bantleon HP, Moritz A, Rausch-Fan X. 2013. Nitric oxide production, systemic inflammation and lipid metabolism in periodontitis patients: Possible gender aspect. Journal of Clinical Periodontology. 40(10):916–923. doi:10.1111/jcpe.12145.

Antonoglou GN, Knuuttila M, Niemelä O, Raunio T, Karttunen R, Vainio O, Hedberg P, Ylöstalo P, Tervonen T. 2015. Low serum level of 1,25(OH)2D is associated with chronic periodontitis. Journal of Periodontal Research. 50(2):274–280. doi:10.1111/jre.12207.

Ardila CM, Guzmán IC. 2015. Comparison of serum amyloid a protein and C-reactive protein levels as inflammatory markers in periodontitis. Journal of Periodontal and Implant Science. 45(1):14–22. doi:10.5051/jpis.2015.45.1.14.

Bansal T, Pandey A, Deepa D, Asthana AK. 2014. C-reactive protein (CRP) and its association with periodontal disease: A brief review. Journal of Clinical and Diagnostic Research. 8(7):21–24. doi:10.7860/JCDR/2014/8355.4646.

Bizzarro S, Van Der Velden U, Ten Heggeler JMAG, Leivadaros E, Hoek FJ, Gerdes VEA, Bakker SJL, Gans ROB, Ten Cate H, Loos BG. 2007. Periodontitis is characterized by elevated PAI-1 activity. Journal of Clinical Periodontology. 34(7):574–580. doi:10.1111/j.1600-051X.2007.01095.x.

Buhlin K, Hultin M, Norderyd O, Persson L, Pockley AG, Rabe P, Klinge B, Gustafsson A. 2009. Risk factors for atherosclerosis in cases with severe periodontitis. Journal of Clinical Periodontology. 36(7):541–549. doi:10.1111/j.1600-051X.2009.01430.x.

Cairo F, Nieri M, Gori AM, Tonelli P, Branchi R, Castellani S, Abbate R, Pini-Prato GP. 2010. Markers of systemic inflammation in periodontal patients: chronic versus aggressive periodontitis. An explorative cross-sectional study. European journal of oral implantology. 3(2):147–53.

Chandy S, Joseph K, Sankaranarayanan A, Issac A, Babu G, Wilson B, Joseph J. 2017. Evaluation of c-reactive protein and fibrinogen in patients with chronic and aggressive periodontitis: A clinico-biochemical study. Journal of Clinical and Diagnostic Research. 11(3):ZC41–ZC45. doi:10.7860/JCDR/2017/23100.9552.

Chopra R, Patil S, Kalburgi N, Mathur S. 2012. Association between alveolar bone loss and serum C-reactive protein levels in aggressive and chronic periodontitis patients. Journal of Indian Society of Periodontology. 16(1):28. doi:10.4103/0972-124X.94600.

Craig RG, Yip JK, So MK, Boylan RJ, Socransky SS, Haffajee AD. 2003. Relationship of Destructive Periodontal Disease to the Acute-Phase Response. Journal of Periodontology. 74(7):1007–1016. doi:10.1902/jop.2003.74.7.1007.

D’Aiuto F, Nibali L, Parkar M, Patel K, Suvan J, Donos N. 2010. Oxidative stress, systemic inflammation, and severe periodontitis. Journal of Dental Research. 89(11):1241–1246. doi:10.1177/0022034510375830.

Deliargyris EN, Madianos PN, Kadoma W, Marron I, Smith SC, Beck JD, Offenbacher S. 2004. Periodontal disease in patients with acute myocardial infarction: Prevalence and contribution to elevated C-reactive protein levels. American Heart Journal. 147(6):1005–1009. doi:10.1016/j.ahj.2003.12.022.

Dye BA, Choudhary K, Shea S, Papapanou PN. 2005. Serum antibodies to periodontal pathogens and markers of systemic inflammation. Journal of Clinical Periodontology. 32(12):1189–1199. doi:10.1111/j.1600-051X.2005.00856.x.

Ebersole JL, Machen RL, Steffen MJ, Willmann DE. 1997. Systemic acute-phase reactants, C-reactive protein and haptoglobin, in adult periodontitis. Clinical and Experimental Immunology. 107(2):347–352. doi:10.1111/j.1365-2249.1997.270-ce1162.x.

Fredriksson MI, Figueredo CMS, Gustafsson A, Bergström KG, Åsman BE. 1999. Effect of Periodontitis and Smoking on Blood Leukocytes and Acute-Phase Proteins. Journal of Periodontology. 70(11):1355–1360. doi:10.1902/jop.1999.70.11.1355.

De Freitas COT, Gomes-Filho IS, Naves RC, Da Cruz SS, De Souza Teles Santos CA, Da Silva Barbosa MD. 2011. Effect of non-surgical periodontal therapy on the levels of C-reactive protein: A pilot study. Revista Odonto Ciencia. 26(1):16–21. doi:10.1590/s1980-65232011000100006.

De Freitas Rêgo Bezerra C, Luz de Aquino AR, Costa de Lima K, da Fonte Porto Carreiro A. 2009. Proteína C-reactiva ultrasensible en pacientes con y sin periodontitis crónica severa generalizada. Avances en Periodoncia e Implantología Oral. 21(3):145–156. doi:10.4321/s1699-65852009000300004.

Gaddale R, Mudda JA, Karthikeyan I, Desai SR, Shinde H, Deshpande P. 2016. Changes in cellular and molecular components of peripheral blood in patients with generalized aggressive periodontitis. Journal of investigative and clinical dentistry. 7(1):59–64. doi:10.1111/jicd.12127.

Gani D, Lakshmi D, Krishnan R, Emmadi P. 2009. Evaluation of C-reactive protein and interleukin-6 in the peripheral blood of patients with chronic periodontitis. Journal of Indian Society of Periodontology. 13(2):69. doi:10.4103/0972-124X.55840.

Gani DK, Mallineni SK, Ambalavanan, Ramakrishnan, Deepalakshmi, Emmadi P. 2012. Estimation of the levels of C-reactive protein, interleukin-6, total leukocyte count, and differential count in peripheral blood smear of patients with chronic periodontitis in a South Indian population. West Indian Medical Journal. 61(8):826–831. doi:10.7727/wimj.2011.096.

Glurich I, Grossi S, Albini B, Ho A, Shah R, Zeid M, Baumann H, Genco RJ, De Nardin E. 2002. Systemic inflammation in cardiovascular and periodontal disease: Comparative study. Clinical and Diagnostic Laboratory Immunology. 9(2):425–432. doi:10.1128/CDLI.9.2.425-432.2002.

Goyal L, Bey A, Gupta N, Sharma V. 2014. Comparative evaluation of serum C-reactive protein levels in chronic and aggressive periodontitis patients and association with periodontal disease severity. Contemporary Clinical Dentistry. 5(4):484. doi:10.4103/0976-237X.142816.

Han D-H, Shin H-S, Kim M-S, Paek D, Kim H-D. 2012. Group of Serum Inflammatory Markers and Periodontitis–Metabolic Syndrome Coexistence in Koreans. Journal of Periodontology. 83(5):612–620. doi:10.1902/jop.2011.110304.

Havemose-Poulsen A, Westergaard J, Stoltze K, Skjødt H, Danneskiold-Samsøe B, Locht H, Bendtzen K, Holmstrup P. 2006. Periodontal and Hematological Characteristics Associated With Aggressive Periodontitis, Juvenile Idiopathic Arthritis, and Rheumatoid Arthritis. Journal of Periodontology. 77(2):280–288. doi:10.1902/jop.2006.050051.

Isola G, Alibrandi A, Currò M, Matarese M, Ricca S, Matarese G, Ientile R, Kocher T. 2020. Evaluation of salivary and serum asymmetric dimethylarginine (ADMA) levels in patients with periodontal and cardiovascular disease as subclinical marker of cardiovascular risk. Journal of Periodontology. 91(8):1076–1084. doi:10.1002/JPER.19-0446.

Isola G, Matarese G, Ramaglia L, Pedullà E, Rapisarda E, Iorio-Siciliano V. 2020. Association between periodontitis and glycosylated haemoglobin before diabetes onset: a cross-sectional study. Clinical Oral Investigations. 24(8):2799–2808. doi:10.1007/s00784-019-03143-0.

Isola G, Polizzi A, Alibrandi A, Indelicato F, Ferlito S. 2020. Analysis of Endothelin-1 Concentrations in Individuals with Periodontitis. Scientific Reports. 10(1):1652. doi:10.1038/s41598-020-58585-4.

Isola G, Polizzi A, Muraglie S, Leonardi R, Giudice A Lo. 2019. Assessment of vitamin C and antioxidant profiles in saliva and serum in patients with periodontitis and ischemic heart disease. Nutrients. 11(12). doi:10.3390/nu11122956.

Isola G, Polizzi A, Santonocito S, Alibrandi A, Ferlito S. 2019. Expression of salivary and serum malondialdehyde and lipid profile of patients with periodontitis and coronary heart disease. International Journal of Molecular Sciences. 20(23). doi:10.3390/ijms20236061.

Kalburgi V, Sravya L, Warad S, Vijayalaxmi K, Sejal P, Hazeil D. 2014. Role of systemic markers in periodontal diseases: A possible inflammatory burden and risk factor for cardiovascular diseases? Annals of Medical and Health Sciences Research. 4(3):388. doi:10.4103/2141-9248.133465.

Kalra N, R. Pradeep A, Priyanka N, Kumari M. 2013. Association of stem cell factor and high-sensitivity C reactive protein concentrations in crevicular fluid and serum in patients with chronic periodontitis with and without type 2 diabetes. Journal of Oral Science. 55(1):57–62. doi:10.2334/josnusd.55.57.

Keleş GÇ, Çetinkaya BÖ, Şimşek SB, Köprülü D, Kahraman H. 2007. The role of periodontal disease on acute phase proteins in patients with coronary heart disease and diabetes. Turkish Journal of Medical Sciences. 37(1):39–44.

Kumar KRV, Ranganath V, Naik R, Banu S, Nichani AS. 2014. Assessment of high-sensitivity C-reactive protein and lipid levels in healthy adults and patients with coronary artery disease, with and without periodontitis - a cross-sectional study. Journal of Periodontal Research. 49(6):836–844. doi:10.1111/jre.12172.

Kumari M, Pradeep A, Priyanka N, Kalra N, Naik SB. 2014. Crevicular and serum levels of monocyte chemoattractant protein-4 and high-sensitivity C-reactive protein in periodontal health and disease. Archives of Oral Biology. 59(6):645–653. doi:10.1016/j.archoralbio.2014.03.012.

Leira Y, Ameijeira P, Domínguez C, Leira R, Blanco J. 2018. High serum procalcitonin levels in patients with periodontitis and chronic migraine. Journal of Periodontology. 89(9):1069–1074. doi:10.1002/JPER.17-0603.

Leira Y, Ameijeira P, Domínguez C, López-Arias E, Ávila-Gómez P, Pérez-Mato M, Sobrino T, Campos F, D’Aiuto F, Leira R, et al. 2020. Severe periodontitis is linked with increased peripheral levels of sTWEAK and PTX3 in chronic migraineurs. Clinical Oral Investigations. 24(2):597–606. doi:10.1007/s00784-019-02950-9.

Leira Y, Carballo Á, Orlandi M, Aldrey JM, Pías-Peleteiro JM, Moreno F, Vázquez-Vázquez L, Campos F, D’Aiuto F, Castillo J, et al. 2020. Periodontitis and systemic markers of neurodegeneration: A case–control study. Journal of Clinical Periodontology. 47(5):561–571. doi:10.1111/jcpe.13267.

Leite FRM, Nascimento GG, Peres KG, Demarco FF, Horta BL, Peres MA. 2020. Collider bias in the association of periodontitis and carotid intima‐media thickness. Community Dentistry and Oral Epidemiology. 48(4):264–270. doi:10.1111/cdoe.12525.

Liu J, Wu Y, Ding Y, Meng S, Ge S, Deng H. 2010. Evaluation of serum levels of C-reactive protein and lipid profiles in patients with chronic periodontitis and/or coronary heart disease in an ethnic Han population. Quintessence international (Berlin, Germany : 1985). 41(3):239–47.

Loo WTY, Yue Y, Fan C bin, Bai L jun, Dou Y ding, Wang M, Liang H, Cheung MNB, Chow LWC, Li J le, et al. 2012. Comparing serum levels of cardiac biomarkers in cancer patients receiving chemotherapy and subjects with chronic periodontitis. Journal of translational medicine. 10 Suppl 1(Suppl 1):1–7. doi:10.1186/1479-5876-10-s1-s5.

Maboudi A, Eghbalian-Nouzanizadeh A, Seifi H, Bahar A, Heidari M, Mohammadpour RA, Abediankenari S, Poorbaghi SL, Sepehrimanesh M. 2019. Serum levels of interleukin-23 and 35 in patients with and without type 2 diabetes mellitus and chronic periodontitis. Caspian Journal of Internal Medicine. 10(3):295–302. doi:10.22088/cjim.10.3.295.

Martinez-Herrera M, López-Domènech S, Silvestre FJ, Silvestre-Rangil J, Bañuls C, Victor VM, Rocha M. 2018. Chronic periodontitis impairs polymorphonuclear leucocyte–endothelium cell interactions and oxidative stress in humans. Journal of Clinical Periodontology. 45(12):1429–1439. doi:10.1111/jcpe.13027.

Masi S, Salpea KD, Li K, Parkar M, Nibali L, Donos N, Patel K, Taddei S, Deanfield JE, DAiuto F, et al. 2011. Oxidative stress, chronic inflammation, and telomere length in patients with periodontitis. Free Radical Biology and Medicine. 50(6):730–735. doi:10.1016/j.freeradbiomed.2010.12.031.

Miyashita H, Honda T, Maekawa T, Takahashi N, Aoki Y, Nakajima T, Tabeta K, Yamazaki K. 2012. Relationship between serum antibody titres to Porphyromonas gingivalis and hs-CRP levels as inflammatory markers of periodontitis. Archives of Oral Biology. 57(6):820–829. doi:10.1016/j.archoralbio.2011.11.008.

Miyazawa H, Honda T, Miyauchi S, Domon H, Okui T, Nakajima T, Tabeta K, Yamazaki K. 2012. Increased serum PCSK9 concentrations are associated with periodontal infection but do not correlate with LDL cholesterol concentration. Clinica Chimica Acta. 413(1–2):154–159. doi:10.1016/j.cca.2011.09.023.

Mysak J, Podzimek S, Vasakova J, Mazanek J, Vinsu A, Duskova J. 2017. C-reactive protein in patients with aggressive periodontitis. Journal of Dental Sciences. 12(4):368–374. doi:10.1016/j.jds.2017.04.003.

Nethravathy RR, Alamelu S, Arun K, Kumar T. 2014. Evaluation of circulatory and salivary levels of heat shock protein 60 in periodontal health and disease. Indian Journal of Dental Research. 25(3):300–304. doi:10.4103/0970-9290.138317.

Nicu EA, Laine ML, Morré SA, Van der Velden U, Loos BG. 2009. Soluble CD14 in periodontitis. Innate Immunity. 15(2):121–128. doi:10.1177/1753425908101577.

Noack B, Genco RJ, Trevisan M, Grossi S, Zambon JJ, Nardin E De. 2001. Periodontal Infections Contribute to Elevated Systemic C-Reactive Protein Level. Journal of Periodontology. 72(9):1221–1227. doi:10.1902/jop.2000.72.9.1221.

Pejcic A, Kesic LJ, Milasin J. 2011. C-reactive protein as a systemic marker of inflammation in periodontitis. European Journal of Clinical Microbiology and Infectious Diseases. 30(3):407–414. doi:10.1007/s10096-010-1101-1.

Pitiphat W, Joshipura KJ, Gillman MW, Williams PL, Douglass CW, Rich-Edwards JW. 2008. Maternal periodontitis and adverse pregnancy outcomes. Community Dentistry and Oral Epidemiology. 36(1):3–11. doi:10.1111/j.1600-0528.2006.00363.x.

Podzimek S, Mysak J, Janatova T, Duskova J. 2015. C-reactive protein in peripheral blood of patients with chronic and aggressive periodontitis, gingivitis, and gingival recessions. Mediators of Inflammation. 2015. doi:10.1155/2015/564858.

Popławska-Kita A, Siewko K, Szpak P, Król B, Telejko B, Klimiuk PA, Stokowska W, Górska M, Szelachowska M. 2014. Association between type 1 diabetes and periodontal health. Advances in Medical Sciences. 59(1):126–131. doi:10.1016/j.advms.2014.01.002.

Pradeep A, Priyanka N, Prasad M, Kalra N, Kumari M. 2012. Association of progranulin and high sensitivity CRP concentrations in gingival crevicular fluid and serum in chronic periodontitis subjects with and without obesity. Disease Markers. 33(4):207–213. doi:10.3233/DMA-2012-0926.

Pradeep AR, Kumari M, Kalra N, Priyanka N. 2013. Correlation of MCP-4 and high-sensitivity C-reactive protein as a marker of inflammation in obesity and chronic periodontitis. Cytokine. 61(3):772–777. doi:10.1016/j.cyto.2012.12.022.

Pradeep AR, Manjunath RGS, Kathariya R. 2010. Progressive periodontal disease has a simultaneous incremental elevation of gingival crevicular fluid and serum CRP levels. Journal of investigative and clinical dentistry. 1(2):133–138. doi:10.1111/j.2041-1626.2010.00022.x.

Pradeep AR, Martande SS, Singh SP, Suke DK, Raju AP, Naik SB. 2014. Correlation of human S100A12 (EN-RAGE) and high-sensitivity C-reactive protein as gingival crevicular fluid and serum markers of inflammation in chronic periodontitis and type 2 diabetes. Inflammation Research. 63(4):317–323. doi:10.1007/s00011-013-0703-3.

Priyanka N, Kumari M, Kalra N, Arjun P, Naik SB, Pradeep AR. 2013. Crevicular Fluid and Serum Concentrations of Progranulin and High Sensitivity CRP in Chronic Periodontitis and Type 2 Diabetes. Disease Markers. 35:389–394. doi:10.1155/2013/803240.

Rai B, Kaur J, Anand SC. 2012. Possible relationship between periodontitis and dementia in a North Indian old age population: a pilot study. Gerodontology. 29(2):e200–e205. doi:10.1111/j.1741-2358.2010.00441.x.

Ramich T, Asendorf A, Nickles K, Oremek GM, Schubert R, Nibali L, Wohlfeil M, Eickholz P. 2018. Inflammatory serum markers up to 5 years after comprehensive periodontal therapy of aggressive and chronic periodontitis. Clinical Oral Investigations. 22(9):3079–3089. doi:10.1007/s00784-018-2398-x.

Salzberg TN, Overstreet BT, Rogers JD, Califano J V., Best AM, Schenkein HA. 2006. C-Reactive Protein Levels in Patients With Aggressive Periodontitis. Journal of Periodontology. 77(6):933–939. doi:10.1902/jop.2006.050165.

Sharma A, Astekar M, Metgud R, Soni A, Verma M, Patel S. 2014. A study of C-reactive protein, lipid metabolism and peripheral blood to identify a link between periodontitis and cardiovascular disease. Biotechnic and Histochemistry. 89(8):577–582. doi:10.3109/10520295.2014.918280.

Shi D, Liu YY, Li W, Zhang X, Sun XJ, Xu L, Zhang L, Chen Z Bin, Meng HX. 2015. Association between plasma leptin level and systemic inflammatory markers in patients with aggressive periodontitis. Chinese Medical Journal. 128(4):528–532. doi:10.4103/0366-6999.151110.

Sun XJ, Meng HX, Shi D, Xu L, Zhang L, Chen ZB, Feng XH, Lu RF, Ren XY. 2009. Elevation of C-reactive protein and interleukin-6 in plasma of patients with aggressive periodontitis. Journal of Periodontal Research. 44(3):311–316. doi:10.1111/j.1600-0765.2008.01131.x.

Tang K, Lin M, Wu Y, Yan F. 2011. Alterations of serum lipid and inflammatory cytokine profiles in patients with coronary heart disease and chronic periodontitis: A pilot study. Journal of International Medical Research. 39(1):238–248. doi:10.1177/147323001103900126.

Tapashetti RP, Guvva S, Patil SR, Sharma S, Pushpalatha HM. 2014. C-reactive Protein as Predict of Increased Carotid Intima Media Thickness in Patients with Chronic Periodontitis. Journal of international oral health : JIOH. 6(4):47–52.

Temelli B, Yetkin Ay Z, Savas HB, Aksoy F, Kumbul Doguç D, Uskun E, Varol E. 2018. Circulation levels of acute phase proteins pentraxin 3 and serum amyloid A in atherosclerosis have correlations with periodontal inflamed surface area. Journal of Applied Oral Science. 26:1–9. doi:10.1590/1678-7757-2017-0322.

Thakare KS, Deo V, Bhongade ML. 2010. Evaluation of the C-reactive protein serum levels in periodontitis patients with or without atherosclerosis. Indian Journal of Dental Research. 21(3):326. doi:10.4103/0970-9290.70787.

Tian Y, Li J le, Hao L, Yue Y, Wang M, Loo WTY, Cheung MNB, Chow LWC, Liu Q, Yip AYS, et al. 2013. Association of cytokines, high sensitive C-reactive protein, VEGF and beta-defensin-1 gene polymorphisms and their protein expressions with chronic periodontitis in the chinese population. International Journal of Biological Markers. 28(1):100–107. doi:10.5301/jbm.5000010.

Tüter G, Kurtis B, Serdar M. 2007. Evaluation of Gingival Crevicular Fluid and Serum Levels of High-Sensitivity C-Reactive Protein in Chronic Periodontitis Patients With or Without Coronary Artery Disease. Journal of Periodontology. 78(12):2319–2324. doi:10.1902/jop.2007.070150.

Wohlfeil M, Scharf S, Siegelin Y, Schacher B, Oremek GM, Sauer-Eppel H, Schubert R, Eickholz P. 2012. Increased systemic elastase and C-reactive protein in aggressive periodontitis (CLOI-D-00160R2). Clinical Oral Investigations. 16(4):1199–1207. doi:10.1007/s00784-011-0627-7.

Yamazaki K, Honda T, Domon H, Okui T, Kajita K, Amanuma R, Kudoh C, Takashiba S, Kokeguchi S, Nishimura F, et al. 2007. Relationship of periodontal infection to serum antibody levels to periodontopathic bacteria and inflammatory markers in periodontitis patients with coronary heart disease. Clinical & Experimental Immunology. 149(3):445–452. doi:10.1111/j.1365-2249.2007.03450.x.

Yamazaki K, Honda T, Oda T, Ueki-Maruyama K, Nakajima T, Yoshie H, Seymour GJ. 2005. Effect of periodontal treatment on the C-reactive protein and proinflammatory cytokine levels in Japanese periodontitis patients. Journal of Periodontal Research. 40(1):53–58. doi:10.1111/j.1600-0765.2004.00772.x.

Yang TH, Masumi SI, Weng SP, Chen HW, Chuang HC, Chuang KJ. 2015. Personal exposure to particulate matter and inflammation among patients with periodontal disease. Science of the Total Environment. 502:585–589. doi:10.1016/j.scitotenv.2014.09.081.

Yetkin Ay Z, Kırzıoğlu FY, Öztürk Tonguç M, Sütçü R, Kapucuoğlu N. 2012. The gingiva contains leptin and leptin receptor in health and disease. Odontology. 100(2):222–231. doi:10.1007/s10266-011-0043-0.

Supplementary 4 – References of included intervention studies

Acharya A, Bhavsar N, Jadav B, Parikh H. 2010. Cardioprotective effect of periodontal therapy in metabolic syndrome: A pilot study in indian subjects. Metabolic Syndrome and Related Disorders. 8(4):335–341. doi:10.1089/met.2010.0002.

Almaghlouth AA, Cionca N, Cancela JA, Décaillet F, Courvoisier DS, Giannopoulou C, Mombelli A. 2014. Effect of periodontal treatment on peak serum levels of inflammatory markers. Clinical Oral Investigations. 18(9):2113–2121. doi:10.1007/s00784-014-1187-4.

Altay U, Gürgan CA, Ağbaht K. 2013. Changes in Inflammatory and Metabolic Parameters After Periodontal Treatment in Patients With and Without Obesity. Journal of Periodontology. 84(1):13–23. doi:10.1902/jop.2012.110646.

Alyousef AA, Divakar DD, Muzaheed. 2017. Chemically modified tetracyclines an emerging host modulator in chronic periodontitis patients: A randomized, double-blind, placebo-controlled, clinical trial. Microbial Pathogenesis. 110:279–284. doi:10.1016/j.micpath.2017.07.002.

Behle JH, Sedaghatfar MH, Demmer RT, Wolf DL, Celenti R, Kebschull M, Belusko PB, Herrera-Abreu M, Lalla E, Papapanou PN. 2009. Heterogeneity of systemic inflammatory responses to periodontal therapy. Journal of Clinical Periodontology. 36(4):287–294. doi:10.1111/j.1600-051X.2009.01382.x.

Bozoglan A, Ertugrul AS, Taspınar M, Yuzbasioglu B. 2017. Determining the relationship between atherosclerosis and periodontopathogenic microorganisms in chronic periodontitis patients. Acta Odontologica Scandinavica. 75(4):233–242. doi:10.1080/00016357.2017.1280739.

Caribé PMV, Villar CC, Romito GA, Pacanaro AP, Strunz CMC, Takada JY, Cesar LAM, Mansur A de P. 2020. Influence of the treatment of periodontal disease in serum concentration of sirtuin 1 and mannose-binding lectin. Journal of Periodontology. 91(7):900–905. doi:10.1002/JPER.19-0236.

Caúla AL, Lira-Junior R, Tinoco EMB, Fischer RG. 2014. The effect of periodontal therapy on cardiovascular risk markers: A 6-month randomized clinical trial. Journal of Clinical Periodontology. 41(9):875–882. doi:10.1111/jcpe.12290.

D’Aiuto F, Nibali L, Mohamed-Ali V, Vallance P, Tonetti MS. 2004. Periodontal therapy: A novel non-drug-induced experimental model to study human inflammation. Journal of Periodontal Research. 39(5):294–299. doi:10.1111/j.1600-0765.2004.00741.x.

D’Aiuto F, Nibali L, Parkar M, Suvan J, Tonetti MS. 2005. Short-term effects of intensive periodontal therapy on serum inflammatory markers and cholesterol. Journal of Dental Research. 84(3):269–273. doi:10.1177/154405910508400312.

D’Aiuto F, Parkar M, Andreou G, Suvan J, Brett PM, Ready D, Tonetti MS. 2004. Periodontitis and systemic inflammation: Control of the local infection is associated with a reduction in serum inflammatory markers. Journal of Dental Research. 83(2):156–160. doi:10.1177/154405910408300214.

D’Aiuto F, Parkar M, Nibali L, Suvan J, Lessem J, Tonetti MS. 2006. Periodontal infections cause changes in traditional and novel cardiovascular risk factors: Results from a randomized controlled clinical trial. American Heart Journal. 151(5):977–984. doi:10.1016/j.ahj.2005.06.018.

Deore GD, Gurav AN, Patil R, Shete AR, NaikTari RS, Inamdar SP. 2014. Omega 3 fatty acids as a host modulator in chronic periodontitis patients: a randomised, double-blind, palcebo-controlled, clinical trial. Journal of Periodontal & Implant Science. 44(1):25. doi:10.5051/jpis.2014.44.1.25.

Deore GD, Gurav AN, Patil R, Shete AR, Naiktari RS, Inamdar SP. 2014. Herbal anti-inflammatory immunomodulators as host modulators in chronic periodontitis patients: a randomised, double-blind, placebo-controlled, clinical trial. Journal of Periodontal & Implant Science. 44(2):71. doi:10.5051/jpis.2014.44.2.71.

Duzagac E, Cifcibasi E, Erdem MG, Karabey V, Kasali K, Badur S, Cintan S. 2016. Is obesity associated with healing after non-surgical periodontal therapy? A local vs. systemic evaluation. Journal of Periodontal Research. 51(5):604–612. doi:10.1111/jre.12340.

Eickholz P, Siegelin Y, Scharf S, Schacher B, Oremek GM, Sauer-Eppel H, Schubert R, Wohlfeil M. 2013. Non-surgical periodontal therapy decreases serum elastase levels in aggressive but not in chronic periodontitis. Journal of Clinical Periodontology. 40(4):327–333. doi:10.1111/jcpe.12076.

Elter JR, Hinderliter AL, Offenbacher S, Beck JD, Caughey M, Brodala N, Madianos PN. 2006. The effects of periodontal therapy on vascular endothelial function: A pilot trial. American Heart Journal. 151(1):47.e1-47.e6. doi:10.1016/j.ahj.2005.10.002.

Ertugrul A, Bozoglan A, Taspınar M. 2017. The effect of nonsurgical periodontal treatment on serum and gingival crevicular fluid markers in patients with atherosclerosis. Nigerian Journal of Clinical Practice. 20(3):361. doi:10.4103/1119-3077.181369.

Fentoğlu Ö, Kirzioğlu FY, Bulut MT, Kurgan Ş, Koçak H, Sütcü R, Köroğlu BK, Günhan M. 2015. Serum Lp-PLA2: As a novel viewpoint in periodontal treatment of hyperlipidaemics. Turkish Journal of Medical Sciences. 45(3):619–626. doi:10.3906/sag-1406-75.

George A, Janam P. 2013. The short-term effects of non-surgical periodontal therapy on the circulating levels of interleukin-6 and C-reactive protein in patients with chronic periodontitis. Journal of Indian Society of Periodontology. 17(1):36. doi:10.4103/0972-124X.107472.

Graziani F, Cei S, La Ferla F, Vano M, Gabriele M, Tonetti M. 2010. Effects of non-surgical periodontal therapy on the glomerular filtration rate of the kidney: an exploratory trial. Journal of Clinical Periodontology. 37(7):638–643. doi:10.1111/j.1600-051X.2010.01578.x.

Graziani F, Cei S, Orlandi M, Gennai S, Gabriele M, Filice N, Nisi M, D’Aiuto F. 2015. Acute-phase response following full-mouth versus quadrant non-surgical periodontal treatment: A randomized clinical trial. Journal of Clinical Periodontology. 42(9):843–852. doi:10.1111/jcpe.12451.

Graziani F, Cei S, Tonetti M, Paolantonio M, Serio R, Sammartino G, Gabriele M, D’Aiuto F. 2010. Systemic inflammation following non-surgical and surgical periodontal therapy. Journal of Clinical Periodontology. 37(9):848–854. doi:10.1111/j.1600-051X.2010.01585.x.

Graziani F, Gennai S, Petrini M, Bettini L, Tonetti M. 2019. Enamel matrix derivative stabilizes blood clot and improves clinical healing in deep pockets after flapless periodontal therapy: A Randomized Clinical Trial. Journal of Clinical Periodontology. 46(2):231–240. doi:10.1111/jcpe.13074.

Gupta B, Sawhney A, Patil N, Kumar M, Tripathi S, Sinha S, Sharma S, Gupta S. 2015. Effect of surgical periodontal therapy on serum C-reactive protein levels using ELISA in both chronic and aggressive periodontitis patient. Journal of Clinical and Diagnostic Research. 9(10):ZC01–ZC05. doi:10.7860/JCDR/2015/14680.6558.

Al Habashneh R, Alsalman W, Khader Y. 2015. Ozone as an adjunct to conventional nonsurgical therapy in chronic periodontitis: a randomized controlled clinical trial. Journal of Periodontal Research. 50(1):37–43. doi:10.1111/jre.12177.

Ide M, Jagdev D, Coward PY, Crook M, Barclay GR, Wilson RF. 2004. The Short-Term Effects of Treatment of Chronic Periodontitis on Circulating Levels of Endotoxin, C-Reactive Protein, Tumor Necrosis Factor-α, and Interleukin-6. Journal of Periodontology. 75(3):420–428. doi:10.1902/jop.2004.75.3.420.

Ide M, McPartlin D, Coward PY, Crook M, Lumb P, Wilson RF. 2003. Effect of treatment of chronic periodontitis on levels of serum markers of acute-phase inflammatory and vascular responses. Journal of Clinical Periodontology. 30(4):334–340. doi:10.1034/j.1600-051X.2003.00282.x.

Johnston W, Paterson M, Piela K, Davison E, Simpson A, Goulding M, Ramage G, Sherriff A, Culshaw S. 2020. The systemic inflammatory response following hand instrumentation versus ultrasonic instrumentation—A randomized controlled trial. Journal of Clinical Periodontology. 47(9):1087–1097. doi:10.1111/jcpe.13342.

Kamil W, Al Habashneh R, Khader Y, Al Bayati L, Taani D. 2011. Effects of nonsurgical periodontal therapy on C-reactive protein and serum lipids in Jordanian adults with advanced periodontitis. Journal of Periodontal Research. 46(5):616–621. doi:10.1111/j.1600-0765.2011.01380.x.

Kardeşler L, Buduneli N, Çetinkalp Ş, Kinane DF. 2010. Adipokines and Inflammatory Mediators After Initial Periodontal Treatment in Patients With Type 2 Diabetes and Chronic Periodontitis. Journal of Periodontology. 81(1):24–33. doi:10.1902/jop.2009.090267.

Kocher T, Holtfreter B, Petersmann A, Eickholz P, Hoffmann T, Kaner D, Kim TS, Meyle J, Schlagenhauf U, Doering S, et al. 2019. Effect of Periodontal Treatment on HbA1c among Patients with Prediabetes. Journal of Dental Research. 98(2):171–179. doi:10.1177/0022034518804185.

Kurgan, Önder C, Balcı N, Fentoğlu, Eser F, Balseven M, Serdar MA, Tatakis DN, Günhan M. 2017. Gingival crevicular fluid tissue/blood vessel-type plasminogen activator and plasminogen activator inhibitor-2 levels in patients with rheumatoid arthritis: effects of nonsurgical periodontal therapy. Journal of Periodontal Research. 52(3):574–581. doi:10.1111/jre.12425.

Leite SA de M, Casanovas RC, Rodrigues VP, Pereira A de FV, Ferreira TCA, Nascimento FRF do, Nascimento JR do, Gomes-Filho IS, Bastos MG, Pereira ALA. 2019. The effect of nonsurgical periodontal therapy on hepcidin and on inflammatory and iron marker levels. Brazilian oral research. 33:e055. doi:10.1590/1807-3107bor-2019.vol33.0055.

Li X, Tse HF, Yiu KH, Li LSW, Jin L. 2011. Effect of periodontal treatment on circulating CD34 + cells and peripheral vascular endothelial function: A randomized controlled trial. Journal of Clinical Periodontology. 38(2):148–156. doi:10.1111/j.1600-051X.2010.01651.x.

Ling MR, Chapple ILC, Matthews JB. 2016. Neutrophil superoxide release and plasma C-reactive protein levels pre- and post-periodontal therapy. Journal of Clinical Periodontology. 43(8):652–658. doi:10.1111/jcpe.12575.

Lobão WJM, Carvalho RCCD, Leite SAM, Rodrigues VP, Batista JE, Gomes-Filho IS, Pereira ALA. 2019. Relationship between periodontal outcomes and serum biomarkers changes after non-surgical periodontal therapy. Anais da Academia Brasileira de Ciencias. 91(2):e20170652. doi:10.1590/0001-3765201920170652.

Luthra S, Grover HS, Singh A, Lall A, Masamatti SS. 2019. Comparative evaluation of C-reactive protein and complete blood count in chronic periodontitis patients following Phase I therapy: A serological and hematological study. Journal of Indian Society of Periodontology. 23(6):525–533. doi:10.4103/jisp.jisp_639_18.

Mallapragada S, Kasana J, Agrawal P. 2017. Effect of nonsurgical periodontal therapy on serum highly sensitive capsule reactive protein and homocysteine levels in chronic periodontitis: A pilot study. Contemporary Clinical Dentistry. 8(2):279. doi:10.4103/ccd.ccd_140_17.

Marcaccini AM, Meschiari CA, Sorgi CA, Saraiva MCP, de Souza AM, Faccioli LH, Tanus-Santos JE, Novaes AB, Gerlach RF. 2009. Circulating Interleukin-6 and High-Sensitivity C-Reactive Protein Decrease After Periodontal Therapy in Otherwise Healthy Subjects. Journal of Periodontology. 80(4):594–602. doi:10.1902/jop.2009.080561.

Martinez-Herrera M, Silvestre FJ, Silvestre-Rangil J, López-Domènech S, Bañuls C, Rocha M. 2018. Levels of serum retinol-binding protein 4 before and after non-surgical periodontal treatment in lean and obese subjects: An interventional study. Journal of Clinical Periodontology. 45(3):336–344. doi:10.1111/jcpe.12840.

Mohan M, Jhingran R, Bains VK, Gupta V, Madan R, Rizvi I, Mani K. 2014. Impact of scaling and root planing on C-reactive protein levels in gingival crevicular fluid and serum in chronic periodontitis patients with or without diabetes mellitus. Journal of Periodontal and Implant Science. 44(4):158–168. doi:10.5051/jpis.2014.44.4.158.

Morozumi T, Yashima A, Gomi K, Ujiie Y, Izumi Y, Akizuki T, Mizutani K, Takamatsu H, Minabe M, Miyauchi S, et al. 2018. Increased systemic levels of inflammatory mediators following one-stage full-mouth scaling and root planing. Journal of Periodontal Research. 53(4):536–544. doi:10.1111/jre.12543.

Nagarale G, Ravindra S, Thakur S, Setty S. 2010. Efficacy of a chairside diagnostic test kit for estimation of C-reactive protein levels in periodontal disease. Journal of Indian Society of Periodontology. 14(4):213. doi:10.4103/0972-124X.76919.

Patil VA, Desai MH. 2013. Effect of periodontal therapy on serum C-reactive protein levels in patients with gingivitis and chronic periodontitis: A clinicobiochemical study. Journal of Contemporary Dental Practice. 14(2):233–237. doi:10.5005/jp-journals-10024-1305.

Pinho M de N, Oliveira RDR, Novaes AB, Voltarelli JC. 2009. Relationship between periodontitis and rheumatoid arthritis and the effect of non-surgical periodontal treatment. Brazilian Dental Journal. 20(5):355–364. doi:10.1590/S0103-64402009000500001.

Preshaw PM, Taylor JJ, Jaedicke KM, De Jager M, Bikker JW, Selten W, Bissett SM, Whall KM, Merwe R, Areibi A, et al. 2020. Treatment of periodontitis reduces systemic inflammation in type 2 diabetes. Journal of Clinical Periodontology. 47(6):737–746. doi:10.1111/jcpe.13274.

Radafshar G, Shad B, Ariamajd E, Geranmayeh S. 2010. Effect of intensive non-surgical treatment on the level of serum inflammatory markers in advanced periodontitis. Journal of dentistry (Tehran, Iran). 7(1):24–30.

Renvert S, Lindahl C, Roos-Jansåker A-M, Lessem J. 2009. Short-Term Effects of an Anti-Inflammatory Treatment on Clinical Parameters and Serum Levels of C-Reactive Protein and Proinflammatory Cytokines in Subjects With Periodontitis. Journal of Periodontology. 80(6):892–900. doi:10.1902/jop.2009.080552.

Seinost G, Wimmer G, Skerget M, Thaller E, Brodmann M, Gasser R, Bratschko RO, Pilger E. 2005. Periodontal treatment improves endothelial dysfunction in patients with severe periodontitis. American Heart Journal. 149(6):1050–1054. doi:10.1016/j.ahj.2004.09.059.

Shimada Y, Komatsu Y, Ikezawa-Suzuki I, Tai H, Sugita N, Yoshie H. 2010. The Effect of Periodontal Treatment on Serum Leptin, Interleukin-6, and C-Reactive Protein. Journal of Periodontology. 81(8):1118–1123. doi:10.1902/jop.2010.090741.

Siqueira MA de S, Fischer RG, Pereira NR, Martins MA, Moss MB, Mendes-Ribeiro AC, Figueredo CM da S, Brunini TMC. 2013. Effects of non-surgical periodontal treatment on the L-arginine-nitric oxide pathway and oxidative status in platelets. Experimental Biology and Medicine. 238(6):713–722. doi:10.1177/1535370213480690.

de Souza AB, Okawa RTP, Silva CO, Araújo MG. 2017. Short-term changes on C-reactive protein (CRP) levels after non-surgical periodontal treatment in systemically healthy individuals. Clinical Oral Investigations. 21(1):477–484. doi:10.1007/s00784-016-1817-0.

Suvan J, Masi S, Harrington Z, Santini E, Raggi F, D’Aiuto F, Solini A. 2021. Effect of Treatment of Periodontitis on Incretin Axis in Obese and Nonobese Individuals: A Cohort Study. The Journal of Clinical Endocrinology & Metabolism. 106(1):e74–e82. doi:10.1210/clinem/dgaa757.

Tamaki N, Tomofuji T, Ekuni D, Yamanaka R, Morita M. 2011. Periodontal treatment decreases plasma oxidized LDL level and oxidative stress. Clinical Oral Investigations. 15(6):953–958. doi:10.1007/s00784-010-0458-y.

Tasdemir Z, Özsarı Tasdemir F, Gürgan C, Eroglu E, Gunturk I, kocyigit I. 2018. The effect of periodontal disease treatment in patients with continuous ambulatory peritoneal dialysis. International Urology and Nephrology. 50(8):1519–1528. doi:10.1007/s11255-018-1913-y.

Taşdemir Z, Özsarı Taşdemir F, Koçyiğit İ, Yazıcı C, Gürgan CA. 2016. The clinical and systemic effects of periodontal treatment in diabetic and non-diabetic obese patients. Journal of Oral Science. 58(4):523–531. doi:10.2334/josnusd.16-0163.

Taylor B, Tofler G, Morel-Kopp MC, Carey H, Carter T, Elliott M, Dailey C, Villata L, Ward C, Woodward M, et al. 2010. The effect of initial treatment of periodontitis on systemic markers of inflammation and cardiovascular risk: A randomized controlled trial. European Journal of Oral Sciences. 118(4):350–356. doi:10.1111/j.1600-0722.2010.00748.x.

Tonetti MS, D’Aiuto F, Nibali L. 2007. Treatment of periodontitis and endothelial function. The New England Journal of Medicine. 356:911–20. doi:10.1016/s0749-4041(08)70642-0.

Torumtay G, Kırzıoğlu FY, Öztürk Tonguç M, Kale B, Calapoğlu M, Orhan H. 2016. Effects of periodontal treatment on inflammation and oxidative stress markers in patients with metabolic syndrome. Journal of Periodontal Research. 51(4):489–498. doi:10.1111/jre.12328.

Ushida Y, Koshy G, Kawashima Y, Kiji M, Umeda M, Nitta H, Nagasawa T, Ishikawa I, Izumi Y. 2008. Changes in serum interleukin-6, C-reactive protein and thrombomodulin levels under periodontal ultrasonic debridement. Journal of Clinical Periodontology. 35(11):969–975. doi:10.1111/j.1600-051X.2008.01316.x.

Vargas-Villafuerte KR, Dantas FT, Messora MR, Novaes AB, Grisi MF, Taba M, Souza SL, Candido dos Reis FJ, Carrara HHA, Palioto DB. 2016. Preliminary Results of Non-Surgical Periodontal Treatment in Patients With Breast Cancer Undergoing Chemotherapy. Journal of Periodontology. 87(11):1268–1277. doi:10.1902/jop.2016.160101.

Vilela EM, Bastos JA, Fernandes N, Ferreira AP, Chaoubah A, Bastos MG. 2011. Treatment of chronic periodontitis decreases serum prohepcidin levels in patients with chronic kidney disease. Clinics. 66(4):657–662. doi:10.1590/S1807-59322011000400022.

Yashima A, Morozumi T, Yoshie H, Hokari T, Izumi Y, Akizuki T, Mizutani K, Takamatsu H, Minabe M, Miyauchi S, et al. 2019. Biological responses following one-stage full-mouth scaling and root planing with and without azithromycin: Multicenter randomized trial. Journal of Periodontal Research. 54(6):709–719. doi:10.1111/jre.12680.

Žekonis G, Žekonis J, Gleiznys A, Noreikienė V, Balnytė I, Šadzevičienė R, Narbutaitė J. 2016. Effect of supragingival irrigation with aerosolized 0.5% hydrogen peroxide on clinical periodontal parameters, markers of systemic inflammation, and morphology of gingival tissues in patients with periodontitis. Medical Science Monitor. 22:3713–3721. doi:10.12659/MSM.900338.

Zhang J, Zhang AM, Zhang ZM, Jia JL, Sui XX, Yu LR, Liu HT. 2017. Efficacy of combined orthodontic-periodontic treatment for patients with periodontitis and its effect on inflammatory cytokines: A comparative study. American Journal of Orthodontics and Dentofacial Orthopedics. 152(4):494–500. doi:10.1016/j.ajodo.2017.01.028.

Zuza EP, Barroso EM, Fabricio M, Carrareto AL V., Toledo BEC, Pires JR. 2016. Lipid profile and high-sensitivity C-reactive protein levels in obese and non-obese subjects undergoing non-surgical periodontal therapy. Journal of Oral Science. 58(3):423–430. doi:10.2334/josnusd.16-0173.

Supplementary Table 5. Studies characteristics comparing CRP and hs-CRP of otherwise healthy patient with PD, AgP and Non-PD diagnosis.

| Year | **Authors (Year) (Country)** | **Funding** | **N. Total** | **Periodontal condition (n) (Healthy/CP/AgP)** | **Sex (n) (M/F [Healthy]; M/F [CP]; M/F [AgP])** | **Smokers (%) (Healthy/CP/AgP)** | **Age (mean±SD) (Healthy/CP/AgP)** | **PD diagnosis criteria** | **CRP method** |
| --- | --- | --- | --- | --- | --- | --- | --- | --- | --- |
| 2021 | Altıngöz et al. (2021) (Turkey) | Ankara University Scientific Research Projects Office, Ankara, Turkey (12B3334002) | 54 | 28/26/0 | 18/10; 18/8; 0/0 | 17.9% / 23.1 % / - | 44.8±6.5 / 46.1±5.3 / - | AAP 1999 | ELISA |
| 2020 | Leira et al. (2020) (Spain) | Spanish Ministry of Economy, European Commission and UCL | 150 | 75/75/0 | 49/26; 49/26; 0/0 | 9.3% / 14.7% / - | 44.7±12.2 / 44.8±10.3 / - | CDC/AAP 2012 | CLIA |
| 2020 | Isola et al. (2020) (Italy) | Department of General Surgery and Surgical-Medical Specialties, University of Catania | 70 | 35/35/0 | 17/18; 18/17; 0/0 | 11.4% / 14.2% / - | 57.0±0.8 / 56.0±1.8 / - | ≥40% of sites with CAL≥2mm and PPD≥4mm; ≥1 site for each quadrant with ≥2 mm of crestal alveolar RBL; ≥40% sites with BOP | Nephelometry |
| 2020 | Leite et al. (2020) (Danmark) | Coordenação de Aperfeiçoamento de Pessoal de Nível Superior, Grant BEX13810/13-8; Brazilian Public Health Association; Conselho Nacional de Desenvolvimento Científico e Tecnológico, Grant no 229279/2013-9, 403257/2012-3 and 475979/2013-3 | 480 | 305/175/0 | 140/165; 100/75; 0/0 | NR / NR / - | NR / NR / - | CDC/AAP 2012 | ELISA |
| 2020 | Leira et al. (2020) (UK) | Spanish Ministry of Economy and Competitiveness —Institute of Health Carlos III (PI13/02027 and PI15/01578), Spanish Ministry of Economy (RTI2018-102165- B-I00) and European Commission under the PANA project (Call H2020-NMP-2015-two stage, Grant 686009) | 150 | 75/75/0 | 49/26; 49/26; 0/0 | 9.3% / 14.7% / - | 44.7±12.2 / 44.8±10.3 / - | ≥40% of sites with CAL≥2mm and PPD≥4mm; ≥1 site for each quadrant with ≥2 mm of crestal alveolar RBL; ≥40% sites with BOP | ELISA |
| 2020 | Isola G et al. (2020) D (Italy) | Department of University of Catania | 188 | 95/93/0 | 49/46; 45/48; 0/0 | 11.6% / 12.9% / - | 52.2±1.1 / 53.7±1.5 / - | ≥40% of sites with CAL≥2mm and PPD≥4mm; ≥1 site for each quadrant with ≥2 mm of crestal alveolar RBL; ≥40% sites with BOP | ELISA |
| 2020 | Isola G et al. (2020) C (Italy) | Department of the University of Catania | 68 | 34/34/0 | 17/17; 18/16; 0/0 | 11.7% / 11.7% / - | 55.0±1.3 / 54.0±1.3 / - | ≥40% of sites with CAL≥2mm and PPD≥4mm; ≥1 site for each quadrant with ≥2 mm of crestal alveolar RBL; ≥40% sites with BOP | Nephelometry |
| 2019 | Isola a et al. (2019) (Italy) | No | 72 | 36/36/0 | 17/19; 18/18; 0/0 | 8.3% / 8.3% / - | 54±NR / 55.0±NR / - | EFP/AAP 2018 | Nephelometry |
| 2019 | Maboudi et al. (2019) (Iran) | Mazandaran University of Medical Sciences (Grant no 95-01-15-13959) | 36 | 18/18/0 | 5/13; 14/4; 0/0 | 0.0% / 0.0% / - | 27.4±10.2 / 44.8±13.2 / - | At least one site PPD≥3mm and CAL≥ 2 mm | Nephelometry |
| 2019 | Isola b et al. (2019) (Italy) | Department of General Surgery and Surgical-Medical Specialties, University of Catania | 66 | 32/34/0 | 16/16; 16/18; 0/0 | 6.2% / 8.8% / - | 58.0±1.5 / 57.0±1.8 / - | ≥40% of sites with CAL≥2mm and PPD≥4 mm, at least≥3 mm of crestal alveolar RBL, and BoP ≥40% sites | Nephelometry |
| 2018 | Ramich et al. (2018) (Germany) | Yes | 42 | 0/25/17 | 0/0; 15/10; 9/8 | - / 28.0% / 24.0% | - / 58.2±7.2 / 35.2 ± 6.6 | AgP: PPD≥ 3.6 mm at more than 30% of sites. RBL ≥ 50% at a minimum of 2 teeth; ≤ 35 years of age. CP: PPD≥3.6 mm and vertical CAL≥5mm at more than 30% of sites; PPD≥7 mm at a minimum of 4 sites; >35 years of age | Immunoturbidimetry |
| 2018 | Temelli et al. (2018) (Turkey) | Süleyman Demirel University Coordination Unit of Scientific Investigations (Grant no 3849-DU1-14) | 37 | 16/21/0 | 6/10; 14/7; 0/0 | 0.0% / 23.8% / - | 49.0±8.0 / 53.3±7.3 / - | 1999 International Workshop for the Classification of Periodontal Disease and Conditions | Immunoturbidimetry |
| 2018 | Martinez-Herrera et al. (2018) (Spain) | PI16/00301 and PI16/01083 from Carlos III Health and has been co-funded by the European Regional Development Fund | 182 | 37/145/0 | 16/21; 50/95; 0/0 | 0.2 / 25.5% / - | 40.0±11.4 / 44.3±10.7 / - | CDC/AAP 2012 | Nephelometry |
| 2017 | Mysak et al. (2017) (Czech Republic) | Yes | 105 | 60/0/45 | NR/NR; 0/0; NR/NR | 0.0% / - / 0 | 23.4±1.3 / - / 35.2±7.2 | Armitage 1999 | Immunoturbidimetry |
| 2017 | Chandy et al. (2017) (India) | No | 55 | 20/20/15 | NR/NR; NR/NR; NR/NR | 0.0% / 0.0% / 0 | NR / NR / NR | Newman et al., 2011 | ELISA |
| 2017 | Leira et al. (2017) (Spain) | NR | 56 | 37/19/0 | 3/34; 0/19; 0/0 | 13.5% / 26.3% / - | 43.3±9.9 / 48.0±10.9 / - | ≥2 interproximal sites with CAL≥3 mm and ≥2 interproximal sites with PPD≥4 mm (not on the same tooth) or 1 site with PPD≥5 mm | CLIA |
| 2016 | Gaddale et al. (2016) (India) | NR | 120 | 60/0/60 | 27/33; 0/0; 32/28 | 0.0% / - / 0 | 26.9±3.7 / - / 29.9±4.8 | International Workshop for a Classification of Periodontal Diseases and Conditions in 1999 | Immunoturbidimetry |
| 2015 | Yang et al. (2015) (Taiwan) | Yes | 200 | 100/100/0 | 50/50; 50/50; 0/0 | 0.0% / 0.0% / - | 32.4±2.5 / 32.8±2.3 / - | PPD≥5 mm and/or CAL in > 8 teeth | CLIA |
| 2015 | Antonoglou et al. (2015) (Finland) | NR | 85 | 30/55/0 | 11/19; 21/34; 0/0 | 0.0% / 40.0% / - | 41.9±12.7 / 46.3±13.5 / - | ≥40% of sites with CAL≥2mm and PPD≥4 mm, at least≥3 mm of crestal alveolar RBL, and ≥40% sites with BOP | ELISA |
| 2015 | Shi et al. (2015) (China) | Yes | 134 | 44/0/90 | 16/28; 0/0; 33/57 | 0.0% / - / 0 | 25.6±3.8 / - / 26.2±4.9 | International Workshop for a Classifi cation of Periodontal Diseases and Conditions in 1999 | ELISA |
| 2015 | Podzimek et al. (2015) (Czech Republic) | Yes | 145 | 8/111/26 | 5/3; 46/65; 13/13 | 0.0% / 0.0% / 0.0% | 44.3±10.3 / 55.1±11.4 / 37.5±7.4 | AgP: at least 1 tooth with BOP and a PPD>5 mm in all quadrants; CP: at least 1 tooth with BOP and PPD>2 mm in all quadrants | Immunoturbidimetry |
| 2015 | Ardila et al. (2015) (Colombia) | Yes | 110 | 30/80/0 | 12/18; 19/61; 0/0 | 6.9% / 25.0% / - | 46.1±3.4 / 46±9.4 / - | CDC/AAP 2012 | CLIA |
| 2014 | Kumari et al. (2014) (India) | No | 25 | 10/15/0 | 5/5; 7/8; 0/0 | 0.0% / 0.0% / - | 31.8±4.9 / 32.1±4.2 / - | >30% sites had PPD≥5 mm, and CAL≥3 mm, with RBL | Immunoturbidimetry |
| 2014 | Nethravathy et al. (2014) (India) | No | 45 | 22/23/0 | NR/NR; NR/NR; 0/0 | 0.0% / 0.0% / - | NR / NR / - | Armitage 1999 | Immunoturbidimetry |
| 2014 | Tapashetti et al. (2014) (India) | No | 30 | 15/15/0 | 9/6; 9/6; 0/0 | 26.7% / 13.3% / - | 47.7±3.4 / 46.5±2.8 / - | Newman et al., 2011 | Immunoturbidimetry |
| 2014 | Goyal et al. (2014) (India) | No | 75 | 25/25/25 | 15/10; 14/11; 12/13 | 0.0% / 0.0% / 0.0% | 33.5±5.9 / 36.6±8.7 / 24.2±5.0 | CP: PPD≥5mm and/or CAL>30% sites; AgP: <30 years of age, PPD≥5mm and/or CAL on 8 or more teeth | ELISA |
| 2014 | Kalburgi et al. (2014) (India) | No | 30 | 10/20/0 | 6/4; 15/5; 0/0 | 0.0% / 0.0% / - | 34.4±4.6 / 45.8±5.8 / - | PPD>4 mm, CAL>4 mm | Immunoturbidimetry |
| 2014 | Bansal et al. (2014) (India) | No | 40 | 20/20/0 | NR/NR; NR/NR; 0/0 | 0.0% / 0.0% / - | NR / NR / - | ≥8 teeth with PPD ≥ 5 mm, ≥8 teeth with CAL≥5 mm and RBL | Immunoturbidimetry |
| 2014 | Kumar et al. (2014) (India) | Yes | 50 | 25/25/0 | NR/NR; NR/NR; 0/0 | NR / NR / - | NR / NR / - | RBL ≥ 30% of sites affecting at least 50% of the dentition and CAL≥4 mm at ≥6 sites. | Immunoturbidimetry |
| 2014 | Pradeep et al. (2014) (India) | NR | 27 | 10/17/0 | 5/5; 9/8; 0/0 | 0.0% / 0.0% / - | 39.3±3.5 / 38.4±3.2 / - | PPD≥5mm and CAL>3 mm, with RBL | ELISA |
| 2014 | Sharma et al. (2014) (India) | NR | 40 | 20/20/0 | NR/NR; NR/NR; 0/0 | 0.0% / 0.0% / - | NR / NR / - | NHANES 3 | ELISA |
| 2014 | Popławska-Kita et al. (2014) (Poland) | Medical University of Bialystok, Poland | 40 | 34/6/0 | NR/NR; NR/NR; 0/0 | NR / NR / - | 29.4±9.5 / 48.3±2.1 / - | Communitary Periodontal Index | ELISA |
| 2013 | Tian et al. (2013) (China) | Yes | 654 | 532/122/0 | 70/462; 118/4; 0/0 | 0.0% / 0.0% / - | 37.0±10.0 / 54.0±8.0 / - | Armitage 1999 | ELISA |
| 2013 | Priyanka et al. (2013) (India) | NR | 25 | 10/15/0 | 5/5; 8/7; 0/0 | 0.0% / 0.0% / - | 34.2±3.4 / 32.1±3.2 / - | 30% sites with PPD≥5 mm, CAL ≥3 mm, with RBL | Immunoturbidimetry |
| 2013 | Kalra et al. (2013) (India) | NR | 25 | 10/15/0 | 5/5; 8/7; 0/0 | 0.0% / 0.0% / - | 32.7±3.7 / 32.2±3.9 / - | 30% sites with PPD≥5 mm, CAL≥3 mm, with RBL | Immunoturbidimetry |
| 2013 | Andrukhov et al. (2013) (Austria) | Yes | 143 | 54/89/0 | 25/29; 53/36; 0/0 | 0.0% / 0.0% / - | 34.3±1.2 / 42.2±8.4 / - | Armitage 1999 | Immunoturbidimetry |
| 2013 | Pradeep et al. (2013) (India) | Colgate research grant, Colgate Palmolive India limited, Mumbai | 20 | 10/10/0 | 5/5; 5/5; 0/0 | 0.0% / 0.0% / - | 31.4±5.0 / 32.8±4.8 / - | PPD≥5 mm, CAL≥3 mm, with RBL | Immunoturbidimetry |
| 2012 | Wohlfeil et al. (2012) (Germany) | Yes | 90 | 30/31/29 | 14/16; 19/12; 13/16 | 27.0% / 32.0% / 31.0% | 27.5±3.3 / 52.8±7.6 / 31.1±5.7 | AgP: PPD≥3.6 mm at more than 30% of sites, RBL ≥ 50% at a minimum of 2 teeth; ≤ 35 years of age.  CP: PPD≥3.6 mm and vertical CAL≥5mm at more than 30% of sites; PPD≥7 mm at a minimum of 4 sites; >35 years of age | Immunoturbidimetry |
| 2012 | Miyashita et al. (2012) (Japan) | Yes | 89 | 49/40/0 | 19/21; 17/32; 0/0 | 0.0% / 0.0% / - | 48.3±9.9 / 50.8±13.8 / - | Armitage 1999 | Nephelometry |
| 2012 | Miyazawa et al. (2012) (Japan) | Yes | 70 | 30/40/0 | 16/14; 13/27; 0/0 | 0.0% / 17.5% / - | 46.1±6.3 / 52.1±11.9 / - | Armitage 1999 | Nephelometry |
| 2012 | Gani et al. (2012) (India) | NR | 42 | 14/28/0 | NR/NR; NR/NR; 0/0 | 0.0% / 0.0% / - | - / NR / - | PPD≥5mm with RBL | Immunoturbidimetry |
| 2012 | Chopra a et al. (2012) (India) | No | 45 | 15/15/15 | NR/NR; NR/NR; NR/NR | 0.0% / 0.0% / 0 | NR / NR / NR | AgP: PPD≥5 mm and/or CAL on 8 or more teeth, at least 3 of which were not first molars and incisors and < 30 years of age;  CP: PPD≥5 mm and/or CAL in more than 8 teeth and > 30 years of age | Immunoturbidimetry |
| 2012 | Pradeep et al. (2012) (India) | NR | 20 | 10/10/0 | 6/4; 4/6; 0/0 | 0.0% / 0.0% / - | 32.4±3.2 / 35.2±3.1 / - | PPD≥5 mm and CAL≥3 mm with RBL | Immunoturbidimetry |
| 2012 | Han et al. (2012) (Korea) | Korea Science and Engineering Foundation of the Ministry of Education, Science, and Technology (Grant 2010-0014141), the Ministry of Environment (Grant 900-20060032), and the National Research Foundation of Korea Grant (grant 2010-0029479) | 93 | 73/20/0 | 30/43; 11/9; 0/0 | 17.8% / 15.0% / - | 40.9±10.1 / 48.7±9.3 / - | Community Periodontal Index | Immunoturbidimetry |
| 2012 | Rai et al. (2012) (India) | NR | 87 | 32/55/0 | 15/17; 30/25; 0/0 | 20.0% / 23.0% / - | 44.1±12.5 / 45.5±14.3 / - | PPD≥5mm or any CAL | ELISA |
| 2012 | Ay et al. (2012) (Turkey) | Scientific and Technological Research Council of Turkey (Grant no 106S187 SBAG 3435) | 57 | 20/21/16 | 8/12; 10/11; 7/9 | 0.0% / 0.0% / 0 | 33.0±5.5 / 47.0±7.25 / 37.0±5.25 | AAP 1999 | CLIA |
| 2012 | Loo et al. (2012) (China) | NR | 152 | 108/44/0 | 69/39; 26/18; 0/0 | 0.0% / 0.0% / - | 42.9±9.7 / 49.3 ±13.6 / - | AAP 1999 | Immunoturbidimetry |
| 2011 | Pejcic et al. (2011) (Serbia) | NR | 75 | 25/50/0 | 7/18; 23/27; 0/0 | 64.0% / 52.0% / - | 26.3±3.6 / 41.3±17.4 / - | PPD≥5mm with RBL | RIA |
| 2011 | Freitas et al. (2011) (Brazil) | No | 62 | 31/31/0 | 11/20; 10/21; 0/0 | 0.0% / 0.0% / - | 39.1±7.8 / 40.5±6.5 / - | ≥4 teeth with at least one site with PPD≥4 mm and CAL≥3 mm in the same site | Nephelometry |
| 2011 | Masi et al. (2011) (United Kingdom) | British Heart Foundation (RG2008/08 and FS/06/053) | 563 | 207 / 285 / 71 | 88/119; NR; NR | 41.5% / NR / NR | 46.8 ± 10.2 / NR / NR | AAP 1999 Consensus classification | Immunoturbidimetry |
| 2011 | Tang et al. (2011) (China) | NR | 43 | 18/25/0 | NR/NR; NR/NR; 0/0 | NR / NR / - | NR / NR / - | CAL>1mm | ELISA |
| 2010 | Cairo et al. (2010) (Italy) | NR | 45 | 0/21/24 | 0/0; 12/9; 12/12 | - / 42.0% / 42.00% | - / 36.8±3.5 / 25.9±3.8 | AAP 1999 | Nephelometry |
| 2010 | Pradeep et al. (2010) (India) | NR | 30 | 15/15/0 | NR/NR; NR/NR; 0/0 | 0.0% / 0.0% / - | NR / NR / - | PPD≥5 mm, and CAL≥3 mm, and RBL | ELISA |
| 2010 | Thakare et al. (2010) (India) | NR | 30 | 15/15/0 | 9/6; 9/6; 0/0 | 0.0% / 0.0% / - | 39.6±0.1 / 40.1±0.1 / - | RBL ≥ 30%, at least 50% of dentition and CAL≥ 4mm at ≥ 6 sites | Immunoturbidimetry |
| 2010 | Liu et al. (2010) (China) | NR | 80 | 40/40/0 | 19/21; 23/17; 0/0 | 0.0% / 0.0% / - | 47.7±5.7 / 50.9±6.0 / - | CAL ≥ 1.6mm, sites with interproximal CAL ≥3mm distributed through ≥3 quadrants or ≥6 teeth | Immunoturbidimetry |
| 2010 | D’Aiuto et al. 2010 (United Kinddown) | Yes | 201 | 56/118/27 | 28/28; NR/NR; NR/NR; | 31.8% / NR / NR | 46.4±7.4 / NR / NR | 1999 Consensus Classification of Periodontal Diseases | Immunoturbidimetry |
| 2009 | Buhlin et al. (2009) (Sweden) | Yes | 116 | 48/68/0 | 23/25; 36/32; 0/0 | 43.8% / 50.0% / - | 53.1±7.5 / 53.9±8.8 / - | PPD≥4 mm and BOP≤35% | ELISA |
| 2009 | Nicu et al. (2009) (The Netherlands) | Yes | 162 | 57/105/0 | 19/43; 41/64; 0/0 | 15.8% / 28.6% / - | 41.5±10.8 / 43.8±8.4 / - | ≥7 teeth with ≥50% bone loss | Nephelometry |
| 2009 | Gani et al. (2009) (India) | NR | 28 | 14/28/0 | NR/NR; NR/NR; 0/0 | 0.0% / 0.0% / - | NR / NR / - | Armitage 1999 | Immunoturbidimetry |
| 2009 | Bezerra et al. (2009) (Brazil) | NR | 62 | 31/31/0 | 12/19; 10/19; 0/0 | 0.0% / 0.0% / - | NR / NR / - | ≥30% of CAL≥5 mm | Nephelometry |
| 2009 | Sun et al. (2009) (China) | National Natural Science Foundations of China (30471882); National Key Project of Scientific and Technical Supporting Programs of China (2007BAZ18B02) | 149 | 65/0/84 | 21/44; 0/0; 38/46 | 0.0% / 0.0% / 15.48% | 26.8±4.7 / - / 27.8±5.8 | AAP 1999 | ELISA |
| 2008 | Pitiphat et al. (2008) (Thailand) | Yes | 121 | 38/83/0 | 1/37; 31/52; 0/0 | 2.6% / 15.7% / - | 34.9±8.3 / 41.9±11.2 / - | >30% sites with PPD≥5 mm | Nephelometry |
| 2007 | Bizzarro et al. (2007) (The Netherlands) | Yes | 130 | 39/91/0 | 14/25; 43/48; 0/0 | 31.0% / 46.2% / - | 39.3±10.6 / 44.7±8.7 / - | ≥7 teeth with ≥50% bone loss | Nephelometry |
| 2007 | Keles et al. (2007) (Turkey) | NR | 40 | 20/20/0 | 15/5; 14/6; 0/0 | 0.0% / 0.0% / - | 48.3±0.5 / 47.6±0.9 / - | Mean CAL>3 mm and PPD≥4 mm with periodontal sites greater than 30% | Nephelometry |
| 2007 | Tuter et al. (2007) (Turkey) | NR | 37 | 17/20/0 | NR/NR; NR/NR; 0/0 | 0.0% / 0.0% / - | NR / NR / - | RBL and CAL≥6 and PPD>4 mm | Nephelometry |
| 2007 | Yamazaki et al. (2007) (Japan) | Ministry of Education, Science Sports and Culture of Japan (16390613, 17659655 and 19390536) | 92 | 37/55/0 | 18/19; 24/31; 0/0 | 0.0% / 65.5% / - | 48.6±1.5 / 47.2±1.7 / - | PPD>3mm | Nephelometry |
| 2006 | Salzberg et al. (2006) (USA) | Yes | 281 | 91/0/190 | 47/44; 0/0; 64/126 | 42.0% / - / 40.53% | 30.7±8.2 / - / 27.1±8.7 | AgP: CAL≥5mm on 8 or more teeth, at least three of which are not first molars and incisors, and <35 years of age | ELISA |
| 2006 | Havemose-Poulsen et al. (2006) (Danmark) | Yes | 70 | 25/0/45 | 10/15; 0/0; 16/29 | 8.0% / - / 51.1% | 25.0±3.1 / - / 24.2±5.8 | Armitage 1999 | Immunoturbidimetry |
| 2005 | Dye et al. (2005) (USA) | Yes | 2973 | 2385/588/0 | NR/NR; NR/NR; 0/0 | NR / NR / - | NR / NR / - | ≥1 site with 3 mm or CAL≥4 mm PPD | Nephelometry |
| 2005 | Yamazaki et al. (2005) (Japan) | Yes | 47 | 23/24/0 | 10/13; 9/15; 0/0 | 0.0% / 0.0% / - | 43.8±6.6 / 40.5±11.1 / - | Armitage 1999 | Nephelometry |
| 2004 | Deliargyris et al. (2004) (USA) | NR | 40 | 33/7/0 | NR/NR; NR/NR; 0/0 | 66.6% / 99.9% / - | 64.0±3.0 / - / - | Armitage 1999 | ELISA |
| 2003 | Amar et al. (2003) (USA) | Yes | 55 | 29/26/0 | 18/11; 16/10; 0/0 | 0.0% / 0.0% / - | 41.0±9.9 / 42.0±10.0 / - | ≥6 teeth with PPD>5 mm and CAL≥3 mm in 3 aspects of tooth | Nephelometry |
| 2003 | Craig et al. (2003) (USA) | NR | 69 | 25/44/0 | 7/18; 34/10; 0/0 | 8.0% / 34.0% / - | 29.9±1.1 / 38.7±1.3 / - | Haffajee et al. 1983 | ELISA |
| 2002 | Glurich et al. (2002) (USA) | U.S. Public Health Service grants DEO 7926, DEO 4898, and DE12085 from the NIDCR. | 46 | 26/20/0 | NR/NR; NR/NR; 0/0 | NR / NR / - | NR / NR / - | Mean of CAL≥4 mm | ELISA |
| 2001 | Noack et al. (2001) (USA) | Yes | 174 | 65/109/0 | 25/40; 61/48; 0/0 | 6.2% / 22.0% / - | 54.0±11.2 / 63.8±11.8 / - | CAL>2 | RIA |
| 1999 | Fredriksson et al. (1999) (Sweden) | Yes | 83 | 43/40/0 | 18/25; 20/20; 0/0 | 32.6% / 47.5% / - | 50.6±7.6 / 52.4±8.4 / - | ≥ 6 sites with CAL ≥5 mm | Immunoturbidimetry |
| 1997 | Ebersole et al. (1997) (USA) | Upjohn Co. and a IADR fellowship | 75 | 35/40/0 | NR/NR; NR/NR; 0/0 | NR / NR / - | NR / NR / - | RBL with CAL, BOP and PPD≥ 5 mm | ELISA |

RBL – Radiographic Bone Loss; CAL - Clinical Attachment Loss; PPD – Periodontal Pocket Depth; BOP – Bleading on probing; H – Healthy; CP – Chronic Periodontitis; AgP – Aggressive Periodontitis; NR – Not reported; NHANES - National Health and Nutrition Examination Survey; AAP – American Academy of Periodontology; CDC – Centre for Disease Control and Prevention; EFP – European Federation of Periodontology; CLIA - Chemiluminescence Immunoassay; RIA – Radioimmunoassay; ELISA - enzyme-linked immunosorbent assay

Supplementary Table 6. Studies characteristics comparing levels of hs-CRP baseline and after periodontal treatment of otherwise healthy.

| **Year** | **Authors (Year) (Country)** | **Funding** | **N. Total** | **Sex (M/F) (n)** | **Smokers (%)** | **Age (mean ± SD)** | **PD Diagnostic Criteria** | **Treatment type** | **CRP method** |
| --- | --- | --- | --- | --- | --- | --- | --- | --- | --- |
| 2021 | Suvan et al. (2021) | University of Pisa | 57 | 28 / 27 | 0.0 | 50.0 ± 8.0 | PPD ≥ 5mm and marginal alveolar bone loss with > 30% sites affected | Intensive | ELISA |
| 2020 | Johnston et al. (2020) (UK) | University of Glasgow's PhD programme and Dentsply Sirona | 37 | 18 / 19 | 29.7 | 44.1 ± 3.9 | PPD ≥5 mm on 2 or more teeth at non‐adjacent sites with cumulative PPD of ≥40 mm | Intensive | Immunoturbidimetry |
| 2020 | Preshaw et al. (2020) (UK) | Dunhill Medical Trust, Grant/Award Number: R63/1107; Philips Research, Grant/Award Number: DRC-0417; National Institute for Health Research, Grant/Award Number: DHCS/03/G121/46 | 44 | NR / NR | NR | 47.5 ± 8.0 | Armitage 1999 | Non-intensive | ELISA |
| 2019 | Luthra et al. (2019) (India) | No | 30 | 22 / 8 | 0.0 | NR | PPD ≥6 mm and evidence of 30% RBL | Intensive | Immunoturbidimetry |
| 2019 | Caribé et al. (2019) (Brazil) | Coordination of Improvement of Higher Education Personnel - Brazil (São Paulo) (CAPES) - Finance Code 001 to P.M.V. Caribé | 40 | NR / NR | 0.0 | 58.2 ± 7.3 | Minimum of 6 teeth, with at least 1 noncontiguous interproximal site with PPD and CAL ≥ 5 mm, and 30% of sites with PPD and CAL ≥ 4 mm and BoP | Non-intensive | Nephelometry |
| 2019 | Leite et al. (2019) (Brazil) | Maranhão State | 33 | 13 / 20 | 0.0 | NR | Armitage 2004 | Non-intensive | ELISA |
| 2019 | Yashima et al. (2019) (Japan) | Pfizer Inc, New York, NY, USA | 29 | 13 / 16 | 0.0 | 59.4 ± 8.9 | PPD > 5 mm | Intensive | ELISA |
| 2019 | Lobão et al. (2019) (Brazil) | Federal University of the Maranhão and the Fundação de Amparo à Pesquisa e ao Desenvolvimento Científico e Tecnológico do Maranhão | 33 | 13 / 20 | 0.0 | 41.1 ± 7.8 | ≥ 2 teeth with CAL ≥ 6 mm and PPD ≥ 5 mm in one or more sites | Non-intensive | ELISA |
| 2019 | Graziani et al. (2019) (Italy) | Institut Straumann AG (Basel, Switzerland) | 19 | 8 / 11 | 0.0 | 50.9 ± 10.1 | proximal CAL≥3 mm in ≥2 non-adjacent teeth, BoP on at least 25% of total sites and RBL | Intensive | ELISA |
| 2019 | Kocher et al. (2019) (Germany) | German Research Foundation (Deutsche Forschungsgemeinschaft: EH 365 1-1) | 218 | 104 / 114 | 25.0 | 51.7 ± 10.8 | Localized PD was defined as <38%, and generalized PD was defined as ≥38% of teeth with PPD≥6 mm | Intensive | Cardiac-specific claim |
| 2018 | Tasdemir et al. (2018) (Turkey) | NR | 38 | 13 / 7 | 0.0 | 47.5 ± 8.8 | Presence of ≥ 5 teeth with ≥ 1 sites with PPD≥5 mm, CAL≥2 mm, and the presence of BoP | Intensive | ELISA |
| 2018 | Morozumi et al. (2018) (Japan) | Pfizer Inc., New York, NY, USA | 29 | 13 / 16 | 0.0 | 59.4 ± 8.9 | moderate: 3-4 mm CAL and severe: ≥5 mm loss, generalized PD: >30% of sites affected | Intensive | ELISA |
| 2018 | Martinez-Herrera et al. (2018) (Spain) | Carlos III Health Institute (PI16/00301), Valencian Regional Ministry of Education (GV/2016/169) and from FISABIO (UGP 15- 220), and European Regional Development Fund | 48 | 21 / 27 | 25.0 | 39.4 ± 8.3 | ≥4 teeth with ≥1 sites with PPD≥4 mm and CAL ≥3 mm | Intensive | Nephelometry |
| 2017 | Zhang et al. (2017) (China) | NR | 58 | 30 / 28 | 46.6 | 34.8 ± 6.1 | mild PD (CAL 1-2 mm), moderate PD (CAL 3-4 mm), and severe PD (CAL≥5 mm) | Non-intensive | ELISA |
| 2017 | Mallapragada et al. (2017) (India) | No | 25 | 14 / 11 | 0.0 | 38.4 ± 3.3 | Disease Control and Prevention 2007 criteria | Non-intensive | Nephelometry |
| 2017 | Alyousef et al. (2017) (India) | No | 61 | NR / NR | 0.0 | 39.4 ± 21.6 | Moderate PD (≥2 interproximal sites with CAL≥4 mm or ≥2 interproximal sites with PPD≥5 mm) and severe PD (≥2 interproximal sites with CAL of ≥6 mm and ≥1 interproximal site with PPD of ≥5 mm) | Non-intensive | Immunoturbidimetry |
| 2017 | Ertugrul et al. (2017) (Turkey) | No | 20 | NR / NR | 0.0 | NR | CAL >30% of their total existing dental areas and PPD ≥5 mm along with bone destruction | Non-intensive | ELISA |
| 2017 | Bozoglan et al. (2017) (Turkey) | University of YYU (Committee of Research 2014-SBE-D016) | 20 | NR / NR | 0.0 | 49.6 ± 8.3 | PPD ≥ 5 mm at least 6 total sites of at least four teeth with one root, and CAL ≥ 4 mm | Non-intensive | ELISA |
| 2017 | Tasdemir et al. (2017) (Turkey) | Scientific Research Projects of Erciyes University (Grant no TDH 2015-5657) | 15 | 9 / 5 | 0.0 | 49.2 ± 9.2 | ≥5 teeth with ≥1 sites with a PPD ≥5 mm, CAL ≥2 mm, and presence of BoP | Intensive | ELISA |
| 2017 | Kurgan et al. (2017) (Turkey) | Ankara University Scientific Research Projects Office, Ankara, Turkey (08B3334005) | 15 | 7 / 8 | NR | 42.1 ± 7.4 | CAL > 5 mm on at least 3 teeth, along with clinical inflammation | Non-intensive | Immunoturbidimetry |
| 2017 | Souza et al. (2017) (Brazil) | No | 22 | 8 / 14 | 0.0 | 44.6 ± 6.9 | ≥5 teeth with at least one site with PPD ≥5 mm and CAL ≥3 mm | Non-intensive | Immunoturbidimetry |
| 2016 | Vargas-Villafuerte et al. (2016) (Brazil) | The State of São Paulo Research Foundation grants 2012/20971-8 | 18 | 0 / 18 | 0.0 | 45.3 ± 7.1 | 6 teeth with PPD ≥ 5 mm and CAL ≥ 5 mm | Non-intensive | Immunoturbidimetry |
| 2016 | Žekonis et al. (2016) (Lithuania) | No | 43 | 22 / 21 | 0.0 | 41.2 ± 6.0 | PPD>6 mm on at least 2 teeth, and horizontal and vertical RBL | Non-intensive | Immunoturbidimetry |
| 2016 | Zuza et al. (2016) (Brazil) | São Paulo Research Foundation (Grant no 2010/17614-3) | 26 | 8 / 18 | 0.0 | 42.7 ± 7.5 | At least 6 teeth with PPD ≥5 mm and CAL ≥3 mm, and BoP | Non-intensive | ELISA |
| 2016 | Ling et al. (2016) (United Kingdown) | Birmingham and the Black Country Comprehensive Local Research Network (Grant no. 10318) and the University of Birmingham (Sponsor reference no. RG_10-077) | 20 | 12 / 8 | 0.0 | 46.0 ± 8.0 | Consensus criteria of the European Federation of Periodontology, 2005 | Non-intensive | Immunoturbidimetry |
| 2016 | Duzagac et al. (2016) (Turkey) | Istanbul University (41707) | 15 | 7 / 8 | 0.0 | 41.1 ± 7.1 | PPD ≥ 4 mm in ≥ 30%BoP ≥ 50%; interproximal CAL> 2 mm in ≥ 20% of periodontal sites; and RBL | Non-intensive | Immunoturbidimetry |
| 2015 | Gupta et al. (2015) (India) | No | 50 | 28 / 22 | 0.0 | 36.6 ± 10.1 | ≥4 sites with PPD ≥ 5mm and at least 4 sites with CAL > 2 mm | Non-intensive | ELISA |
| 2015 | Torumtay et al. (2015) (Turkey) | Unit of Scientific Research Projects, Suleyman Demirel University, Turkey (Grant no 3244-D1-12) | 25 | 10 / 12 | 0.0 | 48.3 ± 7.4 | ≥30% of the teeth present in the whole mouth with CAL ≥ 2 mm | Non-intensive | ELISA |
| 2015 | Graziani et al. (2015) (UK) | Unit of Scientific Research Projects, Suleyman Demirel University, Turkey (Grant no 3244-D1-12) | 19 | 10 / 9 | 30.0 | 48.0 ± 9.0 | Interproximal CAL ≥3 mm in ≥2 nonadjacent teeth, BoP ≥25%, and RBL | Non-intensive | Immunoturbidimetry |
| 2015 | Graziani et al. (2015) (UK) | Unit of Dentistry and Oral Surgery of the University of Pisa and by the Italian Ministry Health and the Tuscan Region (Grant no GR-2009-1592229) | 19 | 9 / 10 | 44.0 | 46.0 ± 12.0 | Interproximal CAL ≥3 mm in ≥2 nonadjacent teeth, BoP ≥25%, and RBL | Intensive | Immunoturbidimetry |
| 2015 | Fentoglu et al. (2015) (Turkey) | Scientific and Technological Research Council of Turkey (Grant no SBAG 3583-107S056) | 28 | NR / NR | 0.0 | NR | ≥4 teeth with a PPD ≥5 mm and a CAL ≥2 mm at the same time | Non-intensive | CLIA |
| 2015 | Habashneh et al. (2015) (Jordan) | NR | 21 | 7 / 14 | 0.0 | 39.0 ± 10.2 | >2 interproximal sites with PPD> 5 mm , RBL and CAL> 6 mm | Non-intensive | ELISA |
| 2014 | Mohan et al. (2014) (India) | Saraswati Dental College and Hospital, Lucknow, India | 24 | NR / NR | 0.0 | NR | PPD ≥ 5mm, a CAL ≥ 3mm, and RBL, ≥ 30% of the sites involved | Intensive | Immunoturbidimetry |
| 2014 | Caúla et al. (2014) (Brazil) | No | 32 | 20 / 12 | 12.5 | 44.4 ± 5.5 | Page & Eke 2007 | Non-intensive | CLIA |
| 2014 | Deore et al. (2014a) (India) | NR | 30 | NR / NR | 0.0 | 45.0 ± 4.9 | Disease Control and Prevention 2007 criteria | Non-intensive | Immunoturbidimetry |
| 2014 | Deore et al. (2014b) (India) | NR | 30 | NR / NR | 0.0 | 44.5 ± 5.2 | Disease Control and Prevention 2007 criteria | Non-intensive | Immunoturbidimetry |
| 2014 | Almaghlouth et al. (2014) (Switzerland) | The Swiss National Science Foundation (Grant no. 320030-122089) | 21 | NR / NR | NR | NR | ≥4 teeth with a PPD>4 mm, CAL≥2 mm, and RBL | Intensive | ELISA |
| 2013 | Siqueira et al. (2013) (Brazil) | National Council of Technological and Scientific Development and Coordination for the Improvement of Higher Level or Education Personnel | 8 | 3 / 5 | NR | 49.5 ± 2.1 | ≥3 teeth with CAL ≥ 4 mm and a PPD ≥ 5 mm | Non-intensive | Immunoturbidimetry |
| 2013 | Patil et al. (2013) (India) | No | 20 | 12 / 8 | 0.0 | NR | PPD≥ 4 to 5 mm and CAL of 1 to 3 mm | Non-intensive | Immunoturbidimetry |
| 2013 | George et al. (2013) (India) | No | 20 | 12 / 8 | 0.0 | NR | CAL ≥5 mm and at least 50% of teeth affected | Non-intensive | Immunoturbidimetry |
| 2013 | Eickholz et al. (2013) (Germany) | German Society of Periodontology, the German Society of Dental, Oral, and Maxillofacial Medicine, and the New Working Group for Periodontology | 31 | 19 / 12 | NR | 52.8 ± 7.6 | PPD ≥ 3.6 mm and vertical CAL ≥ 5 mm at more than 30% of sites; PPD ≥ 7 mm at a minimum of 4 sites | Intensive | Immunoturbidimetry |
| 2013 | Altay et al. (2013) (Turkey) | Ankara University Research Foundation (Grant 2008-08B3334001) | 24 | 9 / 15 | 25.0 | 42.5 ± 8.7 | At least 5 teeth with PPD ≥ 5mm in ≥1 sites , CAL ≥2 mm, and positive BoP | Intensive | ELISA |
| 2011 | Vilela et al. (2011) (Brazil) | Fundação Instituto Mineiro de estudos e Pesquisas em Nefrologia | 20 | 9 / 11 | 0.0 | 43.4 ± 11.0 | At least 2 teeth with PPD ≥ 5 mm, at least 1 site with a CAL ≥6 mm, and RBL | Non-intensive | Nephelometry |
| 2011 | Kamil et al. (2011) (Jordan) | Partially supported by the University of Science and Technology Graduate Student Research Fund | 18 | 10 / 8 | 0.0 | 46.7 ± 3.4 | At least 6 teeth with PPD> 5 mm and CAL≥ 3 mm in 3 sites of each involved tooth | Non-intensive | Immunoturbidimetry |
| 2011 | Li et al. (2011) (China) | Hong Kong Research Grants Council (HKU 7518/05M and HKU766909M), The University of Hong Kong (CRCG Funds 200507176137, 2006 07176038, 200707176095 and 200907176 052) and the Sun Chieh Yeh Heart Foundation | 25 | 10 / 15 | 0.0 | 58.6 ± 11.6 | More than 6 sites with PPD≥4 mm, over 25% of sites with CAL≥5 mm | Non-intensive | Immunoturbidimetry |
| 2011 | Tamaki et al. (2011) (Japan) | Grants-in-Aid for Scientific Research (20791641 & 22792119) from the Ministry of Education, Culture, Sports, Science and Technology, Tokyo, Japan | 22 | 10 / 12 | 0.0 | 48.0 ± 8.5 | At least 4 teeth with PPD ≥4 mm | Non-intensive | ELISA |
| 2010 | Nagarale et al. (2010) (India) | No | 15 | NR / NR | 0.0 | NR | PPD ≥6 mm in minimum of 6 teeth | Non-intensive | Latex agglutination method |
| 2010 | Taylor et al. (2010) (Australia) | National Health and Medical Research Council of Australia (Grant no: 219192) and the Ramaciotti Foundation | 61 | 29 / 32 | 31.1 | 52.1 ± 13.3 | Six or more sites with PPD ≥ 5 mm and CAL ≥ 2 mm | Non-intensive | ELISA |
| 2010 | Acharya et al. (2010) (India) | NR | 15 | 7 / 8 | 0.0 | 45.1 ± 7.1 | CAL≥3 mm and PPD ≥ 5 mm | Non-intensive | Latex agglutination method |
| 2010 | Graziani et al. (2010a) (Italy) | Self-supported by the Unit of Dentistry and Oral Surgery of the University of Pisa | 14 | 8 / 6 | 50.0 | 49.0 ± 15.0 | Armintage 1999 | Intensive | Immunoturbidimetry |
| 2010 | Graziani et al. (2010b) (Italy) | Self-supported by the Unit of Dentistry and Oral Surgery of the University of Pisa | 20 | 12 / 8 | 55.0 | 48.0 ± 9.0 | PPD ≥5 mm on at least 30% of their total sites | Intensive | Immunoturbidimetry |
| 2010 | Shimada et al. (2010) (Japan) | Grant-in-Aids for Scientific Research (19791608, 19390535) from the Ministry of Education, Culture, Sports, Science and Technology of Japan, Tokyo, Japan | 33 | 8 / 25 | 33.3 | 55.1 ± 7.8 | Armitage 1999 | Non-intensive | ELISA |
| 2010 | Kardesler et al. (2010) (Turkey) | University of Louisville and a grant from the Ege University Research Foundation (2005 Disx 015) | 15 | 9 / 6 | 60.0 | 51.3 ± 8.6 | At least 4 teeth in each jaw with a PPD ≥ 5 mm, CAL ≥ 4 mm, at least 2 single-rooted teeth with a PPD of 6 to 9 mm, and BoP | Non-intensive | ELISA |
| 2010 | Radafshar et al. (2010) (Iran) | GUMS secretary of research and technology, Rasht, Iran | 35 | 18 / 17 | 0.0 | NR | PPD and CAL ≥ 5 mm, and RBL | Non-intensive | ELISA |
| 2009 | Pinho et al. (2009) (Brazil) | NR | 15 | NR / NR | 0.0 | NR | Criteria of Machtei et al. 1992 | Non-intensive | Nephelometry |
| 2009 | Renvert et al. (2009) (Sweden) | NR | 29 | 18 / 11 | 52.0 | 56.7 ± 9.1 | ≥ 10 pockets with PPD ≥ 5mm, of which at least 4 PPD of 6 to 9 mm | Non-intensive | ELISA |
| 2009 | Behle et al. (2009) (USA) | NIH grant DE015649 (National Institutes of Health, Bethesda, MA, USA) and gift from Colgate-Palmolive | 30 | 14 / 16 | 0.0 | 43.3 ± 13.3 | RBL ≥30% of the root length at multiple sites; ≥2 teeth/quadrant with a PPD≥6 mm and concomitant CAL≥3 mm | Non-intensive | ELISA |
| 2009 | Marcaccini et al. (2009) (Brazil) | State of São Paulo Research Foundation, São Paulo, SP, Brazil, and the National Research Council | 25 | 9 / 16 | 0.0 | 44.9 ± 5.5 | At least 2 teeth with PPD ≥5 mm, CAL ≥6 mm, and RBL | Non-intensive | Spectrophotometer |
| 2008 | Ushida et al. (2008) (Japan) | Grants-in-Aid for Scientific Research from the Japan Society for the Promotion of Science (nos. 18390561, 15390642 and 18592258) | 12 | NR / NR | NR | NR | At least 5 teeth and 2 pocket sites with PPD ≥5mm in each quadrant, and RBL | Intensive | Nephelometry |
| 2008 | Ushida et al. (2008) (Japan) | Grants-in-Aid for Scientific Research from the Japan Society for the Promotion of Science (nos. 18390561, 15390642 and 18592258) | 12 | NR / NR | NR | NR | At least 5 teeth and 2 pocket sites with PPD ≥5mm in each quadrant, and RBL | Intensive | Nephelometry |
| 2008 | Ushida et al. (2008) (Japan) | Grants-in-Aid for Scientific Research from the Japan Society for the Promotion of Science (nos. 18390561, 15390642 and 18592258) | 12 | NR / NR | NR | NR | At least 5 teeth and 2 pocket sites with PPD ≥5mm in each quadrant, and RBL | Non-intensive | Nephelometry |
| 2007 | Tonetti et al. (2007) (Italy) | University College London Hospital Research and Development Directorate, the British Heart Foundation, the European Research Group on Periodontology, the Periodontology Research Fund of the Eastman Dental Institute, Johnson & Johnson | 59 | 30 / 29 | 31.0 | 47.8 ± 6.3 | PPD >6 mm and marginal alveolar bone loss of >30%, with 50% or more teeth affected | Intensive | Immunoturbidimetry |
| 2007 | Tonetti et al. (2007) (Italy) | University College London Hospital Research and Development Directorate, the British Heart Foundation, the European Research Group on Periodontology, the Periodontology Research Fund of the Eastman Dental Institute, Johnson & Johnson | 61 | 30 / 31 | 31.0 | 47.7 ± 7.9 | PPD >6 mm and marginal alveolar bone loss of >30%, with 50% or more teeth affected | Intensive | Immunoturbidimetry |
| 2006 | D'Aiuto et al. (2006) (UK) | Orapharma and by the Periodontal Research Fund of the Eastman Dental Institute | 20 | 10 / 10 | 25.0 | 47.0 ± 7.0 | PPD >6 mm and marginal alveolar bone loss of >30%, with 50% or more teeth affected | Non-intensive | Immunoturbidimetry |
| 2006 | Elter et al. (2006) (USA) | NR | 32 | 20 / 12 | 23.0 | 42.0 ± 6.0 | Ranney R. Classification of periodontal diseases. Periodontol 2000. 1993;2:13 - 25. | Non-intensive | Nephelometry |
| 2005 | Seinost et al. (2005) (Austria) | NR | 30 | 14 / 16 | 26.0 | 40,2 ± 4.0 | Brown LJ, Loe H. Prevalence, extent, severity and progression of periodontal disease. Periodontol 2000. 1993;2:57 - 71. | Non-intensive | Nephelometry |
| 2005 | D'Aiuto et al. (2005a) (UK) | Unrestricted educational grant from Orapharma and by the Periodontal Research Fund of the Eastman Dental Institute | 20 | 12 / 8 | 25.0 | 48.0 ± 7.0 | PPD >6 mm and marginal alveolar bone loss of >30%, with 50% or more of their teeth affected | Intensive | Immunoturbidimetry |
| 2005 | D'Aiuto et al. (2005a) (UK) | Unrestricted educational grant from Orapharma and by the Periodontal Research Fund of the Eastman Dental Institute | 20 | 12 / 8 | 25.0 | 48.0 ± 7.0 | PPD >6 mm and marginal alveolar bone loss of >30%, with 50% or more of their teeth affected | Non-Intensive | Immunoturbidimetry |
| 2004 | D'Aiuto et al. (2004d) (UK) | Eastman Dental Institute, and Italian Society of Periodontology and the European Union | 14 | 6 / 8 | 14.3 | 48.0 ± 6.0 | PPD >6 mm and marginal alveolar bone loss of >30%, with 50% or more of their teeth affected | Intensive | Immunoturbidimetry |
| 2004 | Ide et al. (2004) (UK) | British Dental Association Shirley Glasstone Hughes Research Award | 23 | 14 / 9 | 0.0 | 40.7 ± 10.3 | AAP 1999 | Non-Intensive | Nephelometry |
| 2004 | D'Aiuto et al. (2004a) (UK) | Electro-Medical Systems, Switzerland, and by the Periodontal Research Fund of the Eastman Dental Institute | 94 | 51 / 43 | 0.0 | 46.0 ± 9.0 | PPD > 6 mm and marginal alveolar bone loss greater than 30% | Non-intensive | Immunoturbidimetry |
| 2003 | Ide et al. (2003) (UK) | British Dental Association Shirley Glasstone Hughes Research Award | 24 | 13 / 11 | 0.0 | 47.8 ± 7.5 | AAP 1999 | Non-intensive | Nephelometry |

RBL – Radiographic Bone Loss; CAL - Clinical Attachment Loss; PPD – Periodontal Pocket Depth; BOP – Bleading on probing; H – Healthy; CP – Chronic Periodontitis; AgP – Aggressive Periodontitis; NR – Not reported; NHANES - National Health and Nutrition Examination Survey; AAP – American Academy of Periodontology; CDC – Centre for Disease Control and Prevention; EFP – European Federation of Periodontology; CLIA - Chemiluminescence Immunoassay; ELISA - enzyme-linked immunosorbent assay

Supplementary Table 7. Newcastle-Ottawa Scale In Non-randomized Studies – for case control studies

| **Study** | **SELECTION** | | | | **COMPARABILITY** | **EXPOSURE** | | | **RoB Score** |
| --- | --- | --- | --- | --- | --- | --- | --- | --- | --- |
|  | **Is the case definition adequate?** | **Representativeness of the cases?** | **Selection of controls?** | **Definition of controls?** | **Comparability of cases and controls of design or analysis?** | **Ascertainment of exposure?** | **Same method of ascertainment for cases and controls** | **Non-response rate?** |  |
| Ebersole et al. 1997 | a | b | c | a | a/b | a | a | a | 7 |
| Fredriksson et al. 1999 | a | b | c | a | a/b | a | a | c | 6 |
| Noack et al. 2001 | a | a | a | a | a/b | a | a | a | 9 |
| Glurich et al. 2002 | a | b | a | a | a/b | a | a | a | 8 |
| Amar et al. 2003 | a | a | a | a | a/b | a | a | a | 9 |
| Craig et al. 2003 | a | a | a | a | a/b | a | a | a | 9 |
| Deliargyris et al. 2004 | c | b | b | a | a/b | a | a | a | 6 |
| Dye et al. 2005 | a | a | a | a | a/b | a | a | a | 9 |
| Yamazaki et al. 2005 | a | a | b | a | a/b | a | a | a | 8 |
| Salzberg et al. 2006 | a | a | a | b | a/b | a | a | a | 8 |
| Havemose-Poulsen et al. 2006 | a | a | a | a | a/b | a | a | a | 9 |
| Bizzarro et al. 2007 | a | b | a | a | a/b | a | a | a | 8 |
| Keles et al. 2007 | a | b | a | a | a/b | a | a | a | 8 |
| Tuter et al. 2007 | a | a | a | a | a/b | a | a | a | 9 |
| Yamazaki et al. 2007 | c | b | b | a | a/b | a | a | a | 6 |
| Pitiphat et al. 2008 | a | b | c | a | a/b | a | a | a | 7 |
| Buhlin et al. 2009 | a | a | a | a | a/b | a | a | a | 9 |
| Nicu et al. 2009 | c | b | a | a | a/b | a | a | a | 7 |
| Gani et al. 2009 | a | b | a | a | a/b | a | a | a | 8 |
| Bezerra et al. 2009 | a | b | a | a | a/b | a | a | a | 8 |
| Sun et al. 2009 | a | b | a | a | a/b | a | a | a | 8 |
| Pradeep et al. 2010 | a | b | a | a | a | a | a | a | 7 |
| Cairo et al. 2010 | a | b | b | a | a/b | a | a | a | 7 |
| D'Aiuto et al. 2010 | a | b | a | a | a/b | a | a | a | 8 |
| Thakare et al. 2010 | a | b | b | a | a/b | a | a | a | 7 |
| Liu et al. 2010 | a | a | b | a | a/b | a | a | a | 8 |
| Pejcic et al. 2011 | a | b | a | a | a/b | a | a | a | 8 |
| Masi et al. 2011 | a | a | a | a | a/b | a | a | a | 9 |
| Freitas et al. 2011 | a | b | a | a | a/b | a | a | a | 8 |
| Tang et al. 2011 | a | a | a | a | a/b | a | a | a | 9 |
| Wohlfeil et al. 2012 | a | b | a | a | a/b | a | a | a | 8 |
| Miyashita et al. 2012 | c | b | c | a | a/b | a | a | a | 6 |
| Ay et al. 2012 | a | a | a | a | a/b | a | a | a | 9 |
| Gani et al. 2012 | a | b | a | a | a/b | a | a | a | 8 |
| Miyazawa et al. 2012 | c | b | a | b | a/b | a | a | a | 6 |
| Chopra et al. 2012 | a | b | a | a | a/b | a | a | a | 8 |
| Pradeep et al. 2012 | a | b | a | a | a/b | a | a | a | 8 |
| Han et al. 2012 | a | a | c | a | a/b | a | a | a | 8 |
| Rai et al. 2012 | a | b | c | a | a/b | a | a | a | 7 |
| Loo et al. 2012 | a | a | b | a | a/b | a | a | a | 8 |
| Tian et al. 2013 | a | b | a | a | a/b | a | a | a | 8 |
| Andrukhov et al. 2013 | a | b | c | a | a/b | a | a | a | 7 |
| Priyanka et al. 2013 | a | a | a | a | a/b | a | a | a | 9 |
| Kalra et al. 2013 | a | a | a | a | a/b | a | a | a | 9 |
| Pradeep et al. 2013 | a | a | a | a | a/b | a | a | a | 9 |
| Kumari et al. 2014 | a | b | a | a | a/b | a | a | a | 8 |
| Nethravathy et al. 2014 | a | a | a | a | a/b | a | a | a | 9 |
| Bansal et al. 2014 | a | b | a | a | a/b | a | a | a | 8 |
| Tapashetti et al. 2014 | c | b | a | a | a/b | a | a | a | 7 |
| Goyal et al. 2014 | a | b | a | a | a/b | a | a | a | 8 |
| Kumar et al. 2014 | a | b | a | a | a/b | a | a | a | 8 |
| Kalburgi et al. 2014 | a | b | b | b | a/b | a | a | a | 6 |
| Pradeep et al. 2014 | a | a | a | a | a/b | a | a | a | 9 |
| Sharma et al. 2014 | c | b | a | a | a/b | a | a | a | 7 |
| Popławska-Kita et al. 2014 | a | b | c | a | a/b | a | a | a | 7 |
| Podzimek et al. 2015 | a | a | a | a | a/b | a | a | a | 9 |
| Yang et al. 2015 | a | a | a | a | a/b | a | a | a | 9 |
| Antonoglou et al. 2015 | a | b | b | a | a/b | a | a | a | 7 |
| Shi et al. 2015 | a | a | a | a | a/b | a | a | a | 9 |
| Ardila et al. 2015 | a | a | a | a | a/b | a | a | a | 9 |
| Gaddale et al. 2016 | a | b | a | a | a/b | a | a | a | 8 |
| Chandy et al. 2017 | a | b | a | a | a/b | a | a | a | 8 |
| Mysak et al. 2017 | a | b | b | a | a/b | a | a | a | 7 |
| Leira et al. 2018 | a | a | a | a | a/b | a | a | a | 9 |
| Ramich et al. 2018 | a | b | a | a | a/b | a | a | a | 8 |
| Martinez‐Herrera et al. 2018 | a | a | a | a | a/b | a | a | a | 9 |
| Isola et al. 2019 a | a | a | b | a | a/b | a | a | a | 8 |
| Maboudi et al. 2019 | a | b | b | a | a/b | a | a | a | 7 |
| Temelli et al. 2018 | a | a | a | a | a/b | a | a | a | 9 |
| Isola et al. 2019 b | a | a | b | a | a/b | a | a | a | 8 |
| Leite et al. 2020 | a | a | a | a | a/b | a | a | a | 9 |
| Isola et al. 2020 | a | a | a | a | a/b | a | a | a | 9 |
| Isola et al. 2020 c | a | a | a | a | a/b | a | a | a | 9 |
| Isola et al. 2020d | a | a | a | a | a/b | a | a | a | 9 |
| Leira et al. 2020 | a | a | a | a | a/b | a | a | a | 9 |
| Leira et al. 2020 | a | a | a | a | a/b | a | a | a | 9 |
| Altıngöz et al. (2021) | a | b | a | a | a/b | a | a | a | 8 |

Supplementary Table 8. Risk of Bias In Non-randomized Studies - of Interventions (ROBINS-I) tool

| Domain | 1 | 2 | 3 | 4 | 5 | 6 | 7 |  |
| --- | --- | --- | --- | --- | --- | --- | --- | --- |
| Study | Confounding | Selection | Classification | Deviations from interventions | Missing Data | Measurement of Outcomes | Selection of Reported Result | Overall |
| Ide et al. 2003 | Low | Low | Low | Low | Low | Low | Low | Low |
| Ide et al. 2004 | Low | Low | Low | Low | Low | Low | Low | Low |
| D’Aiuto et al. 2004a | Low | Low | Low | Low | Low | Low | Low | Low |
| D’Aiuto et al. 2004b | Low | Low | Low | Low | Low | Low | Low | Low |
| D’Aiuto et al. 2005b | Low | Low | Low | Low | Low | Low | Low | Low |
| Seinost et al. 2005 | Low | Low | Low | Low | Low | Low | Low | Low |
| Elter et al. 2006 | Low | Low | Low | Low | Low | Low | Low | Low |
| Marcaccini et al. 2009 | Low | Low | Low | Low | Low | Low | Low | Low |
| Pinho et al. 2009 | Low | Low | Low | Low | Low | Low | Low | Low |
| Behle et al. 2009 | Low | Low | Low | Low | Low | Low | Low | Low |
| Acharya et al. 2010 | Low | NI | Low | Low | Low | Low | Low | NI |
| Graziani et al. 2010 | Low | Low | Low | Low | Low | Low | Low | Low |
| Graziani et al. 2010 | Low | Low | Low | Low | Low | Low | Low | Low |
| Nagarale et al. 2010 | Low | Low | Low | Low | Low | Low | Low | Low |
| Shimada et al. 2010 | Low | Low | Low | Low | Low | Low | Low | Low |
| Kardesler et al. 2010 | Low | Low | Low | Low | Low | Low | Low | Low |
| Tamaki et al. 2010 | Low | Low | Low | Low | Low | Low | Low | Low |
| Vilela et al. 2011 | Low | Low | Low | Low | Low | Low | Low | Low |
| Patil et al. 2013 | Low | Low | Low | Low | Low | Low | Low | Low |
| Siqueira et al. 2013 | Serious | Low | Low | Low | Low | Low | Low | Serious |
| George et al. 2013 | Low | Low | Low | Low | Low | Low | Low | Low |
| Eickholz et al. 2013 | Low | Low | Low | Low | Low | Low | Low | Low |
| Mohan et al. 2014 | Low | Low | Low | Low | Low | Low | Low | Low |
| Altay et al. 2014 | Low | Low | Low | Low | Low | Low | Low | Low |
| Torumtay et al. 2015 | Low | Low | Low | Low | Low | Low | Low | Low |
| Gupta et al. 2015 | Low | Low | Low | Low | Low | Low | Low | Low |
| Duzagac et al. 2015 | Low | Low | Low | Low | Low | Low | Low | Low |
| Vargas-Villafuerte et al. 2016 | Low | Low | Low | Low | Low | Low | Low | Low |
| Žekonis et al. 2016 | Low | Low | Low | Low | Low | Low | Low | Low |
| Zuza et al. 2016 | Low | Low | Low | Low | Low | Low | Low | Low |
| Ling et al. 2016 | Low | Low | Low | Low | Low | Low | Low | Low |
| Kurgan et al. 2016 | Serious | Low | Low | Low | Low | Low | Low | Serious |
| Ertugrul et al. 2017 | Low | Low | Low | Low | NI | Low | Low | NI |
| Bozoglan et al. 2017 | Low | Low | Low | Low | Low | Low | Low | Low |
| de Souza et al. 2017 | Low | Low | Low | Low | Low | Low | Low | Low |
| Martinez-Herrera et al. 2017 | Low | Low | Low | Low | Low | Low | Low | Low |
| Taşdemir et al. 2017 | Low | Low | Low | Low | Low | Low | Low | Low |
| Taşdemir et al. 2018 | Low | Low | Low | Low | Low | Low | Low | Low |
| Fentoglu et al. 2017 | Low | Low | Low | Low | Low | Low | Low | Low |
| Mallapragada et al. 2017 | Low | Low | Low | Low | Low | Low | Low | Low |
| Morozumi et al. 2018 | Low | Low | Low | Low | Low | Low | Low | Low |
| Kocher et al. 2019 | Low | Low | Low | Low | Low | Low | Low | Low |
| Lobão et al. 2019 | Low | Low | Low | Low | Low | Low | Low | Low |
| Leite et al. 2019 | Low | Low | Low | Low | Low | Low | Low | Low |
| Caribé et al. 2019 | Low | Low | Low | Low | Low | Low | Low | Low |
| Luthra et al. 2019 | Low | Low | Low | Low | Low | Low | Low | Low |
| Preshaw et al. 2020 | Low | Low | Low | Low | Low | Low | Low | Low |
| Suvan et al. 2020 | Low | Low | Low | Low | Low | Low | Low | Low |

#

# Supplementary Figure 1. RoB2 Tool


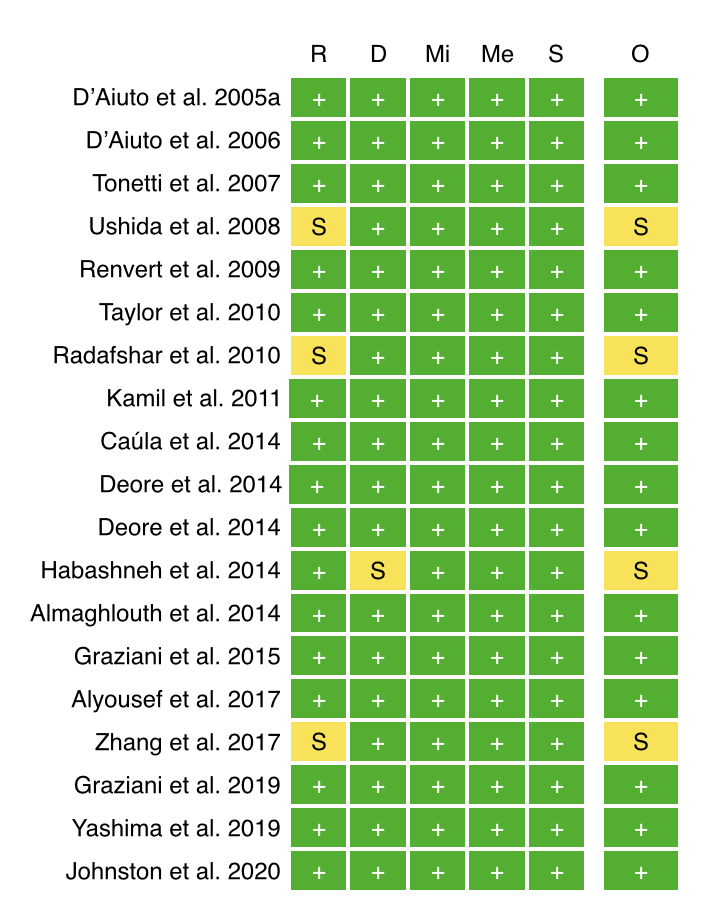


# Supplementary Table 9. Comparison between ROM and SMD results.

| **Variable** | **N** | **ROM** | **95% CI** | **I^2^ (%)** | **N** | **SMD** | **95% CI** | **I^2^ (%)** |
| --- | --- | --- | --- | --- | --- | --- | --- | --- |
| **H vs. CP** | | | | | | | | |
| CRP (mg/L) | 19 | **2.10** | 1.56; 2.83 | 98.5 | 19 | **1.28** | 0.88;1.67 | 93.6 |
| hs-CRP (mg//L) | 59 | **2.10** | 1.62; 2.55 | 97.4 | 59 | **2.04** | 1.72; 2.43 | 99.8 |
| **H vs. AgP** | | | | | | | | |
| CRP (mg/L) | 7 | **2.91** | 1.91; 4.44 | 98.6 | 7 | **3.18** | 1.59;4.77 | 97.6 |
| hs-CRP (mg//L) | 6 | **0.79** | 1.68; 4.65 | 83.6 | 6 | **0.89** | 0.27;1.50 | 91.7 |
| **H vs. P (CP and AgP combined)** | | | | | | | | |
| CRP (mg/L) | 24 | **2.06** | 1.64; 2.59 | 98.7 | 24 | **1.34** | 0.99; 1.70 | 95.1 |
| hs-CRP (mg//L) | 65 | **2.09** | 1.78: 2.47 | 99.8 | 65 | **1.95** | 1.54; 2.36 | 97.2 |
| **CP vs. AgP** | | | | | | | | |
| CRP (mg/L) | 5 | **1.56** | 1.15; 2.12 | 84.9 | 5 | **1.15** | 0.20; 2.10 | 93.2 |
| hs-CRP (mg//L) | 5 | 1.69 | 0.96; 2.98 | 77.5 | 5 | **0.84** | 0.33; 10.30 | 88.6 |

AgP - Aggressive Periodontitis; CP - Chronic Periodontitis; CRP - C-reactive protein; H - Healthy periodontium;  hs-CRP - high sensitivity CRP; ROM - Ratio of Mean; SMD - Standardized Mean Difference.

# Supplementary Table 10. Sensitivity analysis of type of studies using meta-regressions.

|  |  | **N** | **ROM** | **95% CI** | **I^2^** | ***p-value*** |
| --- | --- | --- | --- | --- | --- | --- |
| **CRP** | **Case-control studies with healthy and CP patients** | 17 | 2.16 | 1.56; 2.78 | 98.7 | - |
|  | **Interventional studies with healthy and CP patients** | 2 | - | - | - |  |
|  | **Case-control studies with healthy and AgP patients** | 6 | 3.14 | 1.97; 5.01 | 98.8 | - |
|  | **Interventional studies with healthy and AgP patients** | 1 | - | - | - |  |
|  | **Case-control studies with healthy and periodontitis patients** | 22 | 2.10 | 1.65; 2.67 | 98.8 | 0.318 |
|  | **Interventional studies with healthy and periodontitis patients** | 2 | 1.80 | 1.47; 2.18 | 0 |  |
|  | **Case-control studies with CP and AgP patients** | 3 | 1.65 | 1.28; 2.13 | 76.6 | - |
|  | **Interventional studies with CP and AgP patients** | 2 | - | - | - |  |
| **hs-CRP** | **Case-control studies with healthy and periodontitis patients** | 51 | 2.09 | 1.74; 2.51 | 99.8 | 0.448 |
|  | **Interventional studies with healthy and periodontitis patients** | 8 | 1.75 | 1.16; 2.64 | 75.7 |  |
|  | **Case-control studies with healthy and AgP patients** | 6 | 2.79 | 1.68; 4.65 | 83.6 | - |
|  | **Interventional studies with healthy and AgP patients** | 0 | - | - | - |  |
|  | **Case-control studies with healthy and periodontitis patients** | 57 | 2.14 | 1.80; 2.55 | 99.8 | 0.361 |
|  | **Interventional studies with healthy and periodontitis patients** | 8 | 1.75 | 1.16; 2.64 | 75.7 |  |
|  | **Case-control studies with CP and AgP patients** | 5 | 1.69 | 0.96; 2.98 | 77.5 | - |
|  | **Interventional studies with CP and AgP patients** | 0 | - | - | - |  |

CRP- C-reactive protein; hs-CRP - high-sensitivity C-reactive protein; CI - Confidence Interval; N - Number of studies included; SE - Standard Error.

# Supplementary Table 11. Sensitivity analysis of the risk of bias.

|  |  |  | **N** | **Estimate** | **95% CI** | ***I^2^ (%)*** |
| --- | --- | --- | --- | --- | --- | --- |
| **CRP** | **Healthy vs CP** | Total | 19 | 2.10 | 1.10; 2.83 | 98.5 |
|  |  | Low RoB | 17 | 1.93 | 1.30; 2.88 | 66.3 |
|  | **Healthy vs AgP** | Total | 7 | Not applicable - articles included are of low RoB | | |
|  |  | Low RoB | 7 |  |  |  |
|  | **CP vs AgP** | Total | 5 | Not applicable - articles included are of low RoB | | |
|  |  | Low RoB | 5 |  |  |  |
| **hs-CRP** | **Healthy vs CP** | Total | 58 | 2.06 | 1.73; 2.45 | 99.8 |
|  |  | Low RoB | 54 | 2.01 | 1.68; 2.40 | 99.8 |
|  |  | Moderate | 4 | 2.14 | 1.64; 2.79 | 82.0 |
|  |  | Serious | 1 | - | - | - |
|  | **Healthy vs AgP** | Total | 6 | Not applicable - All articles are of low RoB | | |
|  |  | Low RoB | 6 |  |  |  |
|  | **CP vs AgP** | Total | 6 | Not applicable - All articles are of low RoB | | |
|  |  | Low RoB | 6 |  |  |  |

CRP- C-reactive protein; hs-CRP - high-sensitivity C-reactive protein; CI - Confidence Interval; N - Number of studies included; PD - periodontitis; RoB - Risk of bias; SE - Standard Error

# Supplementary Figure 2. CRP of Healthy Periodontium versus Chronic Periodontitis individuals. Subgroup analysis according to the CRP method.


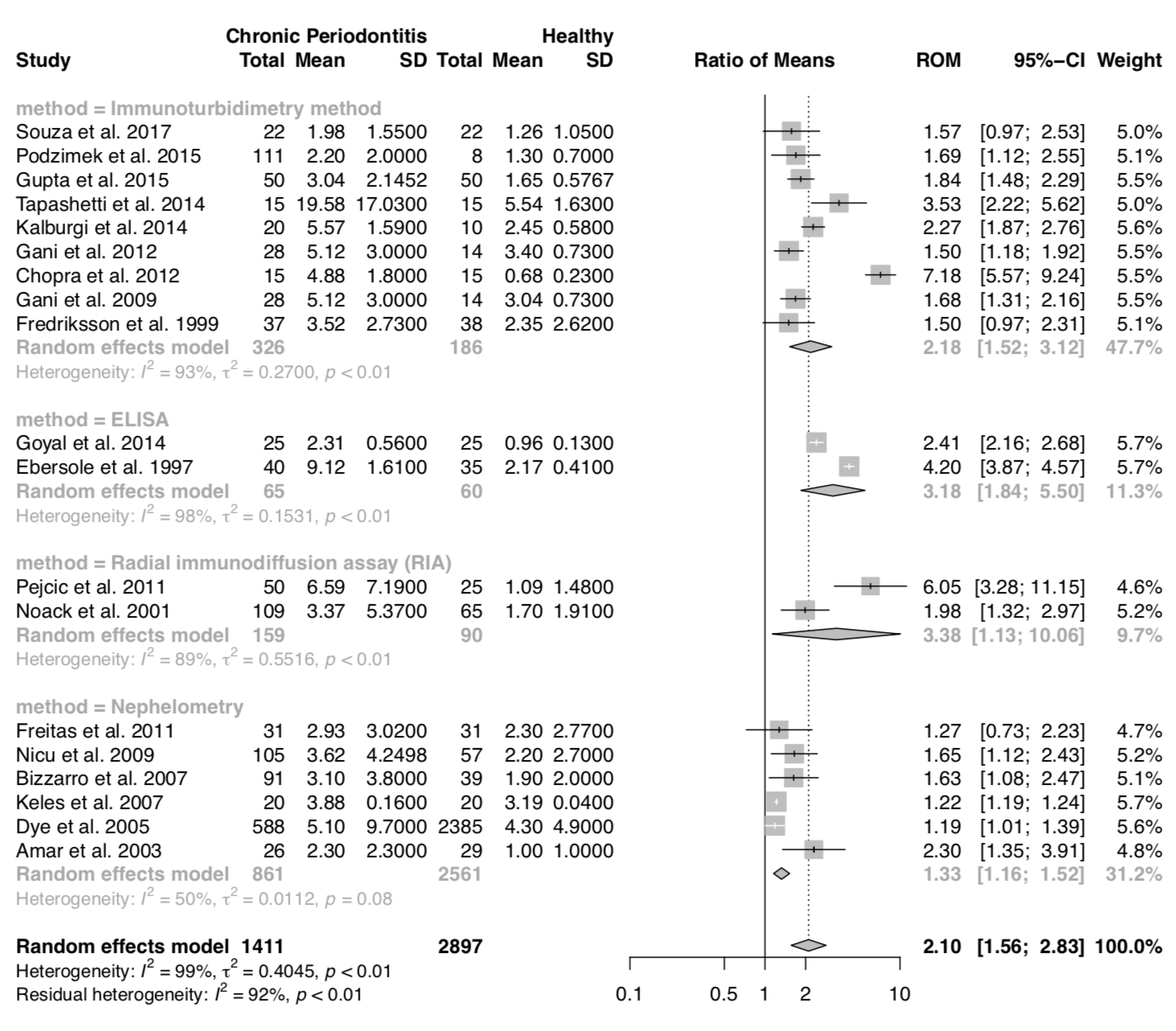


# Supplementary Figure 3. CRP of Healthy Periodontium versus Chronic Periodontitis individuals. Subgroup analysis according to the study type.


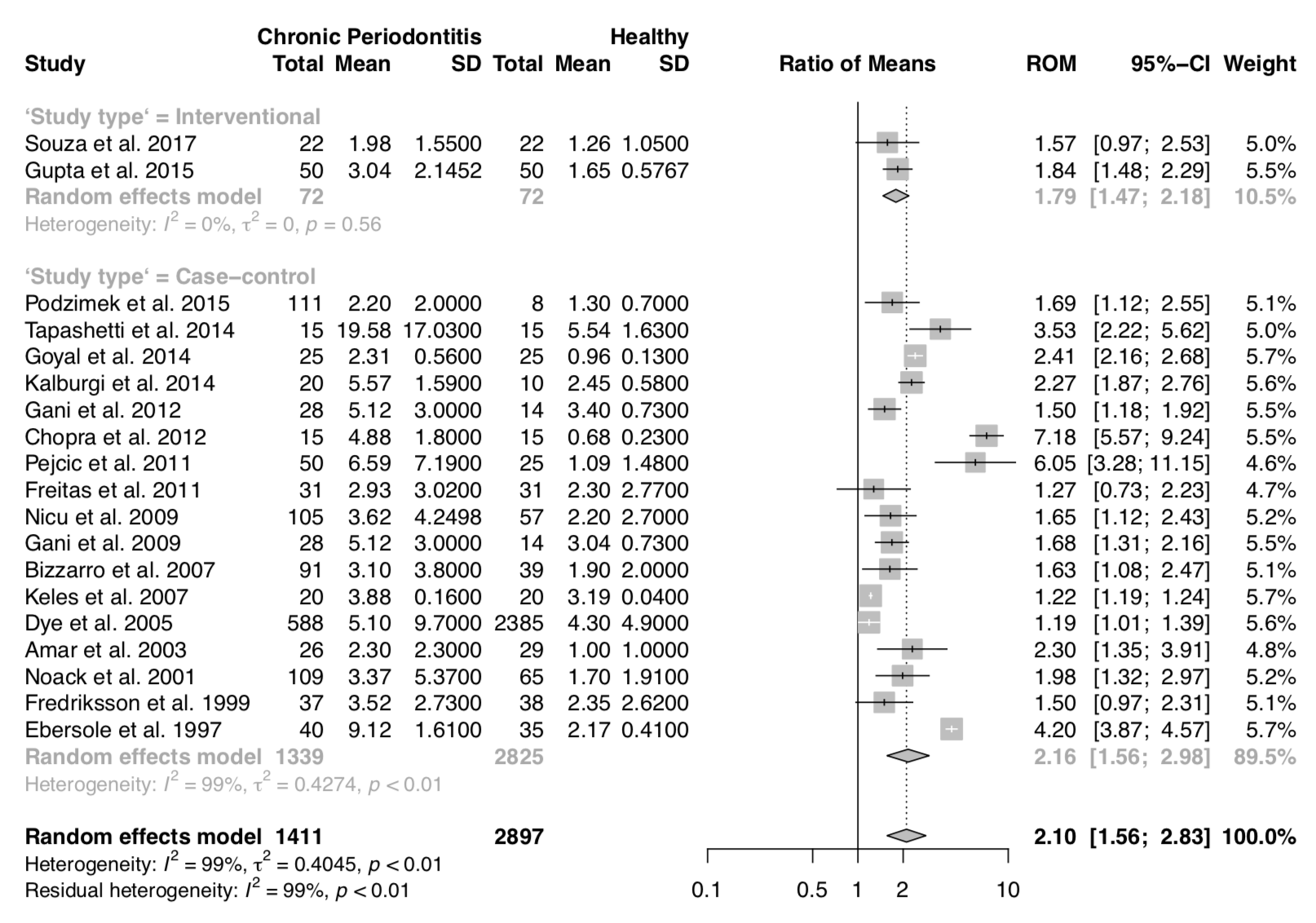


# Supplementary Figure 4. hs-CRP of Healthy Periodontium versus Chronic Periodontitis individuals. Subgroup analysis according to the CRP method.


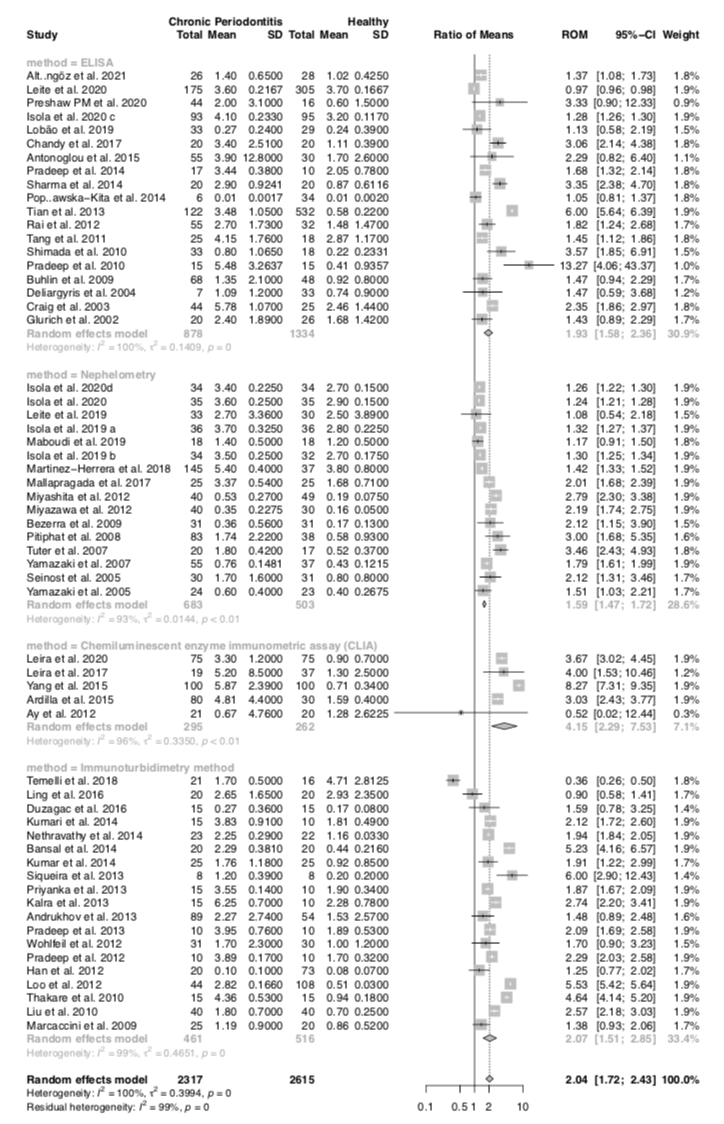


# Supplementary Figure 5. hs-CRP of Healthy Periodontium versus Chronic Periodontitis individuals. Subgroup analysis according to the type of study.


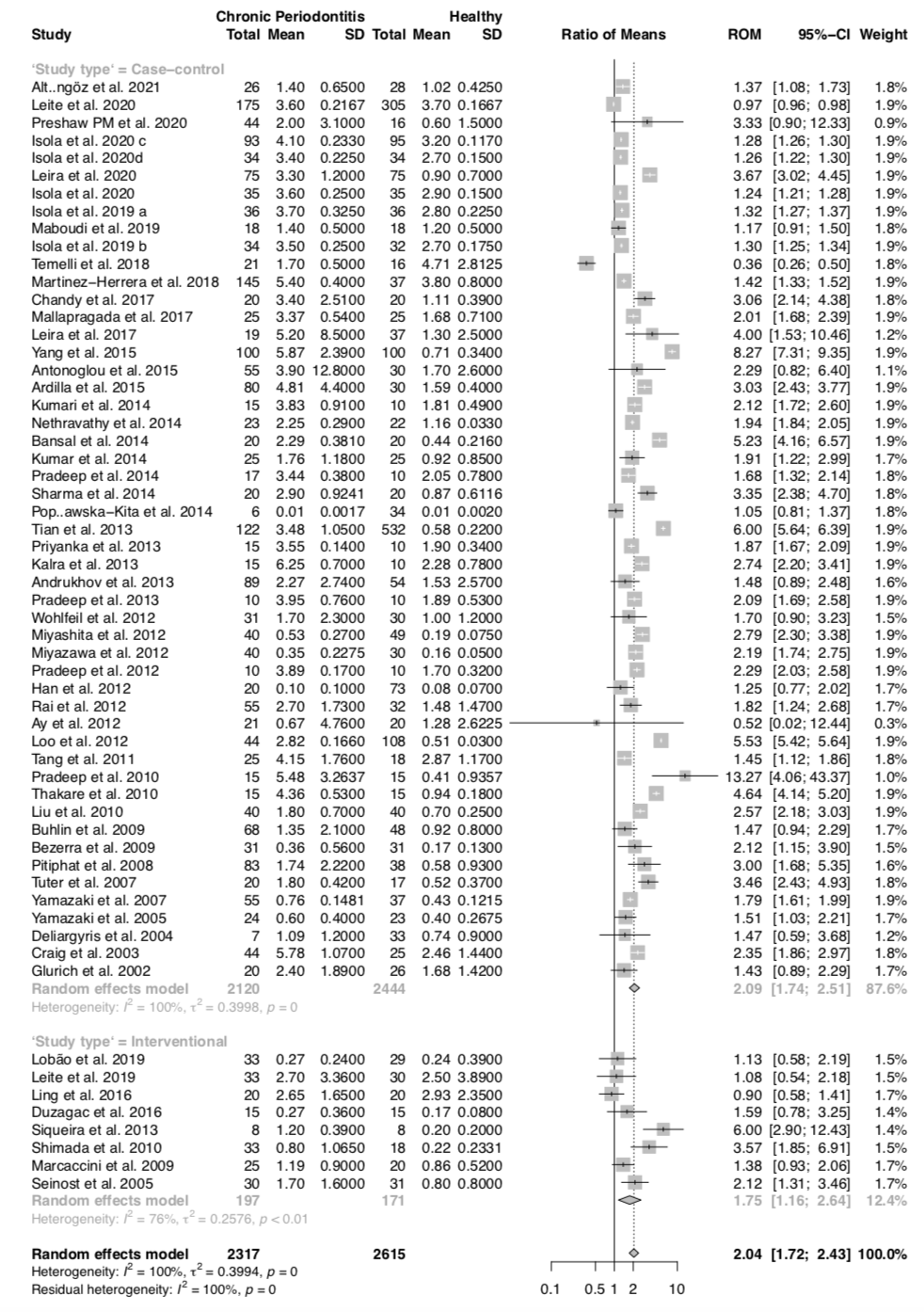


# Supplementary Figure 6. CRP of Healthy Periodontium versus Aggressive Periodontitis individuals.


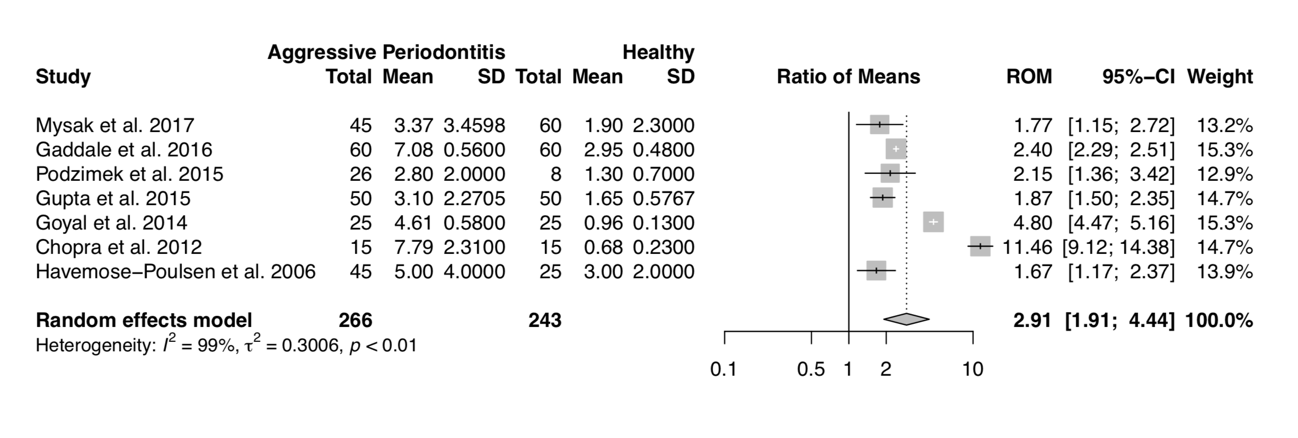


# Supplementary Figure 7. hs-CRP of Healthy Periodontium versus Aggressive Periodontitis individuals. Subgroup analysis according to the type of study.


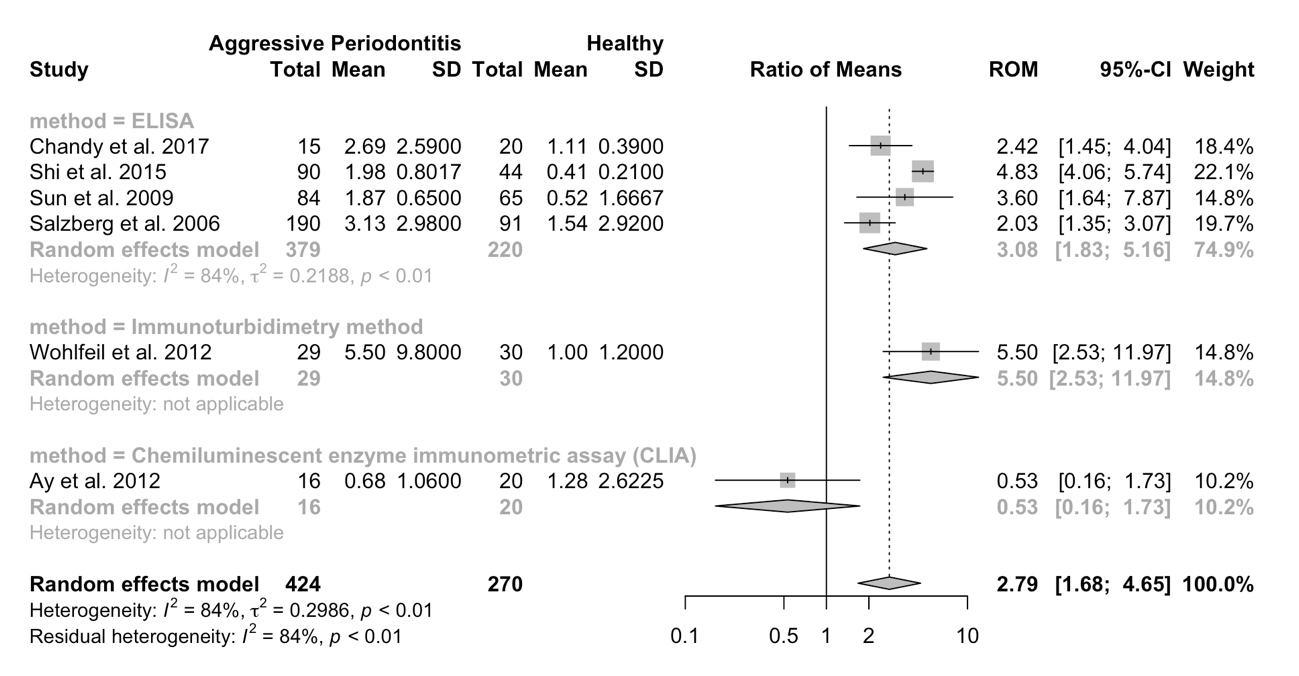


# Supplementary Figure 8. CRP of Healthy Periodontium versus Periodontitis individuals. Subgroup analysis according to the CRP method.


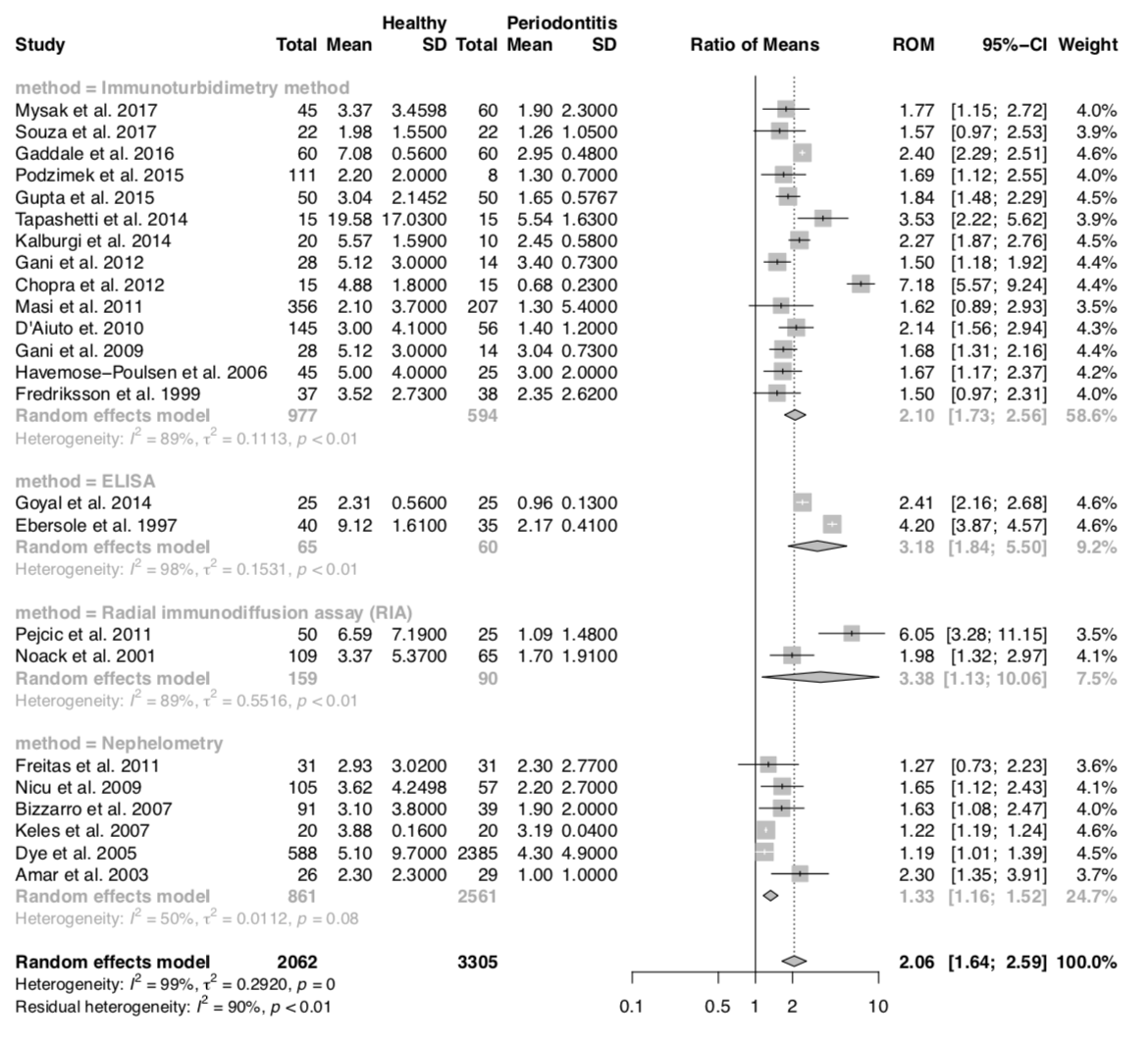


# Supplementary Figure 9. hs-CRP of Healthy Periodontium versus Periodontitis individuals. Subgroup analysis according to the study type.


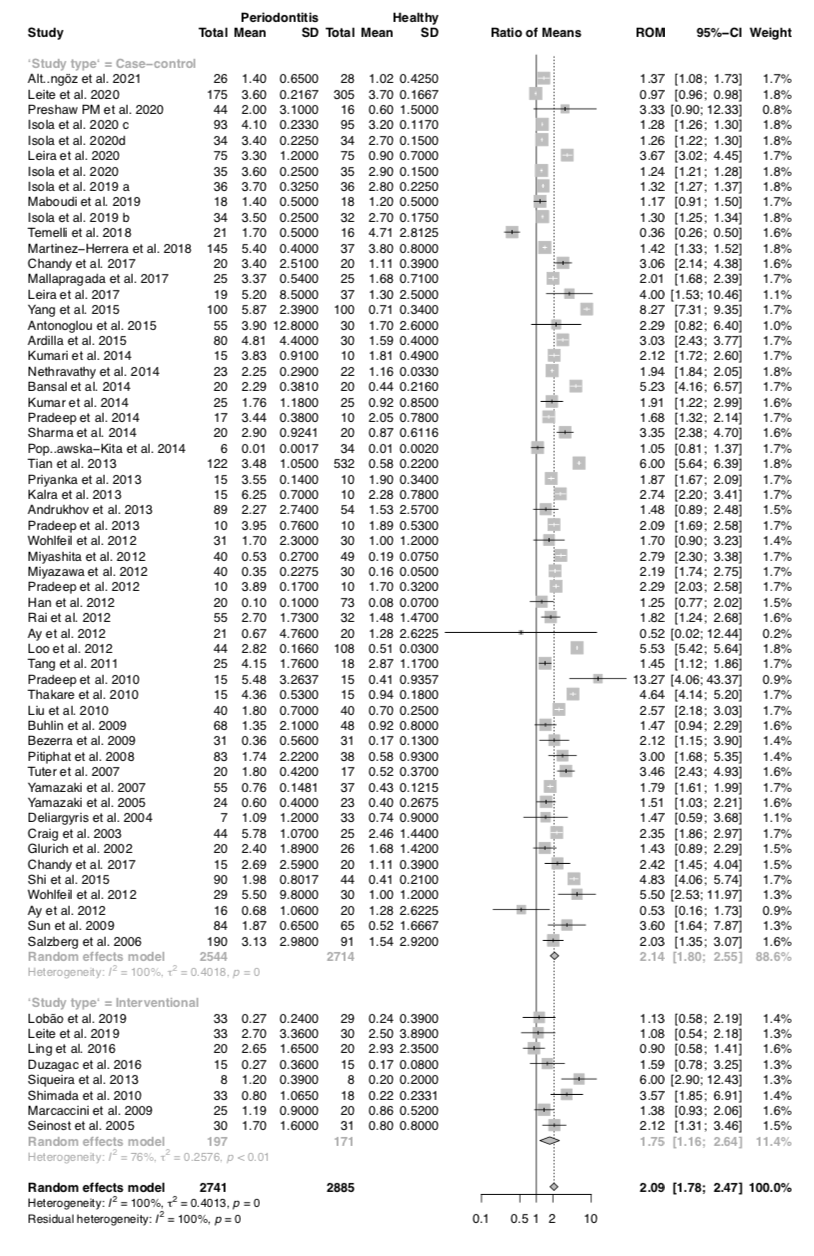


# Supplementary Figure 10. hs-CRP of Healthy Periodontium versus Periodontitis individuals. Subgroup analysis according to the hs-CRP method.


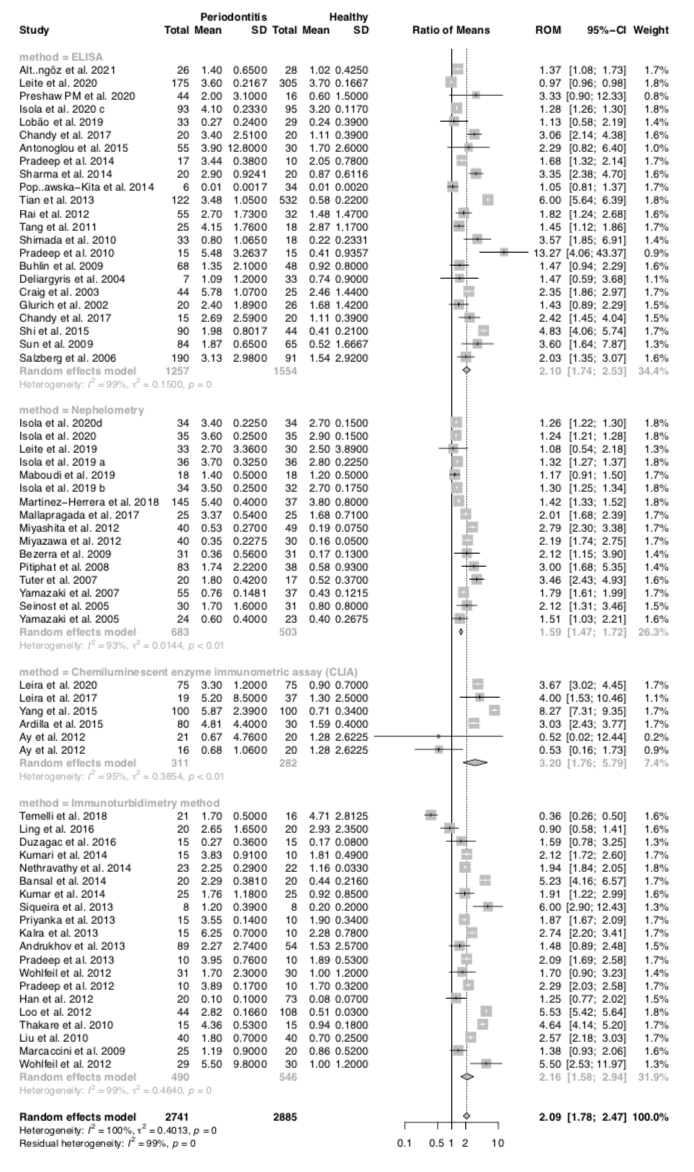


# Supplementary Figure 11. CRP of Chronic Periodontitis versus Aggressive Periodontitis individuals. Subgroup analysis according to the CRP method.


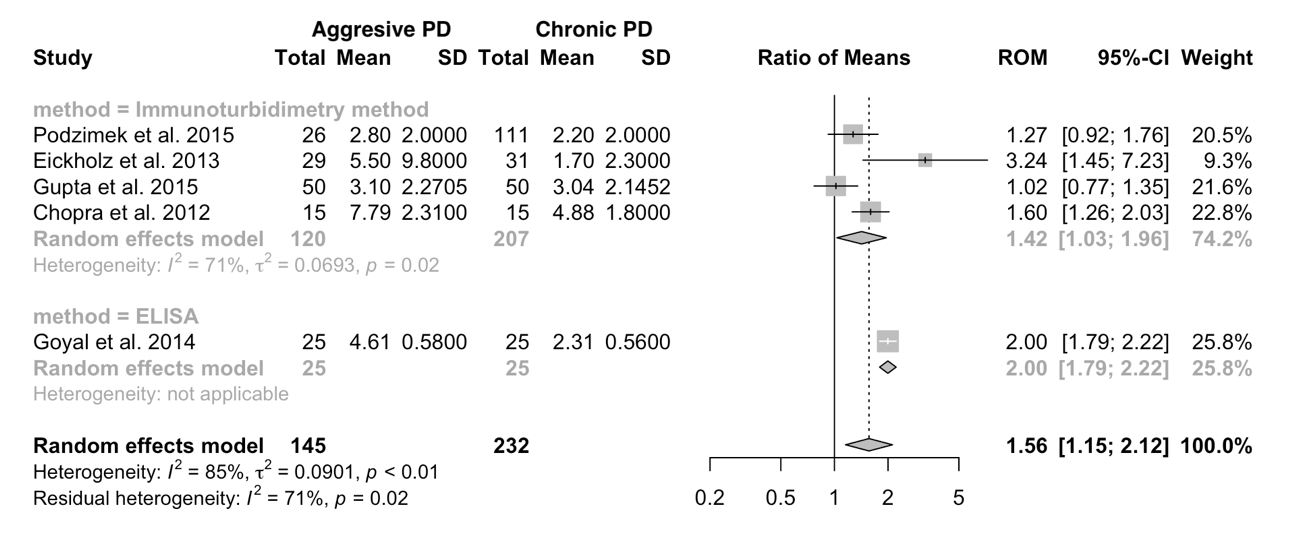


# Supplementary Figure 12. CRP of Chronic Periodontitis versus Aggressive Periodontitis individuals. Subgroup analysis according to the study type.


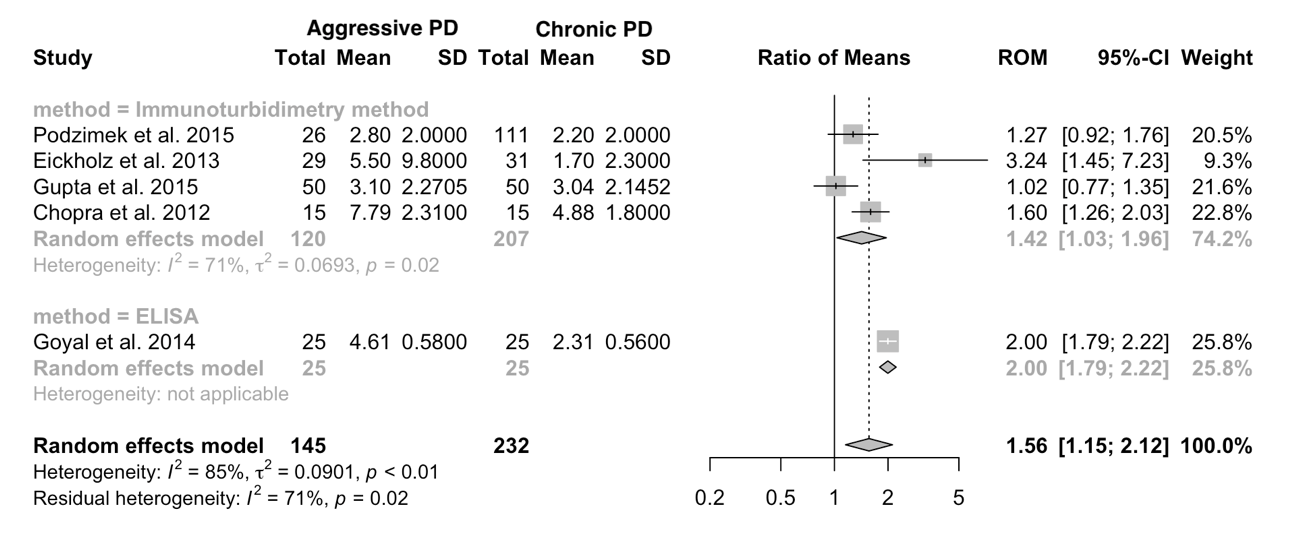


# Supplementary Figure 13. hs-CRP of Chronic Periodontitis versus Aggressive Periodontitis individuals.


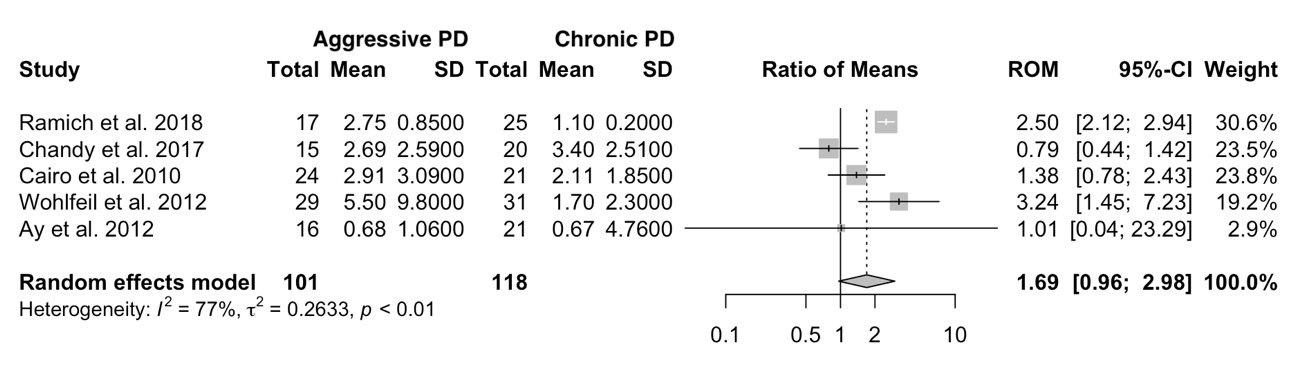


# Supplementary Figure 14. Funnel plot and Egger’s test for CRP of Healthy Periodontium versus Chronic Periodontitis individuals.

#
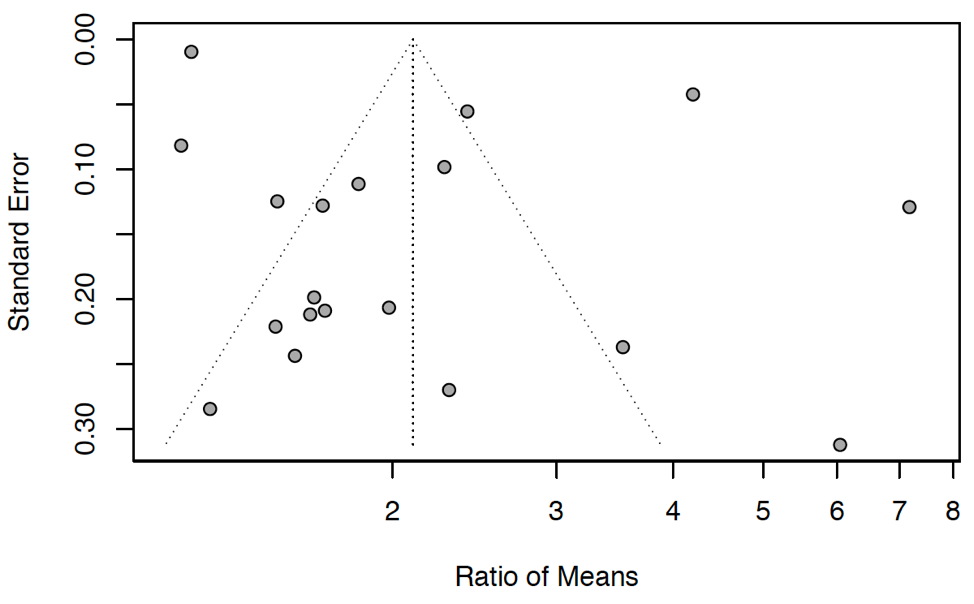


# Supplementary Figure 15. Funnel plot and Egger’s test for CRP of Healthy Periodontium versus Periodontitis individuals.


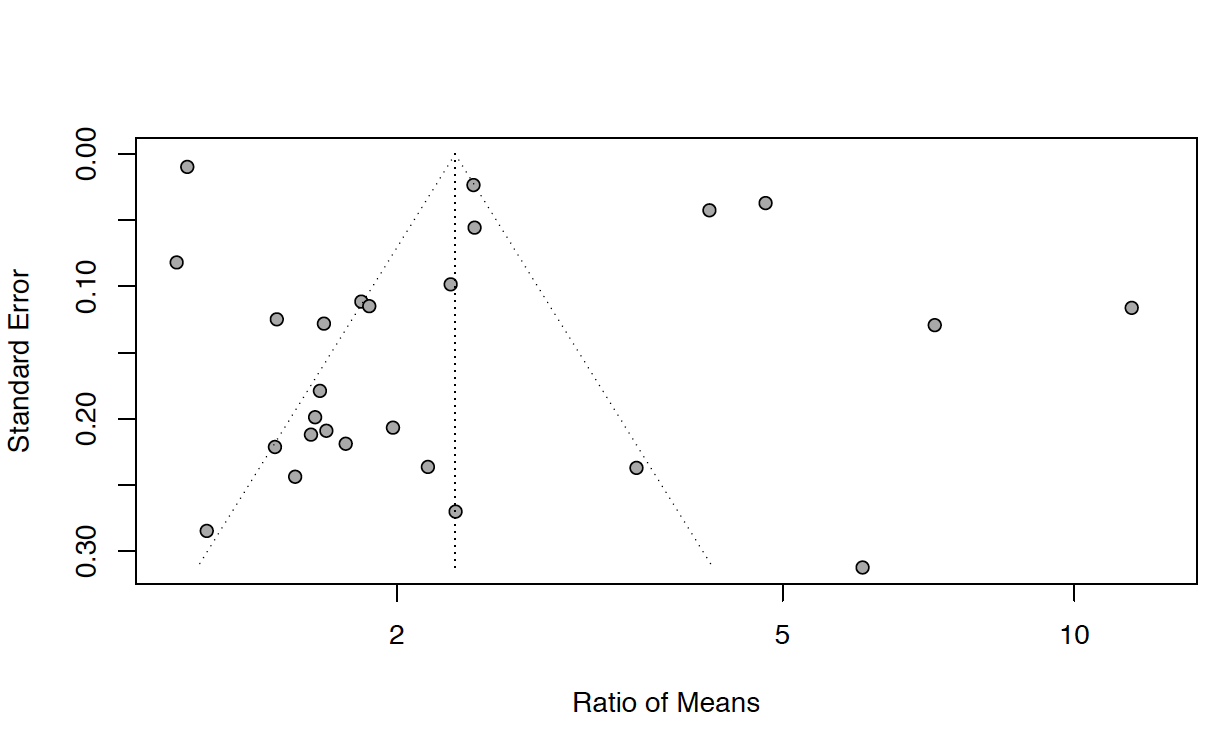


# Supplementary Figure 16. Funnel plot and Egger’s test for hs-CRP of Healthy Periodontium versus Chronic Periodontitis individuals.


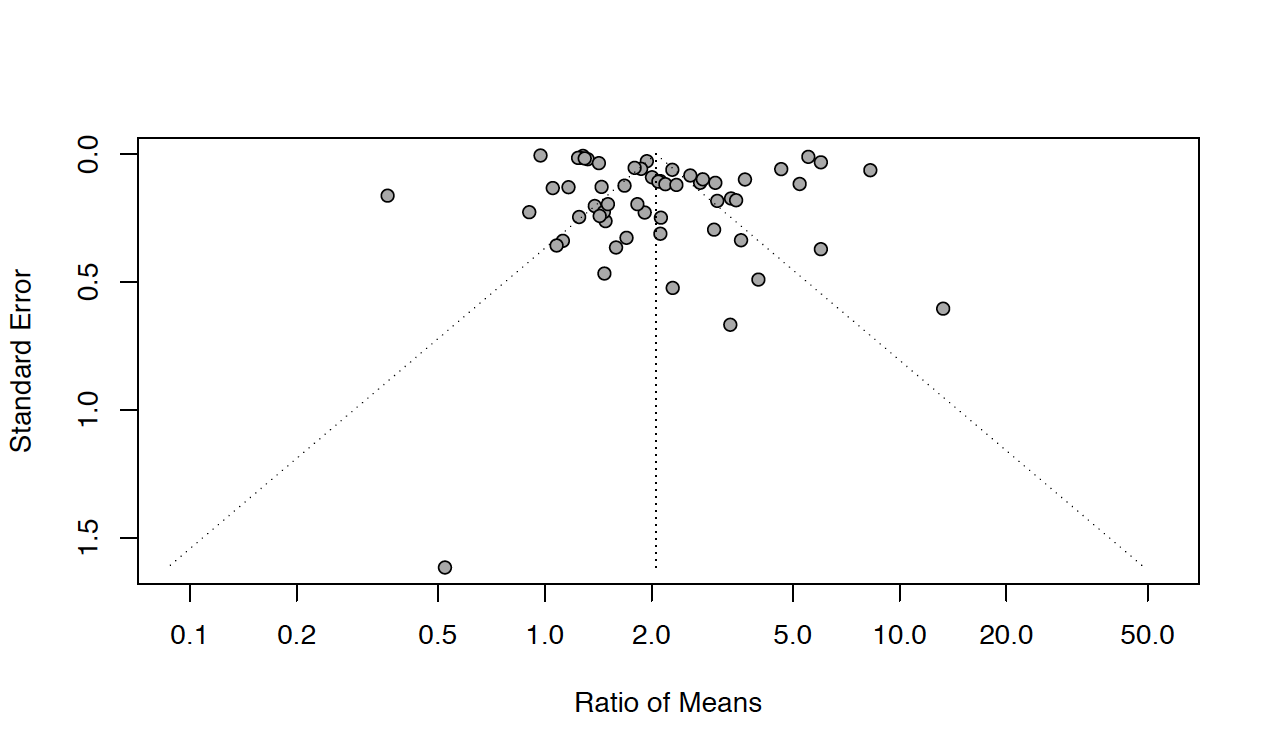


# Supplementary Figure 17. Funnel plot and Egger’s test for hs-CRP of Healthy Periodontium versus Periodontitis individuals.


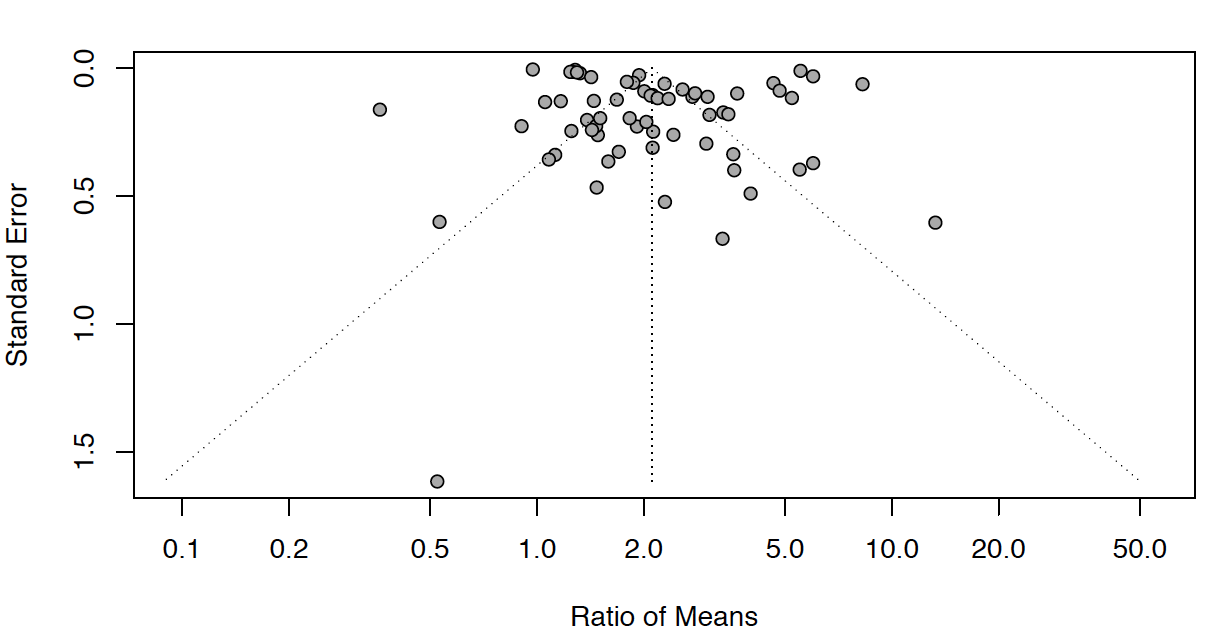


Supplementary Table 12. Sensitivity analysis regarding the type of meta-analytical approach for non-intensive treatment studies

| Non-intensive treatment | | | | | | | | |
| --- | --- | --- | --- | --- | --- | --- | --- | --- |
| Day | n | ROM | 95% CI | I^2^ (%) | n | SMD | 95% CI | I^2^ (%) |
| 1 | 4 | 0.98 | 0.82; 1.17 | 0.0 | 4 | -0.05 | -0.32; 0.20 | 0.0 |
| 7 | 6 | 0.95 | 0.81; 1.1 | 5.7 | 6 | -0.08 | -0.29; 0.19 | 5.7 |
| 30 | 12 | 0.75 | 0.67; 0.86 | 83.9 | 12 | -0.53 | -0.92; -0.14 | 83.0 |
| 42 | 3 | 0.84 | 0.66; 1.08 | 0.0 | 3 | -0.20 | -0.52; 0.12 | 0.0 |
| 60 | 13 | 0.84 | 0.76; 0.94 | 77.5 | 13 | -0.48 | -0.77; -0.19 | 77.5 |
| 90 | 23 | 0.71 | 0.64; 0.78 | 73.0 | 23 | -0.71 | -1.01; -0.41 | 82.9 |
| 180 | 11 | 0.55 | 0.45; 0.66 | 88.5 | 11 | -1.25 | -1.83; -0.67 | 92.4 |

Supplementary Table 13. Sensitivity analysis regarding the type of meta-analytical approach for intensive treatment studies

| Intensive treatment | | | | | | | | |
| --- | --- | --- | --- | --- | --- | --- | --- | --- |
| Day | n | ROM | 95% CI | I^2^ (%) | n | SMD | 95% CI | I^2^ (%) |
| 1 | 16 | 3.05 | 2.12; 4.38 | 95.5 | 16 | 1.86 | 1.25; 2.46 | 91.8 |
| 7 | 9 | 1.25 | 1.04; 1.5 | 73.8 | 9 | 0.39 | 0.08; 0.71 | 63.8 |
| 30 | 10 | 0.96 | 0.86; 1.07 | 4.9 | 10 | -0.02 | -0.21; 0.17 | 10.4 |
| 42 | 4 | 0.93 | 0.56; 1.52 | 34.7 | 4 | -0.04 | -0.32; 0.24 | 16.7 |
| 60 | 4 | 0.96 | 0.87; 1.05 | 0.0 | 4 | -0.06 | -0.28; 0.16 | 0.0 |
| 90 | 13 | 0.87 | 0.78; 0.97 | 82.8 | 13 | -0.34 | -0.61; 0.07 | 63.5 |
| 180 | 6 | 1.04 | 0.89; 1.21 | 64.7 | 6 | 0.02 | -0.34; 0.38 | 35.9 |

Supplementary Table 14. Sensitivity analysis regarding the presence of studies without low risk of bias for non-intensive treatment studies

| Non-intensive treatment | | | | | | | | |
| --- | --- | --- | --- | --- | --- | --- | --- | --- |
|  | Global | | | | Low risk of bias | | | |
| Day | n | ROM | 95% CI | I^2^ (%) | n | ROM | 95% CI | I^2^ (%) |
| 1 | 4 | 0.98 | 0.82-1.17 | 0.0 | 3 | 0.97 | 0.81-1.17 | 0.0 |
| 7 | 6 | 0.95 | 0.81-1.1 | 5.7 | 6 | 0.95 | 0.81-1.10 | 17.9 |
| 30 | 12 | 0.75 | 0.67-0.86 | 83.9 | 11 | 0.76 | 0.66-0.87 | 62.2 |
| 42 | 3 | 0.84 | 0.66-1.08 | 0.0 | 3 | 0.84 | 0.66-1.08 | 0.0 |
| 60 | 13 | 0.84 | 0.76-0.94 | 77.5 | 12 | 0.84 | 0.75-0.94 | 83.8 |
| 90 | 23 | 0.71 | 0.64-0.78 | 73.0 | 20 | 0.69 | 0.62-0.76 | 61.0 |
| 180 | 11 | 0.55 | 0.45-0.66 | 88.5 | 9 | 0.59 | 0.48-0.73 | 87.9 |

Appendix S32. Sensitivity analysis regarding the presence of studies without low risk of bias for intensive treatment studies

| Intensive treatment | | | | | | | | |
| --- | --- | --- | --- | --- | --- | --- | --- | --- |
|  | Global | | | | Low risk of bias | | | |
| Day | n | ROM | 95% CI | I^2^ (%) | n | ROM | 95% CI | I^2^ (%) |
| 1 | 16 | 3.05 | 2.12-4.38 | 95.5 | 16 | 3.57 | 2.43-5.26 | 95.9 |
| 7 | 9 | 1.25 | 1.04-1.50 | 73.8 | 9 | 1.25 | 1.04-1.50 | 73.8 |
| 30 | 10 | 0.96 | 0.86-1.07 | 4.9 | 10 | 0.94 | 0.86-1.03 | 0.0 |
| 42 | 4 | 0.93 | 0.56-1.52 | 34.7 | 4 | 0.93 | 0.56-1.52 | 34.7 |
| 60 | 4 | 0.92 | 0.71-1.18 | 0.0 | 4 | 0.92 | 0.71-1.18 | 0.0 |
| 90 | 13 | 0.87 | 0.78-0.97 | 82.8 | 13 | 0.87 | 0.78-0.97 | 82.8 |
| 180 | 6 | 0.98 | 0.82-1.17 | 43.4 | 6 | 0.98 | 0.82-1.17 | 43.4 |

Appendix S33. Sensitivity analysis for the influence of the presence of smoking participants using meta-regression in non-intensive and intensive treatment studies

| Non-intensive treatment | | | | Intensive treatment | | | |
| --- | --- | --- | --- | --- | --- | --- | --- |
| Day | Estimate | SE | p-value | Day | Estimate | SE | p-value |
| 1 | 0.26 | 0.62 | 0.67 | 1 | 2.22 | 1.39 | 0.11 |
| 7 | -0.17 | 0.63 | 0.79 | 7 | 0.01 | 0.95 | 1.00 |
| 30 | 1.40 | 0.94 | 0.14 | 30 | -0.09 | 0.45 | 0.82 |
| 42 | -* | - | - | 42 | 1.50 | 0.92 | 0.10 |
| 60 | 0.35 | 0.24 | 0.15 | 60 | 2.75 | 2.06 | 0.18 |
| 90 | 0.33 | 0.54 | 0.54 | 90 | -0.18 | 0.78 | 0.81 |
| 180 | 0.70 | 0.39 | 0.07 | 180 | -1.13 | 0.94 | 0.23 |

* All studies had non-smokers included
